# A Comprehensive Genomic Analysis Constructs miRNA–mRNA Interaction Network in Hepatoblastoma

**DOI:** 10.3389/fcell.2021.655703

**Published:** 2021-08-06

**Authors:** Tong Chen, Linlin Tian, Jianglong Chen, Xiuhao Zhao, Jing Zhou, Ting Guo, Qingfeng Sheng, Linlin Zhu, Jiangbin Liu, Zhibao Lv

**Affiliations:** ^1^Department of General Surgery, Shanghai Children’s Hospital, Shanghai Jiao Tong University, Shanghai, China; ^2^Department of Microbiology, Faculty of Basic Medical Sciences, Guilin Medical University, Guilin, China

**Keywords:** hepatoblastoma, miRNA, mRNA, PPI, TF

## Abstract

Hepatoblastoma (HB) is a rare disease but nevertheless the most common hepatic tumor in the pediatric population. For patients with advanced HB, the prognosis is dismal and there are limited therapeutic options. Multiple microRNAs (miRNAs) were reported to be involved in HB development, but the miRNA–mRNA interaction network in HB remains elusive. Through a comparison between HB and normal liver samples in the GSE131329 dataset, we detected 580 upregulated differentially expressed mRNAs (DE-mRNAs) and 790 downregulated DE-mRNAs. As for the GSE153089 dataset, the first cluster of differentially expressed miRNAs (DE-miRNAs) were detected between fetal-type tumor and normal liver groups, while the second cluster of DE-miRNAs were detected between embryonal-type tumor and normal liver groups. Through the intersection of these two clusters of DE-miRNAs, 33 upregulated hub miRNAs, and 12 downregulated hub miRNAs were obtained. Based on the respective hub miRNAs, the upstream transcription factors (TFs) were detected via TransmiR v2.0, while the downstream target genes were predicted via miRNet database. The intersection of target genes of respective hub miRNAs and corresponding DE-mRNAs contributed to 250 downregulated candidate genes and 202 upregulated candidate genes. Gene Ontology (GO) and Kyoto Encyclopedia of Genes and Genomes (KEGG) analyses demonstrated the upregulated candidate genes mainly enriched in the terms and pathways relating to the cell cycle. We constructed protein–protein interaction (PPI) network, and obtained 211 node pairs for the downregulated candidate genes and 157 node pairs for the upregulated candidate genes. Cytoscape software was applied for visualizing the PPI network and respective top 10 hub genes were identified using CytoHubba. The expression values of hub genes in the PPI network were subsequently validated through Oncopression database followed by quantitative real-time polymerase chain reaction (qRT-PCR) in HB and matched normal liver tissues, resulting in six significant downregulated genes and seven significant upregulated genes. The miRNA–mRNA interaction network was finally constructed. In conclusion, we uncover various miRNAs, TFs, and hub genes as potential regulators in HB pathogenesis. Additionally, the miRNA–mRNA interaction network, PPI modules, and pathways may provide potential biomarkers for future HB theranostics.

## Introduction

Hepatoblastoma (HB) is a rare disease with an annual incidence of 1.5 cases per million children per year (Spector and Birch, 2012). Nevertheless, it is the predominant hepatic tumor in the pediatric population (Schnater et al., 2003). The past three decades have witnessed a consistently increasing incidence of HB (Linabery and Ross, 2008). Surgical resection and chemotherapy have dramatically improved the prognosis for HB children, with the 3-years event-free survival (EFS) > 80% (Aronson et al., 2014). However, there are limited therapeutic strategies for advanced HB children, with the 3-years EFS of only 34% (Semeraro et al., 2013). In addition, patient survivors may suffer severe side effects of chemotherapeutic or immunosuppressive agents. Therefore, there is an urgent need to unveil the molecular mechanisms underlying this rare tumor in order to identify novel biomarkers for therapeutic tailoring.

MicroRNAs (miRNAs) are ∼22 nucleotide non-coding RNAs that post-transcriptionally suppress messenger RNAs (mRNAs) expression (Lou et al., 2019). Through base-pairing interactions with mRNAs, miRNAs play crucial roles in proliferation (Roy et al., 2017), apoptosis (Liu et al., 2020), epithelial-mesenchymal transition (Weng et al., 2019), and autophagy (Kuang et al., 2020) of human cells. Moreover, the dysregulated expression of miRNA is associated with the pathogenesis of various human tumors, including HB (Cui et al., 2019b). In the context of HB, miR-193a-5p promotes proliferative, migrative, and invasive properties of HB through targeting DPEP1 and augmenting PI3K/AKT/mTOR signaling pathway (Cui et al., 2019a); miR-492 serves as an endogenous tumor-promoting factor to induce proliferation, anchorage-independent growth, migrative and invasive properties of HB cells by targeting CD44, and high level of miR-492 expression is correlated with high-risk or aggressive HB (von Frowein et al., 2018); miR-21 enhances apoptosis in HB cells through targeting ASPP2 and augmenting ASPP2/p38 signaling pathway (Liu et al., 2019). In other words, the intimate relationship between altered expression of certain miRNA and its target gene has been uncovered in HB. Transcription factors (TFs) are endogenous proteins that regulate the transcription process of mRNAs or miRNAs. The function of TFs can be either oncogenic or tumor suppressive depending on context (Lambert et al., 2018). Recently, multiple TFs have been demonstrated to modulate the aggressive phenotype and cellular process in HB development (Zhang et al., 2019; Nakra et al., 2020; Wagner et al., 2020).

In recent years, high-throughput technologies have enabled us to identify the key genes, miRNAs, and TFs in the initiation and progression of human tumors. To date, there has been a scarce number of integrated genome-wide studies on HB (Zhang L. et al., 2018; Aghajanzadeh et al., 2020) via research on several cases or one dataset. To gain a better understanding of the underlying mechanisms behind HB, this study aimed to explore the miRNA–mRNA interaction network, TFs, and biological pathways involved in HB through comprehensive bioinformatic approaches.

## Materials and Methods

### Data Retrieval and Extraction

HB-related data were obtained from the Gene Expression Omnibus (GEO^1^) database portal via the keyword “hepatoblastoma.” The dataset was included when all four items of the following criteria were met: (1) there were both HB and normal liver samples; (2) the dataset had miRNA or mRNA transcriptome data; (3) data for all samples were completely presented; (4) HB and normal liver samples could be clearly distinguished using principal component analysis (PCA). After screening, we chose one mRNA dataset (accession number: GSE131329) and one miRNA dataset (accession number: GSE153089) for further analysis. GSE131329 (Hiyama et al., 2019), consisting of 53 HB samples and 14 normal liver samples, was analyzed via GPL6244 platform (Affymetrix Human Gene 1.0 ST Array). GSE153089 (Honda et al., 2020), comprising of 30 HB samples and 14 normal liver samples, was analyzed via GPL21572 platform (Affymetrix Multispecies miRNA-4 Array). General information of the two datasets used for the present study is shown in Supplementary Table 1.

The GSE153089 dataset included nine specimens from metastatic tumor, 21 specimens from primary tumor (11 fetal subtypes and 10 embryonal subtypes), and 14 specimens from surrounding normal liver (Honda et al., 2020). Due to the lack of metastatic tumor samples in the GSE131329 dataset (Hiyama et al., 2019), we excluded all specimens from metastatic tumor in the GSE153089 dataset before further analysis. The remaining specimens in the GSE153089 dataset were subsequently divided into three groups, namely, normal surrounding liver, fetal-type tumor, and embryonal-type tumor groups. Each patient in the GSE153089 dataset possessed no more than one specimen from the same group except for patient 7. There were two fetal-type tumor specimens for patient 7 (Sample ID: 25F-1 and 25F-2), one (Sample ID: 25F-2) of which was randomly excluded for further analysis. Detailed information of samples in the GSE153089 dataset used for the present study is listed in Supplementary Table 2.

### Screening of Differentially Expressed miRNAs and Differentially Expressed mRNAs

Raw data files (^∗^.CEL) of GSE153089 and GSE131329 were imported using the *oligo* (Carvalho and Irizarry, 2010) R package. The data were sequentially filtered, background corrected, log base 2 transformed, and normalized. Based on the platform annotation information, gene symbol was obtained via conversion of the probe. If one gene symbol corresponded to two or more probes, the mean expression level of these corresponding mRNAs or miRNAs was treated as the final expression value. Before and after clustering and removing outliers, we detected the distribution patterns of HB and normal liver samples via PCA. DE-mRNAs and DE-miRNAs were then detected using the *limma* R package (17). An adjusted *P* < 0.05 and | log2FC| > 1 indicated statistical significance. Benjamini–Hochberg (BH) method was used to adjust the *P* value. Regarding the GSE131329 dataset, DE-mRNAs were obtained based on the comparison between HB and normal liver samples. As for the GSE153089 dataset, the first cluster of DE-miRNAs were detected between fetal-type tumor and normal liver groups, while the second cluster of DE-miRNAs were detected between embryonal-type tumor and normal liver groups. Through the intersection of these two clusters of DE-miRNAs, the upregulated or downregulated hub miRNAs were obtained.

### Prediction of Potential TFs and Target Genes of Hub miRNAs

Based on the hub miRNAs, we predicted the upstream TFs via TransmiR v2.0 (Tong et al., 2019), an easy-accessible public tool integrating experimentally verified TF-miRNA regulatory relationships from the publications. The Cytoscape software was subsequently utilized to visualize TF-miRNA regulatory relationships (Shannon et al., 2003). In addition, miRNet database was used for the prediction of the downstream target genes of hub miRNAs (Fan et al., 2016).

### Gene Ontology and Kyoto Encyclopedia of Genes and Genomes Analyses

To further explore functional annotation of the candidate genes, we performed Gene Ontology (GO) and Kyoto Encyclopedia of Genes and Genomes (KEGG) analyses via the *clusterProfiler* R package (Yu et al., 2012). GO terms consisted of biological process (BP), cellular component (CC), and molecular function (MF). An adjusted *P* < 0.05 was considered significantly enriched, and BH method was used to adjust the *P* value.

### Protein–Protein Interaction Network

To unveil the relationships between the candidate genes, we established the PPI network via the STRING database (Szklarczyk et al., 2015). PPI pairs were considered significant with a combined score ≥ 0.4. Cytoscape software was subsequently applied to visualize the network (Shannon et al., 2003). On the basis of the degree obtained through Cytoscape plugin CytoHubba (Chin et al., 2014), top 10 hub genes were detected in the PPI network.

### Hub Genes Verification Through Oncopression Database

We applied Oncopression database^2^ to validate expression levels of top 10 up-regulated hub genes and top 10 down-regulated hub genes. Oncopression is a web-based integrated gene expression profile using single sample normalization method UPC (Lee and Choi, 2017).

### Tissue Samples

Hepatoblastoma and matched normal liver tissue samples from eight children undergoing surgical excision for primary HB were obtained from our hospital between 2014 and 2019. None of the patients received adjuvant radiotherapy or chemotherapy prior to surgery. Tissues were stored at −80°C immediately after harvest until further use. The pathological diagnosis of the tissue adjacent to each frozen tissue specimen was confirmed by at least two independent pathologists.

### RNA Extraction and Quantitative Real-Time Polymerase Chain Reaction

Total RNAs were isolated from the tissues using TRIzol reagent (Life Technologies, Carlsbad, CA, United States). Total mRNA was subsequently reverse-transcribed to produce complementary DNA (cDNA) using TaKaRa reverse transcription kit (TaKaRa Bio, Shiga, Japan). The SYBR Green fluorescence system (Roche, IN, United States) was used, and mRNA qRT-PCR was performed using a quantitative mRNA kit (TaKaRa Bio, Shiga, Japan). Based on the 2^–ΔΔ*Ct*^ method, the relative mRNA levels were normalized to GAPDH mRNA levels. All primers were synthesized by Sangon (Shanghai, China). The sequence of primers is summarized in Supplementary Table 3.

### Statistical Analysis

We conducted data analysis and visualization using R software (version 3.6.3) and GraphPad Prism (version 8.0.1). The expression levels of mRNAs or miRNAs between groups in the datasets were compared via a moderated *t*-test. For differential expression analysis of mRNAs or miRNAs in the datasets, a *P* value < 0.05 and | log2FC| > 1 were considered statistically significant. The mRNA expression levels of hub genes in HB and matched normal liver tissues from our hospital were statistically analyzed by a paired Student *t* test, and *P* values below 0.05 were considered significant.

## Results

### Hub miRNAs Identification

**FIGURE 1:**
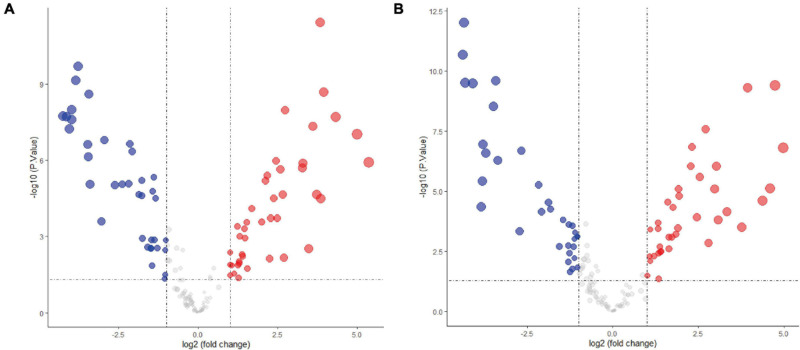
Differentially expressed microRNAs analysis of the GSE153089 dataset. **(A)** DE-miRNAs between fetal-type tumor and normal liver samples were visualized via volcano plot. **(B)** DE-miRNAs between embryonal-type tumor and normal liver samples were visualized via volcano plot. Red points representing up-regulation; blue points indicating down-regulation; gray points representing normal expression. DE-miRNAs, differentially expressed microRNAs.

The expression levels of fetal-type tumor and normal liver samples in the GSE153089 dataset prior to and after normalization are shown (Supplementary Figures 1A,B). PCA results before and after removing outliers (GSM4633970, GSM4633988, and GSM4633998) are also presented (Supplementary Figures 1C,D). Based on the differential expression analysis, we detected 41 upregulated DE-miRNAs and 36 downregulated DE-miRNAs, which are presented via volcano plot in Figure 1A. In addition, these DE-miRNAs between fetal-type tumor and normal liver samples were regarded as the first cluster of DE-miRNAs.

The expression values of embryonal-type tumor and normal liver samples in the GSE153089 dataset prior to and after normalization are shown (Supplementary Figures 2A,B). PCA results before and after removing outliers (GSM4633970 and GSM4633988) are also presented (Supplementary Figures 2C,D). Through the differential expression analysis, we detected 37 upregulated DE-miRNAs and 33 downregulated DE-miRNAs, which are presented via volcano plot in Figure 1B. Additionally, these DE-miRNAs between embryonal-type tumor and normal liver samples were regarded as the second cluster of DE-miRNAs.

**FIGURE 2:**
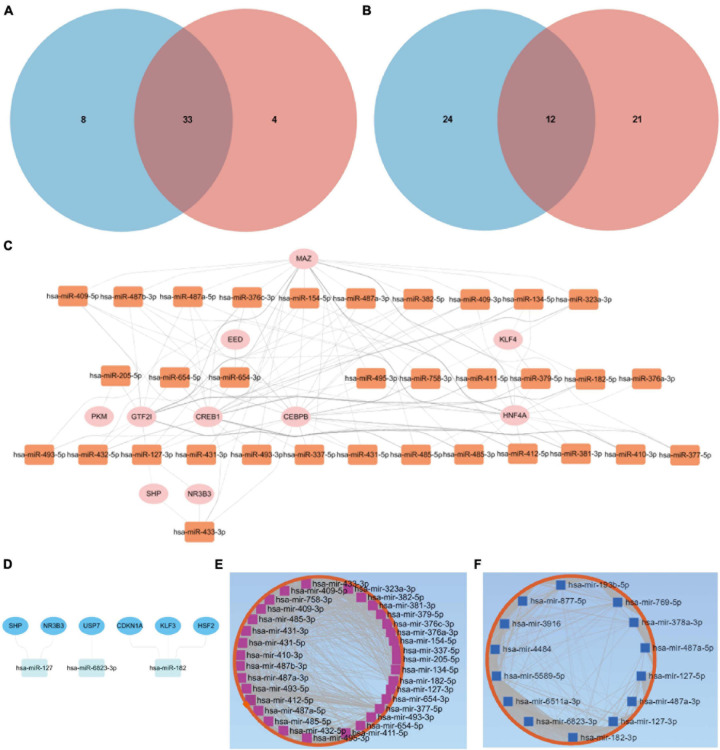
Putative TFs and target genes of the hub miRNAs. **(A)** The intersection of the two clusters of upregulated DE-miRNAs. **(B)** The intersection of the two clusters of downregulated DE-miRNAs. Putative TFs for **(C)** upregulated or **(D)** downregulated hub miRNAs. **(E)** Upregulated or **(F)** downregulated hub miRNA-target gene network. DE-miRNAs, differentially expressed microRNAs; TFs, transcription factors.

Through the intersection of the aforementioned two clusters of DE-miRNAs, a total of 33 upregulated DE-miRNAs and 12 downregulated hub miRNAs were obtained (Figures 2A,B). Detailed information of respective hub miRNAs is also listed (Supplementary Tables 4,5).

### TFs and Target Genes Predicted by Hub miRNAs

As for the upregulated hub miRNAs, the predicted TFs included HNF4A, GTF2I, CEBPB, CREB1, MAZ, NR3B3, SHP, KLF4, PKM, and EED (Figure 2C). Regarding the downregulated hub miRNAs, the predicted TFs included NR3B3, SHP, CDKN1A, KLF3, USP7, and HSF2 (Figure 2D). Detailed information of the TFs predicted for the upregulated or downregulated DE-miRNAs is also listed (Supplementary Tables 6,7). Apart from the predicted TFs, we also predicted 5,772 target genes of the upregulated hub miRNAs and 5,600 target genes of the downregulated hub miRNAs. Upregulated hub miRNA-target gene network and downregulated hub miRNA-target gene network are presented in Figures 2E,F, respectively.

### DE-mRNAs Identification

**FIGURE 3:**
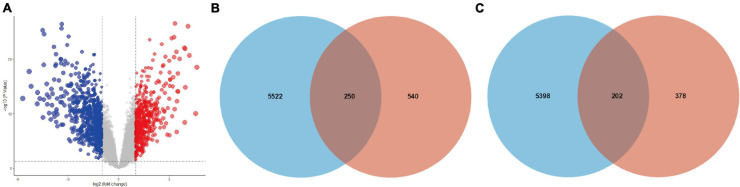
Intersection of target genes of hub miRNAs and corresponding DE-mRNAs. **(A)** DE-mRNAs between HB and normal liver samples in the GSE131329 dataset were visualized using volcano plot. Red points representing up-regulation; blue points indicating down-regulation; gray points representing normal expression. The intersection of target genes of **(B)** upregulated or **(C)** downregulated hub miRNAs and corresponding DE-mRNAs. DE-mRNAs, differentially expressed mRNAs.

The expression levels of all samples in the GSE131329 dataset before and after normalization are visualized in Supplementary Figures 3A,B, respectively. PCA results prior to and after excluding the outlier (GSM3770543) are also shown (Supplementary Figures 3C,D). We then obtained 580 upregulated DE-mRNAs and 790 downregulated DE-mRNAs, which are presented via volcano plot in Figure 3A. The detailed information of these respective DE-mRNAs is listed (Supplementary Tables 8,9). Subsequently, we intersected the target genes of upregulated hub miRNAs and downregulated DE-mRNAs, resulting in a total of 250 downregulated candidate genes (Figure 3B). In addition, the intersection of target genes of downregulated hub miRNAs and upregulated DE-mRNAs resulted in 202 upregulated candidate genes (Figure 3C). Detailed information of these downregulated and upregulated candidate genes is also listed (Supplementary Tables 10, 11).

### Functional Annotation Enrichment Analyses

**FIGURE 4:**
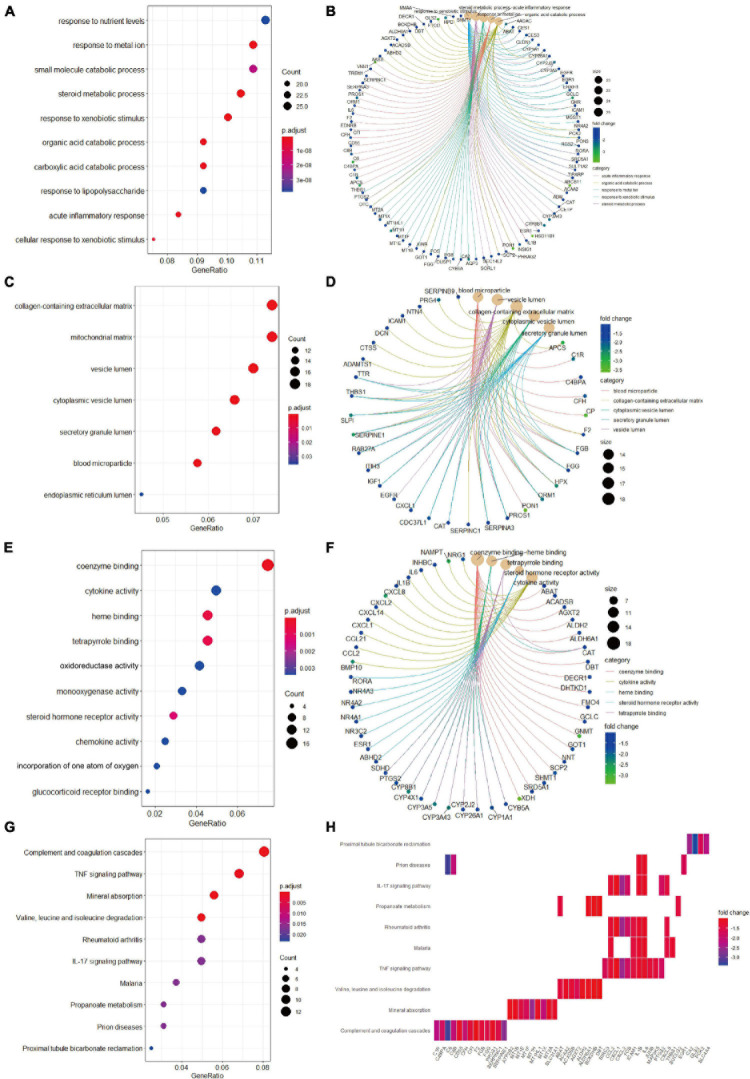
Gene Ontology terms and KEGG pathway enrichment analyses of the downregulated candidate genes. **(A)** The enriched GO-BP terms based on downregulated candidate genes. **(B)** The downregulated candidate genes and their enriched GO-BP terms. **(C)** The enriched GO-CC terms based on downregulated candidate genes. **(D)** The downregulated candidate genes and their enriched GO-CC terms. **(E)** The enriched GO-MF terms based on downregulated candidate genes. **(F)** The downregulated candidate genes and their enriched GO-MF terms. **(G)** KEGG pathway analysis showing the enriched pathways based on downregulated candidate genes. **(H)** Heatmap showing specific downregulated candidate genes and their enriched pathways. BP, biological process; CC, cellular component; GO, Gene Ontology; KEGG, Kyoto Encyclopedia of Genes and Genomes; MF, molecular function.

Biological process analysis indicated that enriched GO terms for downregulated candidate genes included response to nutrient levels, response to metal ion, small molecule catabolic process, and steroid metabolic process (Figure 4A). CC analysis showed that the candidate genes were markedly enriched in collagen-containing extracellular matrix, mitochondrial matrix, vesicle lumen, cytoplasmic vesicle lumen, secretory granule lumen, and blood microparticle (Figure 4C). In the process of MF analysis, the candidate genes were markedly enriched in coenzyme binding, cytokine activity, heme binding, tetrapyrrole binding, and oxidoreductase activity (Figure 4E). The complex relationships between these candidate genes and their related GO terms were visualized using the *cnetplot* R package (Figures 4B,D,F). Moreover, KEGG analysis identified complement and coagulation cascades, TNF signaling pathway, mineral absorption, and valine, leucine and isoleucine degradation as markedly enriched pathways (Figure 4G). Besides, the enriched pathways and their associated candidate genes were unveiled, which are shown as heatmap in Figure 4H.

**FIGURE 5:**
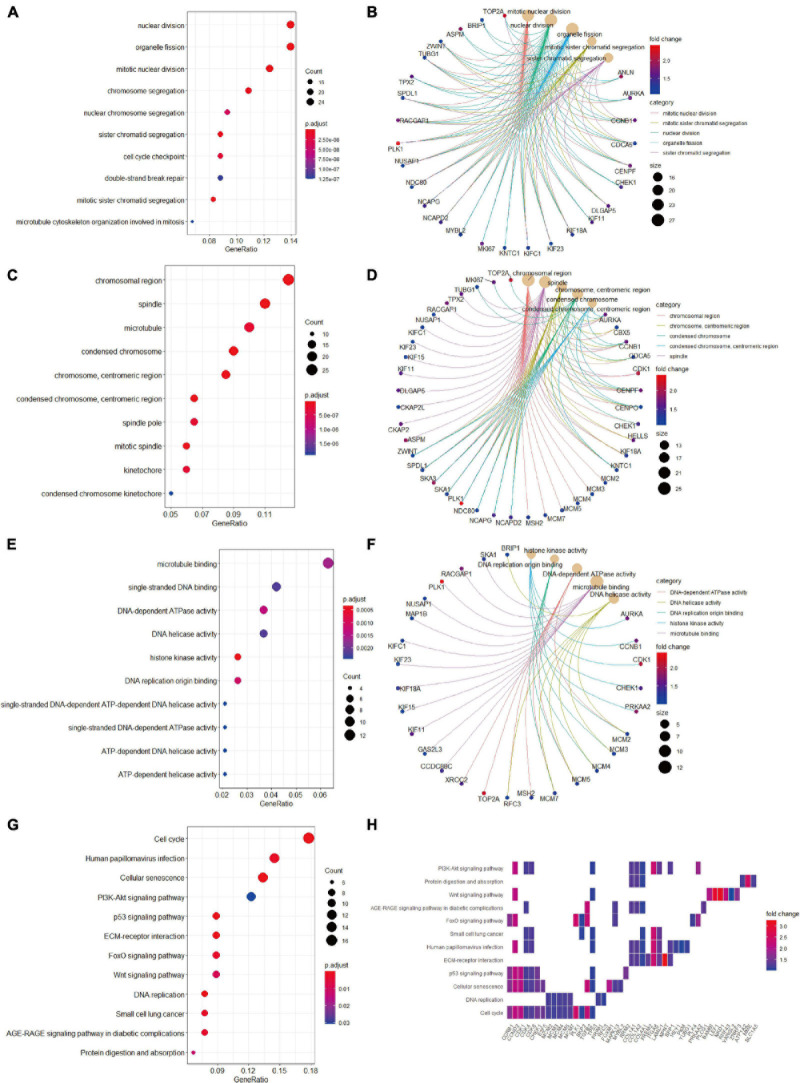
Gene Ontology terms and KEGG pathway enrichment analyses of the upregulated candidate genes. **(A)** The enriched GO-BP terms based on upregulated candidate genes. **(B)** The upregulated candidate genes and their enriched GO-BP terms. **(C)** The enriched GO-CC terms based on upregulated candidate genes. **(D)** The upregulated candidate genes and their enriched GO-CC terms. **(E)** The enriched GO-MF terms based on upregulated candidate genes. **(F)** The upregulated candidate genes and their enriched GO-MF terms. **(G)** KEGG pathway analysis showing the enriched pathways based on upregulated candidate genes. **(H)** Heatmap showing specific upregulated candidate genes and their enriched pathways. BP, biological process; CC, cellular component; GO, Gene Ontology; KEGG, Kyoto Encyclopedia of Genes and Genomes; MF, molecular function.

We then conducted GO terms analysis based on the upregulated candidate genes. BP analysis revealed that nuclear division, organelle fission, mitotic nuclear division, and chromosome segregation served as the top enriched GO terms (Figure 5A). CC analysis identified chromosomal region, spindle, microtubule, and condensed chromosome as significantly enriched GO terms (Figure 5C). In addition, MF analytic results revealed microtubule binding, single-stranded DNA binding, DNA-dependent ATPase activity, DNA helicase activity, and histone kinase activity as markedly enriched GO terms (Figure 5E). The complex relationships between the aforementioned enriched GO terms and their associated candidate genes are also shown (Figures 5B,D,F). KEGG analysis identified cell cycle, cellular senescence and PI3K-AKT signaling as significantly enriched pathways (Figure 5G), and the associations of these pathways and their related candidate genes were visualized using heatmap (Figure 5H).

### PPI Network Construction and Hub Genes Screening

**FIGURE 6:**
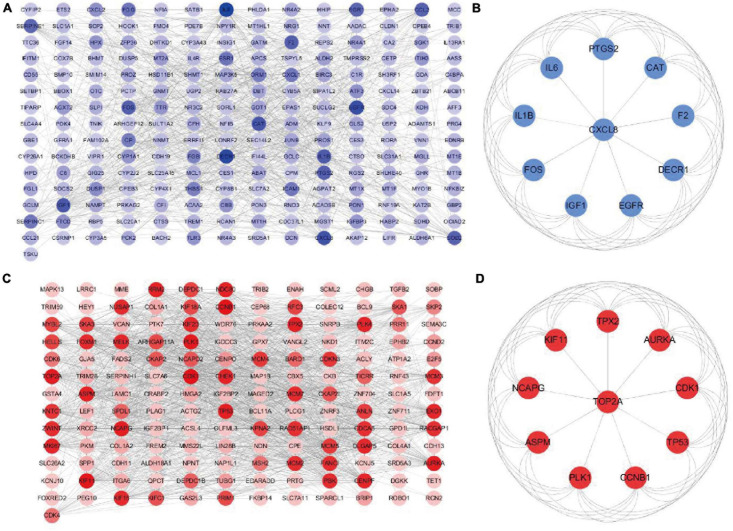
Construction of PPI network based on downregulated or upregulated candidate genes. PPI network of **(A)** downregulated or **(B)** upregulated candidate genes. **(C)** The top 10 hub genes of downregulated candidate genes based on the node degree. **(D)** The top 10 hub genes of upregulated candidate genes based on the node degree. PPI, protein–protein interactions.

The downregulated or upregulated candidate genes were loaded into the STRING database, resulting in the construction of respective PPI network. A total of 211 node pairs were obtained for the downregulated candidate genes (Figure 6A), while 157 node pairs were obtained for the upregulated candidate genes (Figure 6C). The node pairs were input into Cytoscape software to visualize genes in respective PPI network. The respective top 10 hub genes were detected via Cytoscape plugin CytoHubba (Figures 6B,D). Specifically, the top 10 upregulated hub genes were CDK1, CCNB1, KIF11, PLK1, NCAPG, TOP2A, AURKA, TP53, ASPM, and TPX2, while the top 10 downregulated hub genes included IL6, DECR1, EGFR, CXCL8, CAT, IGF1, IL1B, F2, PTGS2, and FOS.

### Hub Genes Verification via Oncopression Database

**FIGURE 7:**
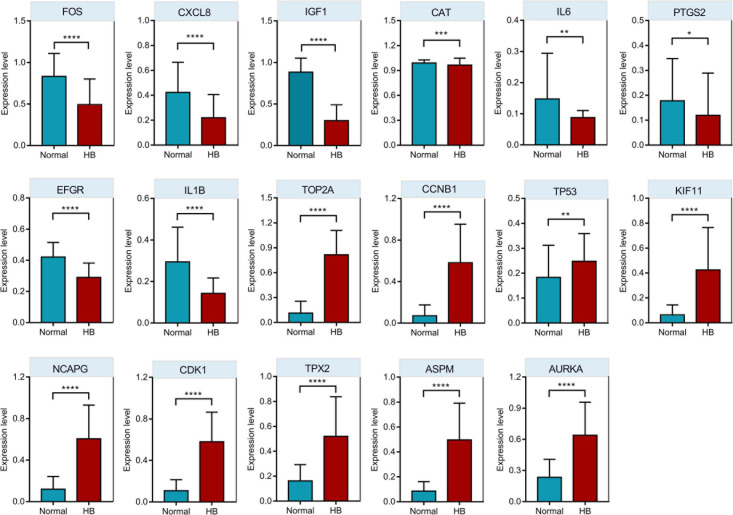
The comparison of hub genes expression levels in HB and normal liver tissue samples from Oncopression database. After UPC-normalization, the expression levels range from 0 to 1 where 0 and 1 indicate no expression and the highest expression, respectively. *P value < 0.05; **P value < 0.01; ***P value < 0.001; ****P value < 0.0001.

Oncopression database was utilized to validate the expression values of respective top 10 hub genes in the PPI network. As shown in Figure 7, eight of the top 10 downregulated hub genes (IL6, EGFR, CXCL8, CAT, IGF1, IL1B, PTGS2, and FOS) had significantly lower expression levels in HB tissue samples compared to normal liver tissue samples, while nine of the top 10 upregulated hub genes (CDK1, CCNB1, KIF11, NCAPG, TOP2A, AURKA, TP53, ASPM, and TPX2) had markedly higher expression levels in HB tissues in comparison to normal liver tissues.

### Hub Genes Verification via qRT-PCR

**FIGURE 8:**
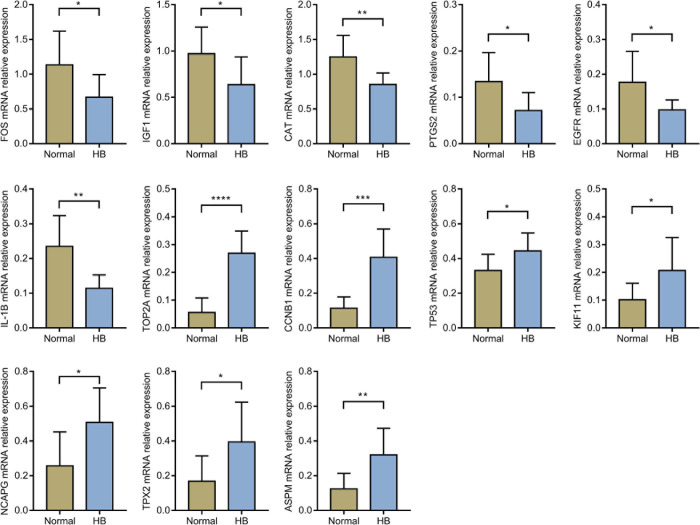
The comparison of hub genes expression levels in HB and normal liver tissues in eight children with HB from our hospital. HB, hepatoblastoma. *P value < 0.05; **P value < 0.01; ***P value < 0.001; ****P value < 0.0001.

**FIGURE 9:**
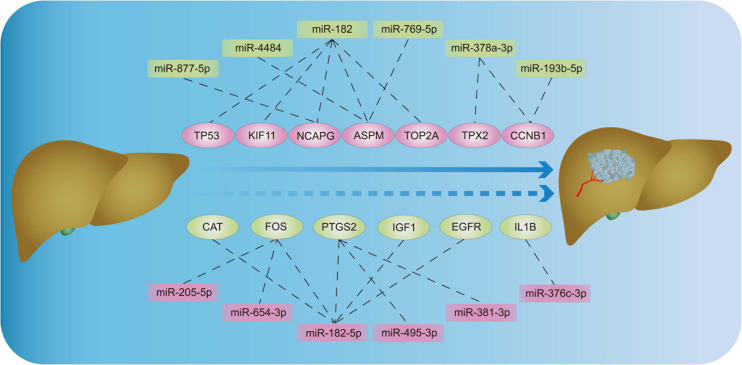
The potential miRNA–mRNA interaction network involved in HB. HB: hepatoblastoma.

Through literature search, we identified AURKA (Zhang Y. et al., 2018; Tan et al., 2020) and CDK1 (Tian et al., 2021) as previously reported oncogenic genes in HB. In contrast, the role of the other 15 hub genes in HB has not been reported to date or remains controversial. Based on HB and matched normal liver tissue samples in eight children with HB, the mRNA expression levels of these 15 hub genes were validated by qRT-PCR. The expression levels of EGFR, CAT, IGF1, IL1B, PTGS2, and FOS were significantly lower for HB tissues when compared with normal liver tissues. On the other hand, the expression values of CCNB1, KIF11, NCAPG, TOP2A, TP53, ASPM, and TPX2 in HB tissues were significantly higher than those in the normal liver tissues (Figure 8). Lastly, according to the predicted miRNA–mRNA pairs and the final verification results, we constructed the potential miRNA–mRNA interaction network involved in HB (Figure 9).

## Discussion

The extreme rarity of HB has hindered our understanding of its underlying molecular mechanisms, and the majority of potential hub genes, DE-miRNAs and TFs in this study were reported for the first time in HB pathogenesis. Therefore, our work may serve as an important resource for future studies to unveil the underlying mechanisms of these key biomarkers and/or therapeutic targets involved in HB.

The dysregulation of miRNA–mRNA interaction network in liver is associated with various liver diseases, such as liver regeneration (Wang et al., 2019) and hepatocellular carcinoma (Zhang and Du, 2017; Lou et al., 2019). In the context of HB, previous studies have identified multiple miRNAs as promising therapeutic targets (von Frowein et al., 2018; Cui et al., 2019a; Liu et al., 2019). In order to provide an overall picture of miRNA–mRNA interaction network in HB pathogenesis, we performed a comprehensive bioinformatic analysis on the basis of two independent GEO datasets. Among the TFs predicted for DE-miRNAs in this study, CDKN1A was reported to regulate the G1/S transition and affect replication and damage repair of DNA during mitosis (Tokumoto et al., 2003). In addition, AP2 negatively controls the growth of HepG2 HB cells through CDKN1A activation (Zeng et al., 1997). USP7, another predicted TF in this study, was reported to promote proliferation, migration and invasion of HB cell lines through activation of PI3K/AKT signaling (Ye et al., 2021). Apart from CDKN1A and USP7, HNF4A was reported to be essential for Smad2/3 binding regions in HepG2 HB cells, thus affecting transcription regulated by TGF-β (Mizutani et al., 2011). Future studies are needed to validate the molecular mechanisms of KLF4, PKM, and other TFs in the pathogenesis of HB development.

In the process of functional annotation enrichment analyses, GO-MF analysis on the basis of downregulated candidate genes identified enriched terms relating to oxidative stress injury such as oxidoreductase activity (Figure 4E). Similar to our findings, previous study also reported that oxidative stress injury plays an essential role in HB development (Tang et al., 2018). Our KEGG analysis of the upregulated candidate genes revealed that the PI3K/AKT pathway is another crucial pathway involved in HB (Figure 5G). In human embryonal tumors, the PI3K/AKT pathway is perhaps the most frequently reported pathway with hyperactivation (Vivanco and Sawyers, 2002; Zhang et al., 2004; Hartmann et al., 2006). In HB cells, it was reported that additive anti-tumor effects can be achieved after combination chemotherapy with PI3K inhibitors (Hartmann et al., 2009).

The cell cycle is composed of the interphase and the mitotic phase. The interphase, including G1, S, and G2 phases, is characterized by the synthesis of DNA and proteins (Norbury and Nurse, 1992). Uncontrolled cell cycle is recognized as a hallmark of tumor and, therefore, constitutes a major therapeutic target for the development of anti-tumor agents. Our KEGG enrichment analysis of the upregulated candidate genes identified cell cycle as the most significantly enriched pathway in HB (Figure 5G). For BP within the GO analysis, we found that the upregulated candidate genes played vital roles in multiple cell cycle events, including mitotic nuclear division and chromosome segregation (Figure 5A). Cellular defects that affect chromosome separation may increase aneuploidy, which in turn accelerate tumor progression (Pines, 2006). Moreover, other key events that interfere with the cell cycle were also observed in CC and MF within the GO analysis (Figures 5C,E).

In addition to the functional annotation enrichment analyses, almost all the upregulated hub genes obtained in this study, including CCNB1, KIF11, NCAPG, TOP2A, ASPM, and TPX2, have been previously reported to be implicated in regulating cell cycle progression. Indeed, the upregulated cell cycle-related proteins can accelerate cellular proliferation in human tumors (Malumbres and Barbacid, 2009). Moreover, the progression through distinct cell cycle phases is monitored by checkpoints that allow or prohibit the progression from one stage to another. Abnormal cell cycle check point hampers the detection and repair of genetic damage, leading to uncontrolled cell division and tumorigenesis. The majority of tumor cells exhibit cell cycle checkpoint defects, among which G1/S phase checkpoint defect is the most typical one (Zhao et al., 2012). CCNB1 is a regulatory protein involved in the G2/M cell cycle transition, and CCNB1 overexpression promotes chromatin bridging by suppressing separase activation (Nam and van-Deursen, 2014). In addition, the proliferation of human HB cell line HepG2 is suppressed by lycorine in a dose-dependent manner through downregulating cyclin A, CCNB1 and CDK1 (Liu et al., 2018). Centrosome linker refers to the protein that concatenates centrosomes during interphase. In the complex of mitotic spindle assembly, the dissolution of the centrosome linker is driven by KIF11 (Hata et al., 2019), a motor protein capable of hydrolyzing ATP. Besides, TPX2 can also regulate mitotic spindle assembly through kinetochore dependent microtubule nucleation and AURKA localization (Moss et al., 2009). NCAPG serves as the regulatory subunit of the condensin complex, which is essential for the conversion between interphase chromatin and mitotic chromosome in the presence of topoisomerases (Kimura et al., 2001). TOP2A, one type of nuclear enzyme, is critical for removing topological barriers left on DNA during mitosis (Linka et al., 2007). ASPM is implicated in the regulation of mitotic spindle and the orchestration of mitotic processes. Also, the microtubule dynamics at spindle poles are modulated by ASPM with the help of the katanin complex (Jiang et al., 2017).

TP53 is famous for its tumor suppressive role in a variety of human tumors (Aubrey et al., 2018). Interestingly, our results demonstrated that TP53 plays an oncogenic role in HB development. Actually, there are two types of TP53, namely, mutant TP53 (mutp53) and wild type TP53 (wtp53). Missense mutation is the predominant form of mutp53 and expresses full-length mutp53 protein (Olivier et al., 2004). Mutp53 cannot activate the target genes of wtp53 or induce MDM2 expression, leading to the accumulation of mutp53 proteins in HB (Yue et al., 2017). Loss of heterozygosity (LOH) represents the phenomenon that mutp53 may inhibit the function of wtp53 and provide tumor cells with oncogenic functions (Baker et al., 1990). In addition, gain of function (GOF) is defined as the effect of mutp53 on promoting proliferation, metastasis, and anti-apoptosis of tumor cells. A greater number of metastatic tumors was observed for mice expressing mutp53 when compared with *TP53*^–/–^ mice (Lang et al., 2004; Olive et al., 2004). The expression of mutp53 has been associated with chemoresistance in certain tumors due to GOF and the loss of wtp53 pro-apoptotic function (Yue et al., 2017). Patients with Li-Fraumeni syndrome and mutp53 were reported to have earlier development of tumors compared with those with Li-Fraumeni syndrome and TP53 deletion (Bougeard et al., 2008). Mutp53 can promote oncogenic cellular changes and alter cellular transcriptional profile. Therefore, to the best of our knowledge, the more likely scenario in this study was that most of over-expressed TP53 proteins in HB may belong to mutp53, thereby exerting oncogenic functions.

It is common that one dataset consists of a combination of paired and independent observations, and the terminology for this described scenario is “partially paired data” (Guo and Yuan, 2017). It should be noted that there are partially paired data in both datasets used for the present study. However, we did not take the inherent pairing structure into consideration in the DE analyses, which can lead to suboptimal results (Kuan and Huang, 2013). When analyzing partially paired data, the optimal pooled *t*-test, the test based on the modified maximum likelihood estimator, or the paired *t*-test, is to be recommended under different conditions in order to improve the statistical power (Guo and Yuan, 2017). Therefore, ignoring the matching for partially matched samples is one of the limitations of this study.

In conclusion, our results identify a variety of DE-miRNAs, TFs, and hub genes as potential regulators in the pathogenesis of HB. In addition, the miRNA–mRNA interaction network, PPI modules, and pathways may suggest putative diagnostic biomarkers or therapeutic targets for future HB theranostics.

## Data Availability Statement

The datasets presented in this study can be found in online repositories. The names of the repository/repositories and accession number(s) can be found in the article/Supplementary Material.

## Ethics Statement

The studies involving human participants were reviewed and approved by the medical research ethics committee of Shanghai Children’s Hospital, Shanghai Jiao Tong University. Written informed consent to participate in this study was provided by the participants’ legal guardian/next of kin.

## Author Contributions

ZL, JL, and LT study design. TC, LT, JC, XZ, JZ, and TG analysis and visualization of data. TC, QS, and LZ manuscript writing. TC, LT, and JL manuscript revision. All authors contributed to the article and approved the submitted version.

## Conflict of Interest

The authors declare that the research was conducted in the absence of any commercial or financial relationships that could be construed as a potential conflict of interest.

## Publisher’s Note

All claims expressed in this article are solely those of the authors and do not necessarily represent those of their affiliated organizations, or those of the publisher, the editors and the reviewers. Any product that may be evaluated in this article, or claim that may be made by its manufacturer, is not guaranteed or endorsed by the publisher.

## Footnotes

1
https://www.ncbi.nlm.nih.gov/geo/

2
http://oncopression.com/

## Supplementary Material

Supplementary Figure 1 Data preprocessing of fetal-type tumor and normal liver samples in the GSE153089 dataset. Data of fetal-type tumor and normal liver samples **(A)** prior to and **(B)** after normalization. PCA of fetal-type tumor and normal liver samples **(C)** prior to and **(D)** after excluding the outlier sample. PCA, principal component analysis.

Supplementary Figure 2 Data preprocessing of embryonal-type tumor and normal liver samples in the GSE153089 dataset. Data of embryonal-type tumor and normal liver samples **(A)** before and **(B)** after normalization. PCA of embryonal-type tumor and normal liver samples **(C)** prior to and **(D)** after excluding the outlier sample. PCA, principal component analysis.

Supplementary Figure 3 Data preprocessing of HB and normal liver samples in the GSE131329 dataset. Data of HB and normal liver samples **(A)** before and **(B)** after normalization. PCA of HB and normal liver samples **(C)** prior to and **(D)** after excluding the outlier sample. HB: hepatoblastoma; PCA: principal component analysis.
