# Supplementary figures and images for: A Comprehensive Genomic Analysis Constructs miRNA–mRNA Interaction Network in Hepatoblastoma
Source: Front Cell Dev Biol. 2021 Aug 6;9:655703. doi: 10.3389/fcell.2021.655703 (PMC8377242; doi:10.3389/fcell.2021.655703)

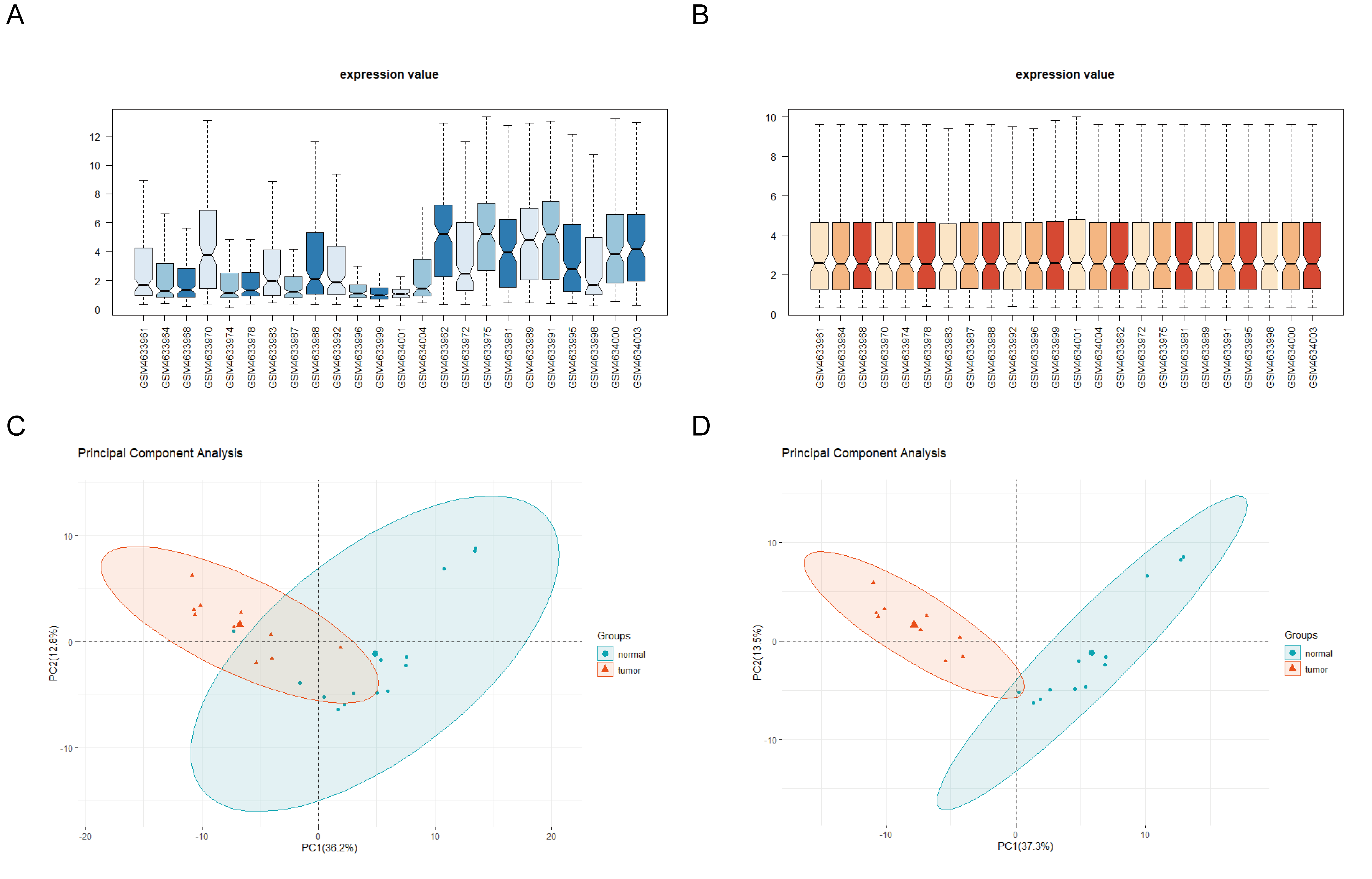

Supplement: Supplementary Figure 1 — Data preprocessing of fetal-type tumor and normal liver samples in the GSE153089 dataset. Data of fetal-type tumor and normal liver samples (A) prior to and (B) after normalization. PCA of fetal-type tumor and normal liver samples (C) prior to and (D) after excluding the outlier sample. PCA, principal component analysis. [file Image_1.TIF]

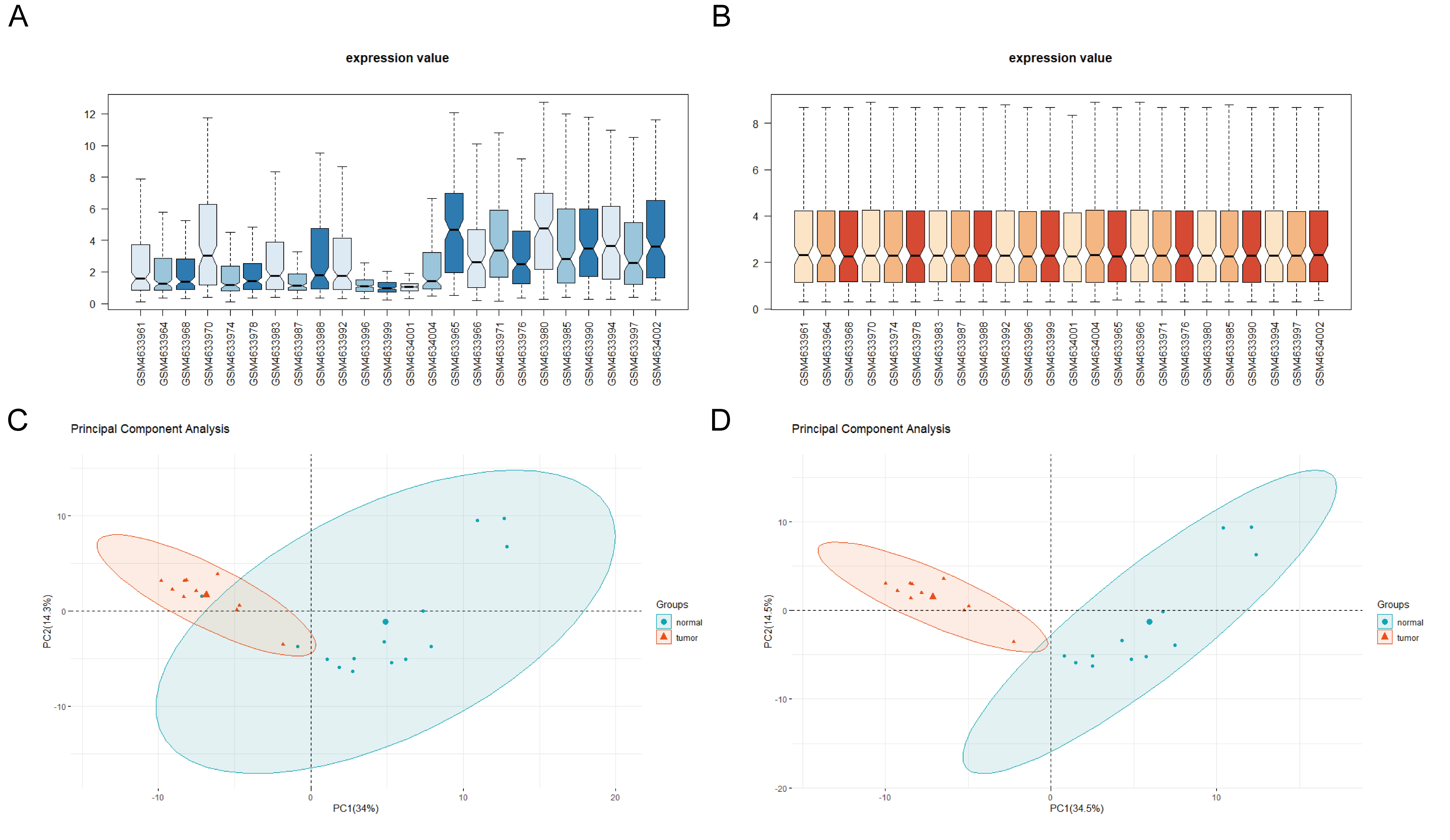

Supplement: Supplementary Figure 2 — Data preprocessing of embryonal-type tumor and normal liver samples in the GSE153089 dataset. Data of embryonal-type tumor and normal liver samples (A) before and (B) after normalization. PCA of embryonal-type tumor and normal liver samples (C) prior to and (D) after excluding the outlier sample. PCA, principal component analysis. [file Image_2.TIF]

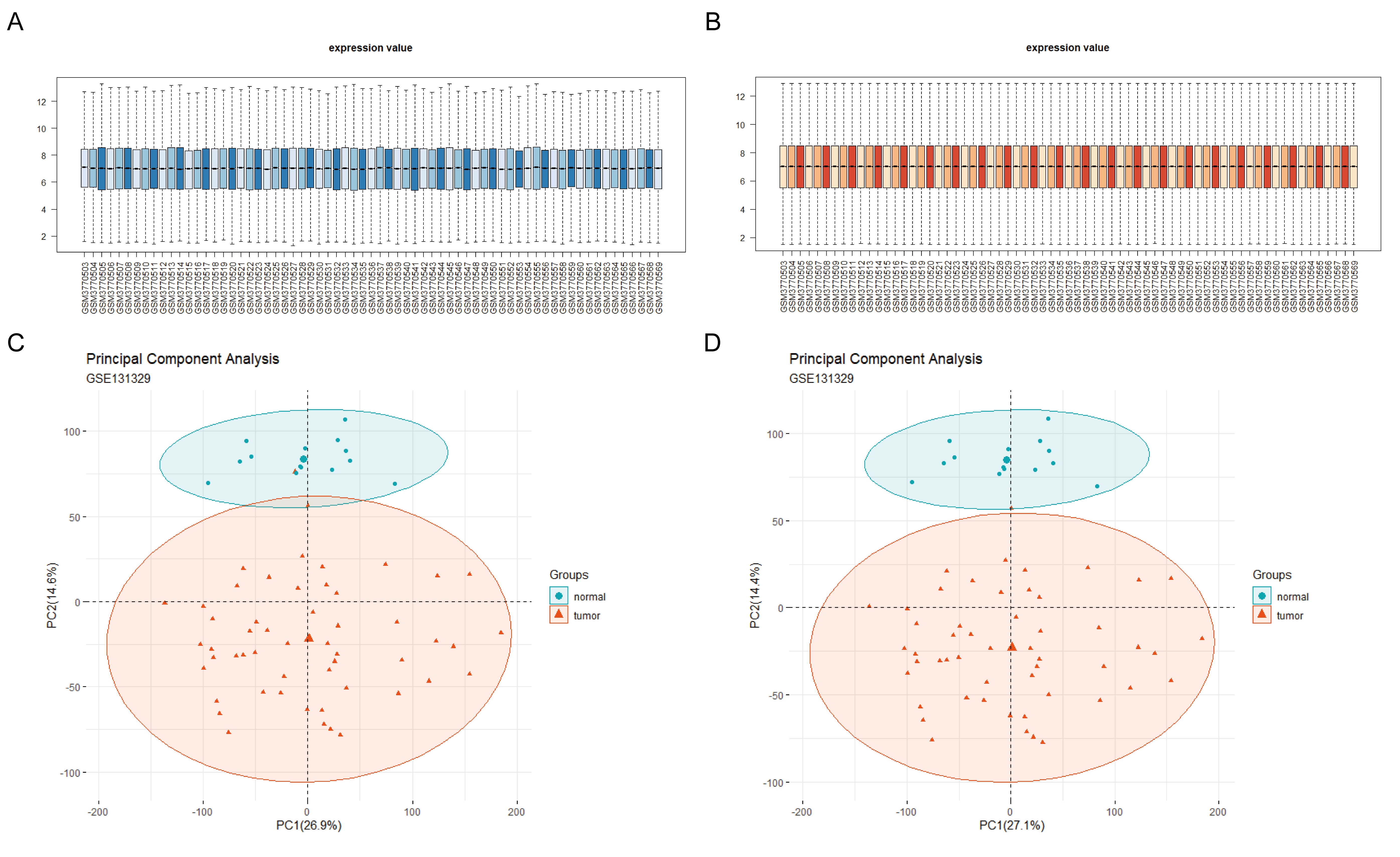

Supplement: Supplementary Figure 3 — Data preprocessing of HB and normal liver samples in the GSE131329 dataset. Data of HB and normal liver samples (A) before and (B) after normalization. PCA of HB and normal liver samples (C) prior to and (D) after excluding the outlier sample. HB: hepatoblastoma; PCA: principal component analysis. [file Image_3.TIF]
