# Supplementary material for: A Comprehensive Genomic Analysis Constructs miRNA–mRNA Interaction Network in Hepatoblastoma
Source: Front Cell Dev Biol. 2021 Aug 6;9:655703. doi: 10.3389/fcell.2021.655703 (PMC8377242; doi:10.3389/fcell.2021.655703)
Supplement: Supplementary file 4 [file Table_1.DOCX]

**Table S1. General information of the two datasets used for the present study.**

| **ID** | **Platform** | **Normal** | **Tumor** | **Year** | **Country** |
| --- | --- | --- | --- | --- | --- |
| GEO153089 | Affymetrix Multispecies miRNA-4 Array | 14 | 30 | 2020 | Japan |
| GSE131329 | Affymetrix mRNA microarray | 14 | 53 | 2019 | USA |

GEO, Gene Expression Omnibus.
