# Supplementary material for: A Comprehensive Genomic Analysis Constructs miRNA–mRNA Interaction Network in Hepatoblastoma
Source: Front Cell Dev Biol. 2021 Aug 6;9:655703. doi: 10.3389/fcell.2021.655703 (PMC8377242; doi:10.3389/fcell.2021.655703)
Supplement: Supplementary file 5 [file Table_2.DOCX]

**Table S2. Detailed information of samples in the GSE153089 dataset used for the present study.**

| **Group** | **Case Number** | **Sample ID** |
| --- | --- | --- |
| Normal surrounding liver | 1 | 22N |
|  | 2 | 24N |
|  | 3 | H11N |
|  | 4 | 7N |
|  | 5 | H12N |
|  | 6 | H16N |
|  | 7 | 25N |
|  | 8 | 8N |
|  | 9 | H09N |
|  | 10 | 2N |
|  | 11 | H10N |
|  | 12 | 6N |
|  | 14 | 3N |
|  | 15 | 9N |
| Fetal tumor | 1 | 22F |
|  | 2 | 24F |
|  | 3 | H11F |
|  | 5 | H12F |
|  | 6 | H16F |
|  | 7 | 25F-1 |
|  | 8 | 8F |
|  | 9 | H09F |
|  | 11 | H10F |
|  | 12 | 6F |
| Embryonal tumor | 2 | 24E |
|  | 4 | 7E |
|  | 5 | H12E |
|  | 6 | H16E |
|  | 8 | 8E |
|  | 9 | H09E |
|  | 11 | H10E |
|  | 12 | 6E |
|  | 14 | 3E |
|  | 15 | 9E |
