# Supplementary material for: A Comprehensive Genomic Analysis Constructs miRNA–mRNA Interaction Network in Hepatoblastoma
Source: Front Cell Dev Biol. 2021 Aug 6;9:655703. doi: 10.3389/fcell.2021.655703 (PMC8377242; doi:10.3389/fcell.2021.655703)
Supplement: Supplementary file 6 [file Table_3.DOCX]

**Table S3. The sequence of primers used for qRT-PCR.**

| **Gene name** | **Forward primer sequence** | **Reverse primer sequence** |
| --- | --- | --- |
| TP53 | TTCCTGAAAACAACGTTCTGTC | AACCATTGTTCAATATCGTCCG |
| TOP2A | AAGATTCATTGAAGACGCTTCG | GCTGTAAAATGCCATTTCTTGC |
| NCAPG | ATTGCTTTGTATTGGTGTGCCCTTTG | ACAACTGGAATGCTCTGGATGTAACTC |
| KIF11 | CATACTCTAGTCGTTCCCACTC | CAACCAAGTTCAACTTTCCGAT |
| CCNB1 | GACTTTGCTTTTGTGACTGACA | CCCAGACCAAAGTTTAAAGCTC |
| ASPM | AAGAATGTCTGAGCCAGCGAAATAGG | CCTCCAAGAATAGCCTCTCCATAATGC |
| PTGS2 | TGTCAAAACCGAGGTGTATGTA | AACGTTCCAAAATCCCTTGAAG |
| IL6 | CACTGGTCTTTTGGAGTTTGAG | GGACTTTTGTACTCATCTGCAC |
| IL1B | GCCAGTGAAATGATGGCTTATT | AGGAGCACTTCATCTGTTTAGG |
| IGF1 | AAAAATCAGCAGTCTTCCAACC | CCTGTGGGCTTGTTGAAATAAA |
| FOS | CTTCCCAGAAGAGATGTCTGTG | TGGGAACAGGAAGTCATCAAAG |
| EGFR | ACCCATATGTACCATCGATGTC | GAATTCGATGATCAACTCACGG |
| CXCL8 | GCCAGTGAAATGATGGCTTATT | AGGAGCACTTCATCTGTTTAGG |
| CAT | GAGCACAGCATCCAATATTCTG | CTCATTCAGCACGTTCACATAG |
| GAPDH | CGACCACTTTGTCAAGCTCA | AGGGGTCTACATGGCAACTG |

qRT-PCR, quantitative real-time polymerase chain reaction.
