# Supplementary material for: A Comprehensive Genomic Analysis Constructs miRNA–mRNA Interaction Network in Hepatoblastoma
Source: Front Cell Dev Biol. 2021 Aug 6;9:655703. doi: 10.3389/fcell.2021.655703 (PMC8377242; doi:10.3389/fcell.2021.655703)
Supplement: Supplementary file 7 [file Table_4.DOCX]

**Table S4. Upregulated hub miRNAs through the intersection of two clusters of DE-miRNAs from the GSE153089 dataset.**

| **DE-miRNAs** | **logFC** | **AveExpr** | ***t*** | ***P*.Value** | **adj.*P*.Val** | **B** |
| --- | --- | --- | --- | --- | --- | --- |
| hsa-miR-487b-3p | 5.362130318 | 6.477876039 | 6.547094668 | 1.24E-06 | 9.26E-06 | 5.28556751 |
| hsa-miR-432-5p | 4.999874613 | 7.096197089 | 7.701105401 | 9.49E-08 | 1.06E-06 | 7.865329979 |
| hsa-miR-431-5p | 4.320508708 | 3.780044407 | 8.45520786 | 1.95E-08 | 3.06E-07 | 9.454707559 |
| hsa-miR-493-3p | 3.944528908 | 3.463758503 | 9.582611101 | 2.10E-09 | 7.99E-08 | 11.68650895 |
| hsa-miR-379-5p | 3.849086062 | 6.120611536 | 5.163456092 | 3.32E-05 | 0.00013032 | 1.994506966 |
| hsa-miR-376a-3p | 3.837815118 | 3.216293698 | 13.30705213 | 3.80E-12 | 5.97E-10 | 17.9454979 |
| hsa-miR-127-3p | 3.729050649 | 8.648216647 | 5.307694336 | 2.34E-05 | 0.000101912 | 2.344809705 |
| hsa-miR-487a-3p | 3.592332038 | 4.360249197 | 8.027936531 | 4.73E-08 | 6.19E-07 | 8.563735631 |
| hsa-miR-205-5p | 3.468711971 | 3.19474726 | 3.319233596 | 0.003050514 | 0.007724688 | -2.451756027 |
| hsa-miR-376c-3p | 3.278021109 | 4.682186791 | 6.525757963 | 1.30E-06 | 9.29E-06 | 5.236284182 |
| hsa-miR-382-5p | 3.262090498 | 6.693777872 | 6.331259441 | 2.04E-06 | 1.39E-05 | 4.784587828 |
| hsa-miR-409-3p | 2.735722002 | 7.223176492 | 8.741540391 | 1.09E-08 | 2.44E-07 | 10.03779777 |
| hsa-miR-337-5p | 2.659292603 | 4.960044879 | 5.322839739 | 2.25E-05 | 0.000101912 | 2.381525549 |
| hsa-miR-654-3p | 2.589950948 | 4.412698885 | 6.280940072 | 2.30E-06 | 1.50E-05 | 4.667027028 |
| hsa-miR-134-5p | 2.471789986 | 6.272808721 | 4.442589082 | 0.000195354 | 0.00071327 | 0.234724838 |
| hsa-miR-409-5p | 2.432966821 | 3.583733466 | 6.607707475 | 1.08E-06 | 8.46E-06 | 5.425274601 |
| hsa-miR-485-3p | 2.356645172 | 3.740032124 | 5.185507772 | 3.15E-05 | 0.000129459 | 2.048132353 |
| hsa-miR-433-3p | 2.277988316 | 4.551948696 | 4.460027363 | 0.000187121 | 0.000699478 | 0.277293656 |
| hsa-miR-493-5p | 2.171057512 | 2.910135346 | 6.041255577 | 4.03E-06 | 2.53E-05 | 4.103291913 |
| hsa-miR-411-5p | 2.10469122 | 3.090821287 | 5.846885269 | 6.38E-06 | 3.58E-05 | 3.641841251 |
| hsa-miR-381-3p | 2.00270544 | 4.292119709 | 4.294137662 | 0.00028185 | 0.000961968 | -0.127225617 |
| hsa-miR-182-5p | 1.546819334 | 3.463058154 | 2.556173007 | 0.017826002 | 0.035344495 | -4.123307493 |
| hsa-miR-758-3p | 1.517992174 | 1.928562453 | 4.303988443 | 0.000275078 | 0.000959717 | -0.103236772 |
| hsa-miR-154-5p | 1.460403715 | 4.422491969 | 3.725699954 | 0.001140664 | 0.003511455 | -1.498486778 |
| hsa-miR-487a-5p | 1.449202629 | 2.83092191 | 4.063099379 | 0.000498342 | 0.001629993 | -0.688156826 |
| hsa-miR-485-5p | 1.389096671 | 4.086145597 | 3.036154135 | 0.005963764 | 0.013974789 | -3.093721419 |
| hsa-miR-412-5p | 1.369690945 | 1.785411943 | 3.108598038 | 0.00503083 | 0.011967277 | -2.931518421 |
| hsa-miR-323a-3p | 1.290215774 | 1.719331066 | 3.787687534 | 0.000980246 | 0.003077973 | -1.350646611 |
| hsa-miR-654-5p | 1.284420194 | 3.282848403 | 2.82170813 | 0.009800201 | 0.021369884 | -3.56415157 |
| hsa-miR-495-3p | 1.240060075 | 3.19246953 | 2.69106003 | 0.013187912 | 0.02797976 | -3.842781493 |
| hsa-miR-377-5p | 1.220881146 | 2.89037722 | 4.146570765 | 0.00040567 | 0.001355109 | -0.48592408 |
| hsa-miR-410-3p | 1.00678227 | 1.949478694 | 3.17751334 | 0.004275006 | 0.010325784 | -2.775827775 |
| hsa-miR-431-3p | 1.002659695 | 2.617662273 | 2.259558951 | 0.033854929 | 0.063276474 | -4.711086631 |
