# Supplementary material for: A Comprehensive Genomic Analysis Constructs miRNA–mRNA Interaction Network in Hepatoblastoma
Source: Front Cell Dev Biol. 2021 Aug 6;9:655703. doi: 10.3389/fcell.2021.655703 (PMC8377242; doi:10.3389/fcell.2021.655703)
Supplement: Supplementary file 8 [file Table_5.DOCX]

**Table S5. Downregulated hub miRNAs through the intersection of two clusters of DE-miRNAs from the GSE153089 dataset.**

| **DE‐miRNA** | **logFC** | **AveExpr** | ***t*** | ***P*.Value** | **adj.*P*.Val** | **B** |
| --- | --- | --- | --- | --- | --- | --- |
| hsa-miR-193b-5p | -3.77390625 | 5.847227183 | -10.86363739 | 2.02E-10 | 1.59E-08 | 14.0212691 |
| hsa-miR-5589-5p | -3.469618607 | 2.925689248 | -6.771001005 | 7.43E-07 | 6.14E-06 | 5.799436147 |
| hsa-miR-4484 | -3.41144042 | 7.990993952 | -5.721425416 | 8.61E-06 | 4.50E-05 | 3.342117201 |
| hsa-miR-378a-3p | -3.03736869 | 9.499747496 | -4.32912362 | 0.000258525 | 0.000922465 | -0.042005956 |
| hsa-miR-769-5p | -2.201149554 | 3.518778581 | -5.744233249 | 8.16E-06 | 4.42E-05 | 3.396708584 |
| hsa-miR-3916 | -2.163894952 | 3.105870418 | -7.310827741 | 2.22E-07 | 2.18E-06 | 7.012362792 |
| hsa-miR-877-5p | -2.077954144 | 5.025721372 | -6.985873609 | 4.57E-07 | 3.99E-06 | 6.286725396 |
| hsa-miR-6823-3p | -1.744823082 | 1.668133031 | -3.702039764 | 0.001208492 | 0.003648717 | -1.5547707 |
| hsa-mir-127 | -1.472419395 | 2.40057894 | -3.644514921 | 0.001390407 | 0.004042479 | -1.691256091 |
| hsa-mir-487a | -1.466270117 | 3.83980702 | -2.658577281 | 0.014187665 | 0.028928095 | -3.911025953 |
| hsa-mir-182 | -1.439202767 | 2.137432445 | -5.441296479 | 1.69E-05 | 8.04E-05 | 2.668197462 |
| hsa-miR-6511a-3p | -1.346103395 | 2.596228505 | -5.17654914 | 3.22E-05 | 0.000129459 | 2.026349538 |
