# Supplementary material for: A Comprehensive Genomic Analysis Constructs miRNA–mRNA Interaction Network in Hepatoblastoma
Source: Front Cell Dev Biol. 2021 Aug 6;9:655703. doi: 10.3389/fcell.2021.655703 (PMC8377242; doi:10.3389/fcell.2021.655703)
Supplement: Supplementary file 9 [file Table_6.DOCX]

**Table S6. The TFs predicted for the upregulated DE-miRNAs.**

| **TF** | **Counts** | **Percent** | **Fold** | **P-value** | **Bonferroni** | **FDR** |
| --- | --- | --- | --- | --- | --- | --- |
| HNF4A | 19 | 0.07 | 4.7704 | 1.73E-10 | 1.96E-08 | 1.96E-08 |
| GTF2I | 20 | 0.05 | 3.3051 | 2.29E-08 | 2.59E-06 | 1.30E-06 |
| CEBPB | 25 | 0.03 | 1.9832 | 6.76E-07 | 7.64E-05 | 2.55E-05 |
| CREB1 | 25 | 0.03 | 1.9011 | 0.00000179 | 2.02E-04 | 5.05E-05 |
| MAZ | 24 | 0.03 | 1.9318 | 0.00000479 | 5.42E-04 | 1.08E-04 |
| NR3B3 | 2 | 1 | 71.5556 | 1.08E-03 | 0.122 | 0.0174 |
| SHP | 2 | 1 | 71.5556 | 1.08E-03 | 0.122 | 0.0174 |
| KLF4 | 6 | 0.05 | 3.903 | 4.02E-03 | 0.4541 | 0.0568 |
| PKM | 1 | 0.5 | 35.7778 | 0.0408 | 1 | 0.4697 |
| EED | 2 | 0.09 | 6.2222 | 0.0452 | 1 | 0.4697 |

DE-miRNAs, differentially expressed miRNAs; TF, transcription factor.
