# Supplementary material for: A Comprehensive Genomic Analysis Constructs miRNA–mRNA Interaction Network in Hepatoblastoma
Source: Front Cell Dev Biol. 2021 Aug 6;9:655703. doi: 10.3389/fcell.2021.655703 (PMC8377242; doi:10.3389/fcell.2021.655703)
Supplement: Supplementary file 10 [file Table_7.DOCX]

**Table S7. The TFs predicted for the downregulated DE-miRNAs.**

| **TF** | **Counts** | **Percent** | **Fold** | **P-value** | **Bonferroni** | **FDR** |
| --- | --- | --- | --- | --- | --- | --- |
| NR3B3 | 1 | 0.5 | 87.8182 | 0.0169 | 1 | 0.9836 |
| SHP | 1 | 0.5 | 87.8182 | 0.0169 | 1 | 0.9836 |
| CDKN1A | 1 | 0.33 | 58.5455 | 0.0225 | 1 | 0.9836 |
| KLF3 | 1 | 0.25 | 43.9091 | 0.028 | 1 | 0.9836 |
| USP7 | 1 | 0.25 | 43.9091 | 0.028 | 1 | 0.9836 |
| HSF2 | 1 | 0.2 | 35.1273 | 0.0335 | 1 | 0.9836 |

DE-miRNAs, differentially expressed miRNAs; TF, transcription factor.
