# Supplementary material for: A Comprehensive Genomic Analysis Constructs miRNA–mRNA Interaction Network in Hepatoblastoma
Source: Front Cell Dev Biol. 2021 Aug 6;9:655703. doi: 10.3389/fcell.2021.655703 (PMC8377242; doi:10.3389/fcell.2021.655703)
Supplement: Supplementary file 11 [file Table_8.DOCX]

**Table S8. Upregulated DE-mRNAs between HB and normal liver samples from the GSE131329 dataset.**

| **Upregulated DE‐mRNA** | **logFC** | **AveExpr** | **t** | **P.Value** | **adj.P.Val** | **B** |
| --- | --- | --- | --- | --- | --- | --- |
| GXYLT2 | 3.334517819 | 8.364690495 | 17.8484106 | 2.78E-27 | 3.32E-23 | 51.49894941 |
| DLK1 | 4.120675061 | 11.54621226 | 17.45390017 | 9.65E-27 | 6.05E-23 | 50.29341104 |
| NPNT | 3.282197308 | 9.598291702 | 16.03675757 | 9.85E-25 | 2.32E-21 | 45.80093392 |
| NKD1 | 3.145090499 | 10.52862601 | 15.89662858 | 1.58E-24 | 3.30E-21 | 45.34263469 |
| TMEM98 | 1.846653982 | 9.376720205 | 15.71996329 | 2.87E-24 | 5.39E-21 | 44.76116927 |
| TNFRSF19 | 2.549917369 | 9.039290294 | 15.18077239 | 1.82E-23 | 3.11E-20 | 42.96101073 |
| EPCAM | 3.913777829 | 9.037220208 | 14.71726318 | 9.14E-23 | 1.23E-19 | 41.3825861 |
| LGR5 | 3.943358655 | 9.996774512 | 14.59298078 | 1.42E-22 | 1.78E-19 | 40.954467 |
| NOTUM | 3.420113888 | 10.86205998 | 14.24553297 | 4.87E-22 | 5.39E-19 | 39.7466008 |
| ROBO1 | 2.131387277 | 10.26094412 | 13.98402381 | 1.25E-21 | 1.23E-18 | 38.82679204 |
| GPC3 | 4.203513524 | 11.92787331 | 13.81144344 | 2.33E-21 | 1.99E-18 | 38.21474171 |
| BMP4 | 2.781595611 | 8.853555306 | 13.67471969 | 3.83E-21 | 3.14E-18 | 37.72701883 |
| IGF2BP1 | 3.278136653 | 9.435421454 | 13.63840078 | 4.37E-21 | 3.43E-18 | 37.59704041 |
| PEG10 | 3.606445313 | 11.57976408 | 13.4340293 | 9.26E-21 | 6.97E-18 | 36.86234393 |
| BAMBI | 2.032911916 | 10.0422033 | 13.28030996 | 1.63E-20 | 1.10E-17 | 36.3060639 |
| ITGA2 | 3.000647405 | 8.564609149 | 13.21249818 | 2.10E-20 | 1.32E-17 | 36.05966695 |
| SERPINE2 | 2.662207835 | 9.340766133 | 12.72044427 | 1.32E-19 | 6.55E-17 | 34.25356307 |
| TYMS | 2.600472846 | 9.10542652 | 12.6322531 | 1.84E-19 | 8.26E-17 | 33.92649794 |
| DKK1 | 4.64774927 | 9.958413498 | 12.49099962 | 3.15E-19 | 1.38E-16 | 33.40054297 |
| OXCT1 | 1.771004551 | 6.955956748 | 12.43319633 | 3.92E-19 | 1.67E-16 | 33.18457028 |
| TRIB2 | 1.598725714 | 9.389235932 | 12.42424638 | 4.06E-19 | 1.67E-16 | 33.1510918 |
| IGF2BP2 | 1.946770317 | 9.200256122 | 12.3475407 | 5.44E-19 | 2.18E-16 | 32.86374247 |
| HSDL1 | 1.410013246 | 7.973313664 | 12.11538994 | 1.32E-18 | 4.89E-16 | 31.98950645 |
| MAGED1 | 1.502744302 | 11.4439792 | 12.04874541 | 1.71E-18 | 6.19E-16 | 31.73727739 |
| GREB1 | 2.487918261 | 8.69094127 | 11.91865855 | 2.83E-18 | 9.64E-16 | 31.24333884 |
| LEF1 | 3.017950221 | 8.792682304 | 11.91505637 | 2.87E-18 | 9.64E-16 | 31.22963143 |
| CEP68 | 1.068679968 | 7.992141802 | 11.83482106 | 3.91E-18 | 1.25E-15 | 30.92389614 |
| APCDD1 | 3.513632062 | 10.56039236 | 11.79790704 | 4.52E-18 | 1.38E-15 | 30.78296959 |
| SLC7A6 | 1.655143258 | 8.302813495 | 11.52820043 | 1.29E-17 | 3.55E-15 | 29.74828275 |
| GNA12 | 1.348519597 | 9.283776244 | 11.52677278 | 1.30E-17 | 3.55E-15 | 29.7427825 |
| MACROH2A2 | 1.799152743 | 8.726787923 | 11.45722063 | 1.71E-17 | 4.53E-15 | 29.47452871 |
| FAM3B | 3.240433665 | 8.834542419 | 11.40075685 | 2.13E-17 | 5.49E-15 | 29.25633448 |
| FRRS1L | 3.055649624 | 9.362113611 | 11.15620843 | 5.59E-17 | 1.35E-14 | 28.30703654 |
| GNG4 | 3.050858364 | 8.867541969 | 11.10703772 | 6.79E-17 | 1.60E-14 | 28.11533644 |
| RACGAP1 | 1.738577693 | 8.013111326 | 11.06778293 | 7.94E-17 | 1.78E-14 | 27.96209959 |
| ITGA6 | 2.350675736 | 9.396464863 | 11.03629286 | 8.99E-17 | 1.95E-14 | 27.83904868 |
| PLCG1 | 1.36143934 | 9.237701437 | 10.97871552 | 1.13E-16 | 2.39E-14 | 27.61377368 |
| NRXN3 | 2.421352262 | 7.789558247 | 10.91763371 | 1.44E-16 | 2.92E-14 | 27.37438737 |
| RNF43 | 2.223383012 | 8.829624388 | 10.81668171 | 2.16E-16 | 4.26E-14 | 26.97785441 |
| FRAS1 | 2.815916501 | 8.769598342 | 10.7312039 | 3.03E-16 | 5.83E-14 | 26.64124806 |
| GSDME | 1.306959603 | 7.273984932 | 10.61896381 | 4.76E-16 | 8.45E-14 | 26.19808557 |
| UXS1 | 1.118731874 | 9.238103226 | 10.60571287 | 5.02E-16 | 8.83E-14 | 26.14568005 |
| TRPC1 | 1.545735983 | 6.854107497 | 10.49795991 | 7.74E-16 | 1.29E-13 | 25.7188679 |
| PARD3B | 1.318787099 | 8.8740613 | 10.48245857 | 8.24E-16 | 1.36E-13 | 25.65737027 |
| ABHD12B | 3.041104046 | 7.671592553 | 10.47174949 | 8.60E-16 | 1.41E-13 | 25.61487067 |
| NT5DC2 | 2.700446625 | 9.752747865 | 10.46206328 | 8.94E-16 | 1.44E-13 | 25.57642059 |
| MAGED2 | 1.298488751 | 9.447604888 | 10.43147014 | 1.01E-15 | 1.59E-13 | 25.45491779 |
| TTLL1 | 1.016886313 | 7.85932968 | 10.4010084 | 1.14E-15 | 1.78E-13 | 25.33384514 |
| TSPAN5 | 3.213198495 | 8.663672215 | 10.39767373 | 1.16E-15 | 1.79E-13 | 25.32058568 |
| RHNO1 | 1.299100036 | 9.068708937 | 10.38390765 | 1.23E-15 | 1.88E-13 | 25.26583698 |
| AXIN2 | 1.63073797 | 8.737350081 | 10.35157949 | 1.40E-15 | 2.12E-13 | 25.137193 |
| SNORD113-4 | 3.960466991 | 9.280339483 | 10.32702201 | 1.54E-15 | 2.29E-13 | 25.03940343 |
| PTPN14 | 1.64052097 | 9.148281833 | 10.29747056 | 1.74E-15 | 2.54E-13 | 24.92165083 |
| LRRC1 | 1.257251169 | 7.256897412 | 10.14204008 | 3.26E-15 | 4.58E-13 | 24.30095929 |
| SORT1 | 1.543479092 | 9.445655182 | 10.07989932 | 4.20E-15 | 5.70E-13 | 24.05218689 |
| SQLE | 2.562487443 | 9.74066658 | 10.07744702 | 4.24E-15 | 5.70E-13 | 24.04236231 |
| SLC7A11 | 3.486572778 | 7.692992929 | 9.920174064 | 8.05E-15 | 1.05E-12 | 23.4111828 |
| SNORD114-26 | 3.534836574 | 10.21168377 | 9.906794027 | 8.50E-15 | 1.10E-12 | 23.3573869 |
| POGLUT2 | 1.234990048 | 7.45065017 | 9.884434313 | 9.31E-15 | 1.18E-12 | 23.26745365 |
| CD34 | 1.592179453 | 9.260634247 | 9.882678463 | 9.38E-15 | 1.19E-12 | 23.26038966 |
| CD24 | 2.078500328 | 8.528922903 | 9.830922713 | 1.16E-14 | 1.45E-12 | 23.05205547 |
| GPX7 | 1.916614525 | 9.086789033 | 9.803986968 | 1.29E-14 | 1.58E-12 | 22.94354324 |
| FLVCR1 | 2.059286768 | 8.186015421 | 9.803643741 | 1.30E-14 | 1.58E-12 | 22.94216015 |
| ZNRF3 | 1.669130819 | 9.875832298 | 9.795971769 | 1.34E-14 | 1.60E-12 | 22.9112422 |
| SALL2 | 1.497471031 | 7.590890672 | 9.753148074 | 1.59E-14 | 1.87E-12 | 22.73857655 |
| H2AC6 | 1.271329892 | 9.700487468 | 9.750073406 | 1.61E-14 | 1.87E-12 | 22.72617383 |
| SNORD114-3 | 2.749127004 | 11.87352222 | 9.742676788 | 1.66E-14 | 1.91E-12 | 22.69633401 |
| IMPDH2 | 1.446152395 | 9.975528313 | 9.56719032 | 3.41E-14 | 3.71E-12 | 21.98714405 |
| HIF1AN | 1.100867652 | 8.24819784 | 9.54275014 | 3.77E-14 | 4.06E-12 | 21.88819254 |
| PAPSS1 | 1.221738479 | 8.015520585 | 9.531559408 | 3.95E-14 | 4.23E-12 | 21.84287004 |
| RAB34 | 1.910359669 | 8.611419238 | 9.529058629 | 3.99E-14 | 4.25E-12 | 21.83274065 |
| LYRM4 | 1.06762234 | 7.478073775 | 9.398124546 | 6.84E-14 | 6.95E-12 | 21.30178441 |
| LAMC1 | 1.412920452 | 9.937528934 | 9.333118638 | 8.95E-14 | 8.63E-12 | 21.03774858 |
| SLC1A4 | 1.616478529 | 9.36769174 | 9.289706088 | 1.07E-13 | 1.00E-11 | 20.86126824 |
| CCNB2 | 2.397618097 | 7.378167558 | 9.28840982 | 1.08E-13 | 1.00E-11 | 20.85599685 |
| HTR1D | 3.003341481 | 8.841416327 | 9.129503267 | 2.07E-13 | 1.81E-11 | 20.20902809 |
| ARMC9 | 1.023396066 | 7.083713704 | 9.129239477 | 2.08E-13 | 1.81E-11 | 20.2079529 |
| KIF20A | 2.301413056 | 6.965262136 | 9.109912016 | 2.25E-13 | 1.93E-11 | 20.12916459 |
| ZNF610 | 1.18913426 | 6.513622433 | 9.073730711 | 2.61E-13 | 2.18E-11 | 19.98161728 |
| NCAPD2 | 1.469891557 | 9.162615334 | 9.064981365 | 2.71E-13 | 2.26E-11 | 19.945927 |
| HMGA2 | 1.940571302 | 7.918837055 | 9.051784726 | 2.86E-13 | 2.36E-11 | 19.89208777 |
| ARL6IP6 | 1.256990044 | 7.972258617 | 9.008585293 | 3.42E-13 | 2.76E-11 | 19.71578149 |
| CDK6 | 1.276795869 | 9.079212796 | 8.9879258 | 3.73E-13 | 2.97E-11 | 19.63143248 |
| EPHB2 | 1.545548603 | 8.295195866 | 8.956655447 | 4.24E-13 | 3.33E-11 | 19.50372163 |
| ZBED8 | 1.626980376 | 5.510262325 | 8.936336415 | 4.61E-13 | 3.57E-11 | 19.42071195 |
| DPEP1 | 3.455693831 | 10.38352412 | 8.90068762 | 5.35E-13 | 4.06E-11 | 19.27502917 |
| PTP4A3 | 1.492437658 | 9.259241316 | 8.889928699 | 5.59E-13 | 4.21E-11 | 19.23105034 |
| ZSWIM5 | 1.811866754 | 9.156097342 | 8.859795434 | 6.34E-13 | 4.72E-11 | 19.10784863 |
| SMYD2 | 1.18343418 | 8.941259009 | 8.835452071 | 7.01E-13 | 5.14E-11 | 19.0082908 |
| PSPH | 1.579058465 | 8.831550262 | 8.820297825 | 7.47E-13 | 5.43E-11 | 18.94630147 |
| H3C10 | 1.748163592 | 7.559812675 | 8.801472866 | 8.08E-13 | 5.77E-11 | 18.86928379 |
| SPATS2 | 1.119820225 | 8.061799089 | 8.790212396 | 8.46E-13 | 5.99E-11 | 18.82320755 |
| ACLY | 1.135436034 | 9.78578876 | 8.784136334 | 8.68E-13 | 6.12E-11 | 18.79834309 |
| H2BC8 | 1.631395415 | 8.212752348 | 8.771893509 | 9.13E-13 | 6.36E-11 | 18.74823862 |
| GSN | 1.504072941 | 9.841387208 | 8.756669981 | 9.73E-13 | 6.71E-11 | 18.6859274 |
| RHOBTB1 | 1.617197447 | 8.864874519 | 8.753552719 | 9.85E-13 | 6.77E-11 | 18.67316709 |
| ST8SIA3 | 1.836569391 | 7.395722434 | 8.750438562 | 9.98E-13 | 6.77E-11 | 18.66041912 |
| DTL | 2.285317941 | 7.570949489 | 8.747879807 | 1.01E-12 | 6.79E-11 | 18.64994445 |
| CDKN3 | 2.175037651 | 6.233471444 | 8.747544912 | 1.01E-12 | 6.79E-11 | 18.64857349 |
| RNF157 | 1.533795655 | 8.428308207 | 8.726221992 | 1.10E-12 | 7.19E-11 | 18.56127504 |
| MPZL1 | 1.061881163 | 9.552405243 | 8.68091861 | 1.33E-12 | 8.47E-11 | 18.37574379 |
| SUPT3H | 1.340629299 | 7.091402614 | 8.646151201 | 1.54E-12 | 9.69E-11 | 18.23331312 |
| SRD5A3 | 1.162481738 | 8.371284681 | 8.641405054 | 1.57E-12 | 9.85E-11 | 18.21386666 |
| TARBP1 | 1.335892624 | 8.434325571 | 8.632248592 | 1.63E-12 | 1.02E-10 | 18.17634772 |
| H2AC8 | 2.0693819 | 6.43677705 | 8.60142759 | 1.85E-12 | 1.14E-10 | 18.05003843 |
| SNRPB | 1.308231607 | 10.4698593 | 8.586249757 | 1.98E-12 | 1.21E-10 | 17.98782683 |
| SDK1 | 1.504174346 | 8.453713777 | 8.572597504 | 2.09E-12 | 1.27E-10 | 17.93186265 |
| CCNA2 | 1.909675615 | 8.072862077 | 8.539140496 | 2.40E-12 | 1.43E-10 | 17.79469164 |
| PHYHIPL | 2.209353745 | 8.829288291 | 8.51284709 | 2.68E-12 | 1.57E-10 | 17.68687002 |
| MELK | 1.806620561 | 6.560644137 | 8.489226325 | 2.96E-12 | 1.71E-10 | 17.58999342 |
| PLPPR1 | 2.204949852 | 9.580004085 | 8.476374855 | 3.12E-12 | 1.79E-10 | 17.5372797 |
| UBE2T | 1.936367867 | 7.298125929 | 8.476093937 | 3.12E-12 | 1.79E-10 | 17.53612739 |
| UGT3A2 | 2.026999061 | 8.744543513 | 8.473487762 | 3.16E-12 | 1.80E-10 | 17.52543699 |
| H3C6 | 1.58529842 | 5.725392678 | 8.468109722 | 3.23E-12 | 1.83E-10 | 17.50337605 |
| H2BC5 | 1.643359553 | 8.052239929 | 8.450899607 | 3.47E-12 | 1.93E-10 | 17.43277514 |
| MAP7D2 | 1.330842158 | 6.453627136 | 8.447264771 | 3.52E-12 | 1.95E-10 | 17.41786316 |
| MFGE8 | 1.656925459 | 9.492723272 | 8.404442278 | 4.21E-12 | 2.29E-10 | 17.24216276 |
| USP11 | 1.185786329 | 9.758162388 | 8.39761695 | 4.33E-12 | 2.34E-10 | 17.21415519 |
| TP53I3 | 1.544392711 | 8.673499972 | 8.373315592 | 4.79E-12 | 2.55E-10 | 17.11442831 |
| ASPSCR1 | 1.473270029 | 9.845533002 | 8.315901566 | 6.09E-12 | 3.15E-10 | 16.8787768 |
| GJA5 | 1.438558726 | 7.098452815 | 8.314221367 | 6.13E-12 | 3.16E-10 | 16.87187982 |
| SLC44A3 | 1.295006028 | 8.317787321 | 8.307336937 | 6.31E-12 | 3.24E-10 | 16.84361981 |
| MMP11 | 1.905630712 | 9.158739896 | 8.28961971 | 6.79E-12 | 3.47E-10 | 16.77088929 |
| AACS | 1.228546065 | 8.163594841 | 8.288695321 | 6.82E-12 | 3.47E-10 | 16.7670945 |
| FAM169A | 1.689995381 | 7.247374689 | 8.2885956 | 6.82E-12 | 3.47E-10 | 16.76668512 |
| ETV1 | 1.572356803 | 8.191715725 | 8.256924267 | 7.79E-12 | 3.88E-10 | 16.63666244 |
| CDK4 | 1.194233101 | 10.81122527 | 8.240846746 | 8.33E-12 | 4.09E-10 | 16.57065422 |
| CAD | 1.432708509 | 8.936921038 | 8.218812688 | 9.13E-12 | 4.44E-10 | 16.48018696 |
| GINS1 | 2.308260456 | 7.812045639 | 8.204197913 | 9.70E-12 | 4.67E-10 | 16.42017964 |
| OLR1 | 2.92654584 | 8.16213212 | 8.187892089 | 1.04E-11 | 4.96E-10 | 16.35322727 |
| RHBG | 2.132733015 | 9.050887688 | 8.173041933 | 1.10E-11 | 5.22E-10 | 16.29225059 |
| CCND2 | 2.155315522 | 9.816418479 | 8.171645298 | 1.11E-11 | 5.24E-10 | 16.28651577 |
| H3C2 | 2.602281149 | 10.22520574 | 8.113719444 | 1.41E-11 | 6.48E-10 | 16.0486561 |
| MCM3 | 1.148280065 | 8.749225655 | 8.113173938 | 1.42E-11 | 6.48E-10 | 16.04641606 |
| DKK4 | 3.327005313 | 8.57414025 | 8.109221214 | 1.44E-11 | 6.55E-10 | 16.0301848 |
| CKAP2 | 1.681375739 | 7.477878595 | 8.106285517 | 1.46E-11 | 6.62E-10 | 16.0181298 |
| ARHGEF2 | 1.058366177 | 8.882719266 | 8.100271603 | 1.50E-11 | 6.77E-10 | 15.99343455 |
| AMER1 | 1.238651161 | 7.934665291 | 8.094795465 | 1.53E-11 | 6.89E-10 | 15.97094759 |
| TOP2A | 2.178370882 | 7.352340003 | 8.088462403 | 1.57E-11 | 7.05E-10 | 15.9449418 |
| LRP4 | 1.116526169 | 8.311412317 | 8.067040546 | 1.72E-11 | 7.61E-10 | 15.85697634 |
| EHMT2 | 1.285521952 | 8.718175866 | 8.058932232 | 1.78E-11 | 7.82E-10 | 15.82368103 |
| FKBP10 | 2.268022124 | 10.32415787 | 8.052721873 | 1.82E-11 | 7.97E-10 | 15.79817944 |
| PFKM | 1.384918067 | 8.066138016 | 8.047875349 | 1.86E-11 | 8.11E-10 | 15.77827824 |
| PRIM1 | 1.160490681 | 7.958373033 | 8.000953818 | 2.26E-11 | 9.60E-10 | 15.58561012 |
| B3GALNT1 | 1.190443847 | 6.24832069 | 7.998820955 | 2.28E-11 | 9.64E-10 | 15.57685248 |
| EDARADD | 1.337316446 | 7.876570793 | 7.996245058 | 2.31E-11 | 9.67E-10 | 15.56627577 |
| MKI67 | 1.637800832 | 7.439672602 | 7.98802581 | 2.39E-11 | 9.95E-10 | 15.53252757 |
| CDK1 | 2.083547989 | 5.879456857 | 7.987014948 | 2.40E-11 | 9.97E-10 | 15.52837701 |
| MCM6 | 1.37936676 | 7.190468651 | 7.966499654 | 2.61E-11 | 1.07E-09 | 15.44414366 |
| PTK7 | 1.61061442 | 8.72176737 | 7.946155597 | 2.84E-11 | 1.15E-09 | 15.36061697 |
| MAPK13 | 1.272749762 | 8.40757739 | 7.935601753 | 2.97E-11 | 1.20E-09 | 15.3172876 |
| NREP | 1.716951754 | 10.81496699 | 7.931711322 | 3.02E-11 | 1.21E-09 | 15.30131552 |
| MSH2 | 1.268386096 | 7.26479822 | 7.929166897 | 3.05E-11 | 1.22E-09 | 15.29086953 |
| SNORD113-3 | 2.732897264 | 7.538923474 | 7.908065255 | 3.33E-11 | 1.32E-09 | 15.20424085 |
| FANCI | 1.474634584 | 6.859873607 | 7.906127895 | 3.36E-11 | 1.33E-09 | 15.19628766 |
| TRIM59 | 1.321362731 | 5.476907243 | 7.896115172 | 3.51E-11 | 1.38E-09 | 15.15518456 |
| XRCC1 | 1.004380408 | 9.212673099 | 7.889051404 | 3.61E-11 | 1.42E-09 | 15.12618797 |
| SULT1C2 | 2.070616805 | 7.368448492 | 7.879906736 | 3.75E-11 | 1.46E-09 | 15.08865039 |
| TUBG1 | 1.118529969 | 10.522168 | 7.878779218 | 3.77E-11 | 1.46E-09 | 15.08402216 |
| TMEM182 | 1.072893011 | 6.348806681 | 7.867353428 | 3.95E-11 | 1.52E-09 | 15.0371228 |
| PCGF2 | 1.197195547 | 9.167694998 | 7.863480717 | 4.02E-11 | 1.54E-09 | 15.02122696 |
| TRIM28 | 1.182951146 | 10.62560195 | 7.861057295 | 4.06E-11 | 1.56E-09 | 15.01127996 |
| ASIC1 | 1.90999154 | 7.451810327 | 7.84022611 | 4.42E-11 | 1.68E-09 | 14.92578174 |
| BEX1 | 2.709836374 | 8.921574751 | 7.839111158 | 4.45E-11 | 1.68E-09 | 14.9212058 |
| H2AZ1 | 1.197634527 | 8.97041182 | 7.828713113 | 4.64E-11 | 1.75E-09 | 14.8785317 |
| ZC3HAV1L | 1.292177971 | 8.490300499 | 7.797957345 | 5.28E-11 | 1.95E-09 | 14.75232026 |
| PLAG1 | 1.477146037 | 7.247707466 | 7.783970596 | 5.59E-11 | 2.04E-09 | 14.69492954 |
| C1orf74 | 1.202389576 | 8.020362028 | 7.780651207 | 5.67E-11 | 2.06E-09 | 14.68130996 |
| PLK1 | 2.412514592 | 8.921564979 | 7.778641917 | 5.72E-11 | 2.07E-09 | 14.67306588 |
| SLC26A2 | 1.108743685 | 6.818203595 | 7.778174042 | 5.73E-11 | 2.07E-09 | 14.67114621 |
| NDRG3 | 1.034632045 | 9.285799135 | 7.778170597 | 5.73E-11 | 2.07E-09 | 14.67113207 |
| THY1 | 1.832923323 | 8.592422408 | 7.745506569 | 6.57E-11 | 2.32E-09 | 14.53712528 |
| TGFB2 | 2.264112043 | 8.687769178 | 7.731206915 | 6.97E-11 | 2.43E-09 | 14.47846775 |
| SNTG1 | 1.792241852 | 7.246158613 | 7.730172391 | 7.00E-11 | 2.44E-09 | 14.47422431 |
| CDH13 | 1.297535823 | 8.080990611 | 7.721865303 | 7.25E-11 | 2.52E-09 | 14.44015101 |
| CACNB4 | 1.459895385 | 7.576951955 | 7.712639118 | 7.53E-11 | 2.61E-09 | 14.40230993 |
| FIGN | 1.827531693 | 9.093064388 | 7.696676428 | 8.05E-11 | 2.75E-09 | 14.33684454 |
| ZMYM3 | 1.119125701 | 9.078646419 | 7.691823298 | 8.21E-11 | 2.80E-09 | 14.31694251 |
| SOBP | 1.144253776 | 8.288161076 | 7.677218691 | 8.73E-11 | 2.96E-09 | 14.25705502 |
| ANLN | 1.945267933 | 6.717726716 | 7.664091711 | 9.22E-11 | 3.09E-09 | 14.20323198 |
| WDCP | 1.019700158 | 7.528419069 | 7.658404653 | 9.44E-11 | 3.16E-09 | 14.1799156 |
| REG3A | 4.576358499 | 10.24968529 | 7.646724657 | 9.91E-11 | 3.30E-09 | 14.13203187 |
| H4C9 | 1.173232502 | 6.468999975 | 7.644356248 | 1.00E-10 | 3.32E-09 | 14.12232278 |
| FOXM1 | 2.024086663 | 7.879821567 | 7.642277906 | 1.01E-10 | 3.33E-09 | 14.11380294 |
| HDAC11 | 1.28362557 | 7.971310378 | 7.633178877 | 1.05E-10 | 3.45E-09 | 14.0765045 |
| PDGFRB | 1.399527244 | 9.655076773 | 7.615742961 | 1.13E-10 | 3.69E-09 | 14.00503934 |
| ATP6AP1 | 1.010988909 | 10.95766149 | 7.612381873 | 1.14E-10 | 3.73E-09 | 13.99126431 |
| FGD1 | 1.012588005 | 7.945458662 | 7.599819905 | 1.21E-10 | 3.92E-09 | 13.93978397 |
| RFC3 | 1.102139322 | 7.554680957 | 7.597366102 | 1.22E-10 | 3.95E-09 | 13.92972866 |
| PARPBP | 1.631499511 | 5.258048079 | 7.580097043 | 1.31E-10 | 4.20E-09 | 13.85896871 |
| HELLS | 1.678850919 | 6.072627546 | 7.570343627 | 1.36E-10 | 4.36E-09 | 13.8190089 |
| ATP8B2 | 1.217890176 | 8.847461136 | 7.567573673 | 1.38E-10 | 4.40E-09 | 13.80766103 |
| TICRR | 1.787516611 | 7.272720925 | 7.555890442 | 1.45E-10 | 4.59E-09 | 13.75980065 |
| CHEK1 | 1.489541296 | 7.019705634 | 7.554104221 | 1.46E-10 | 4.62E-09 | 13.75248386 |
| CCNB1 | 1.675570182 | 6.930587494 | 7.529864594 | 1.61E-10 | 5.08E-09 | 13.65320479 |
| ACSL4 | 2.191079707 | 9.457615927 | 7.52499403 | 1.65E-10 | 5.15E-09 | 13.63325909 |
| H2BC7 | 2.294919807 | 9.098110331 | 7.499824646 | 1.83E-10 | 5.69E-09 | 13.53020227 |
| AURKA | 1.697446459 | 7.44579256 | 7.487797759 | 1.92E-10 | 5.96E-09 | 13.48096733 |
| ALDH18A1 | 1.162397337 | 8.79215765 | 7.483875828 | 1.95E-10 | 6.05E-09 | 13.46491333 |
| IGDCC3 | 1.94210042 | 8.877011638 | 7.481586331 | 1.97E-10 | 6.07E-09 | 13.45554183 |
| TET1 | 1.198754251 | 7.16143004 | 7.476329637 | 2.02E-10 | 6.19E-09 | 13.43402572 |
| HS6ST1 | 1.227912896 | 10.53277823 | 7.475858817 | 2.02E-10 | 6.19E-09 | 13.43209867 |
| ZNF704 | 1.165593036 | 8.31500024 | 7.475768322 | 2.02E-10 | 6.19E-09 | 13.43172828 |
| ALYREF | 1.222367921 | 10.45768225 | 7.474512917 | 2.03E-10 | 6.21E-09 | 13.42659002 |
| PIGU | 1.254642271 | 9.391688164 | 7.464247504 | 2.12E-10 | 6.45E-09 | 13.3845772 |
| CDCA7 | 1.889775509 | 7.007827972 | 7.450454263 | 2.24E-10 | 6.82E-09 | 13.32813379 |
| LAPTM4B | 1.639573557 | 10.43569631 | 7.440115379 | 2.34E-10 | 7.03E-09 | 13.2858318 |
| SKA3 | 1.938216705 | 6.328014231 | 7.410951341 | 2.65E-10 | 7.84E-09 | 13.16653363 |
| HUNK | 1.463194082 | 7.728366221 | 7.408152105 | 2.68E-10 | 7.92E-09 | 13.1550853 |
| SKP2 | 1.076306002 | 10.02341392 | 7.394186729 | 2.84E-10 | 8.34E-09 | 13.09797557 |
| CDON | 1.068972884 | 8.224238256 | 7.390359385 | 2.88E-10 | 8.45E-09 | 13.08232585 |
| ASPM | 1.895340459 | 6.398222509 | 7.381832722 | 2.99E-10 | 8.69E-09 | 13.04746369 |
| PBK | 1.767009814 | 5.685898004 | 7.374557487 | 3.08E-10 | 8.90E-09 | 13.01772114 |
| RPS5 | 1.185687416 | 11.53297067 | 7.3708092 | 3.13E-10 | 9.02E-09 | 13.00239851 |
| ZNF362 | 1.043221957 | 9.347383559 | 7.369457767 | 3.14E-10 | 9.05E-09 | 12.99687418 |
| MEP1A | 3.188203912 | 7.479082024 | 7.362004045 | 3.24E-10 | 9.29E-09 | 12.96640688 |
| MFAP2 | 1.794715433 | 9.148296429 | 7.354102916 | 3.35E-10 | 9.54E-09 | 12.93411407 |
| TCTN2 | 1.248935615 | 7.642745262 | 7.352537366 | 3.37E-10 | 9.59E-09 | 12.92771589 |
| PRC1 | 1.531223139 | 7.539632282 | 7.347896641 | 3.44E-10 | 9.75E-09 | 12.90875072 |
| SLC25A36 | 1.184247511 | 7.836810236 | 7.340253356 | 3.55E-10 | 1.00E-08 | 12.87751762 |
| ADAM32 | 1.028802449 | 6.101341621 | 7.305914445 | 4.09E-10 | 1.14E-08 | 12.73723779 |
| RRM2 | 1.536782251 | 7.740766954 | 7.298827027 | 4.22E-10 | 1.17E-08 | 12.70829309 |
| TMEM245 | 1.073459239 | 10.96713326 | 7.293212879 | 4.32E-10 | 1.19E-08 | 12.68536728 |
| H4C5 | 1.442926631 | 9.568467468 | 7.285673789 | 4.45E-10 | 1.22E-08 | 12.65458378 |
| PTTG1 | 1.457747189 | 7.281470615 | 7.265140165 | 4.85E-10 | 1.33E-08 | 12.57075873 |
| MCM7 | 1.255867325 | 9.402161157 | 7.248290669 | 5.20E-10 | 1.41E-08 | 12.50199307 |
| ETV4 | 2.034290051 | 8.611755648 | 7.240135692 | 5.38E-10 | 1.46E-08 | 12.46871768 |
| NDN | 1.28306659 | 6.562040801 | 7.219646542 | 5.86E-10 | 1.58E-08 | 12.38513322 |
| E2F5 | 1.452304495 | 7.518878813 | 7.213541873 | 6.01E-10 | 1.62E-08 | 12.36023486 |
| PRKAA2 | 1.836274412 | 8.581348209 | 7.186046551 | 6.73E-10 | 1.79E-08 | 12.24812418 |
| C9orf152 | 1.373523746 | 5.622290581 | 7.182922424 | 6.82E-10 | 1.81E-08 | 12.23538899 |
| TOX3 | 1.252327437 | 9.194053982 | 7.179535154 | 6.92E-10 | 1.83E-08 | 12.2215819 |
| ZWINT | 1.06849927 | 7.655972174 | 7.158739134 | 7.54E-10 | 1.97E-08 | 12.13683136 |
| H2BC14 | 2.665078243 | 10.75311256 | 7.148886864 | 7.86E-10 | 2.04E-08 | 12.09669091 |
| PRTG | 2.245048981 | 7.582425393 | 7.148708985 | 7.86E-10 | 2.04E-08 | 12.09596625 |
| PLAGL2 | 1.081327318 | 8.393528886 | 7.146536045 | 7.93E-10 | 2.05E-08 | 12.08711416 |
| BCAM | 1.525041958 | 9.965304925 | 7.119637106 | 8.87E-10 | 2.28E-08 | 11.97756226 |
| TTLL4 | 1.214984511 | 9.635701067 | 7.104113824 | 9.46E-10 | 2.41E-08 | 11.91436483 |
| USP21 | 1.047938434 | 8.507815308 | 7.084799219 | 1.02E-09 | 2.58E-08 | 11.83575814 |
| ERVMER34-1 | 2.071770702 | 7.818499008 | 7.063656566 | 1.12E-09 | 2.79E-08 | 11.74974507 |
| STOX1 | 1.559991387 | 5.719685008 | 7.015800741 | 1.36E-09 | 3.33E-08 | 11.55519034 |
| LYZ | 1.469969285 | 11.96324396 | 7.010080114 | 1.40E-09 | 3.41E-08 | 11.53194624 |
| H4C15 | 2.223115185 | 9.904898186 | 7.006895933 | 1.41E-09 | 3.44E-08 | 11.51900946 |
| H4C14 | 2.223115185 | 9.904898186 | 7.006895933 | 1.41E-09 | 3.44E-08 | 11.51900946 |
| MMS22L | 1.23222546 | 6.078946658 | 6.999206753 | 1.46E-09 | 3.53E-08 | 11.4877732 |
| CCDC34 | 1.119582803 | 6.971746912 | 6.991635011 | 1.51E-09 | 3.62E-08 | 11.45701899 |
| SLC29A4 | 1.772143999 | 9.405481036 | 6.970933353 | 1.64E-09 | 3.90E-08 | 11.37296038 |
| H2BC3 | 2.339806262 | 7.842291941 | 6.965098533 | 1.68E-09 | 3.98E-08 | 11.34927505 |
| H3C11 | 2.472319939 | 10.8070386 | 6.948611254 | 1.80E-09 | 4.22E-08 | 11.28236459 |
| WDR76 | 1.306458708 | 7.189942473 | 6.937288468 | 1.89E-09 | 4.40E-08 | 11.2364275 |
| NCAPG | 1.493597014 | 6.60389155 | 6.921871871 | 2.01E-09 | 4.68E-08 | 11.17390057 |
| RASL11B | 1.624823556 | 7.583604515 | 6.91096736 | 2.10E-09 | 4.86E-08 | 11.1296872 |
| CENPK | 1.539351267 | 6.529929748 | 6.902889379 | 2.17E-09 | 5.01E-08 | 11.0969415 |
| CDC20 | 1.750980359 | 8.074611253 | 6.892814248 | 2.27E-09 | 5.19E-08 | 11.05610865 |
| SCML2 | 1.066559831 | 6.809152021 | 6.876046902 | 2.43E-09 | 5.52E-08 | 10.98817502 |
| PXYLP1 | 1.045977505 | 7.014301165 | 6.874160611 | 2.45E-09 | 5.55E-08 | 10.98053435 |
| GSTA4 | 1.0761372 | 8.184457853 | 6.870182578 | 2.49E-09 | 5.61E-08 | 10.96442192 |
| H2AC16 | 1.645580868 | 7.197654709 | 6.868219647 | 2.51E-09 | 5.65E-08 | 10.95647194 |
| ENPP3 | 1.735153864 | 8.487844937 | 6.855781533 | 2.64E-09 | 5.90E-08 | 10.9061057 |
| MEG3 | 1.827333711 | 9.356971829 | 6.852772011 | 2.67E-09 | 5.96E-08 | 10.89392142 |
| ZNF711 | 1.443436117 | 6.736636205 | 6.85270933 | 2.67E-09 | 5.96E-08 | 10.89366766 |
| MAD2L1 | 1.35632512 | 6.021124834 | 6.848953445 | 2.72E-09 | 6.03E-08 | 10.87846296 |
| TTYH3 | 1.150129393 | 9.310792584 | 6.839402442 | 2.82E-09 | 6.27E-08 | 10.83980464 |
| SPC24 | 1.499822071 | 6.933637084 | 6.835681376 | 2.87E-09 | 6.35E-08 | 10.82474588 |
| POLE2 | 1.154084258 | 6.648787515 | 6.815162487 | 3.12E-09 | 6.85E-08 | 10.74173349 |
| H2BC11 | 1.022803156 | 6.179716574 | 6.806472259 | 3.24E-09 | 7.08E-08 | 10.70658891 |
| C2orf15 | 1.101967574 | 5.394606119 | 6.805257305 | 3.25E-09 | 7.11E-08 | 10.70167608 |
| STIL | 1.239510227 | 6.38863397 | 6.794571236 | 3.40E-09 | 7.33E-08 | 10.65847221 |
| LIX1 | 2.093101776 | 6.778415464 | 6.773582803 | 3.70E-09 | 7.91E-08 | 10.57365104 |
| IGF2BP3 | 1.643654347 | 7.165708886 | 6.770880608 | 3.75E-09 | 7.99E-08 | 10.562734 |
| CENPI | 1.445421752 | 6.459178183 | 6.768600793 | 3.78E-09 | 8.02E-08 | 10.55352403 |
| SKA1 | 1.258371464 | 5.608117734 | 6.761316341 | 3.90E-09 | 8.22E-08 | 10.52410013 |
| H2AC13 | 2.086057055 | 8.211234827 | 6.749095296 | 4.10E-09 | 8.56E-08 | 10.47474901 |
| TRIP13 | 1.095340944 | 6.83221209 | 6.748245819 | 4.11E-09 | 8.56E-08 | 10.47131925 |
| AFP | 3.925359024 | 8.614203475 | 6.736301219 | 4.32E-09 | 8.93E-08 | 10.42310149 |
| TMEM52B | 1.873421578 | 7.015910498 | 6.732719631 | 4.38E-09 | 9.04E-08 | 10.40864649 |
| SNORD95 | 1.051679149 | 10.5484478 | 6.724233509 | 4.54E-09 | 9.32E-08 | 10.37440292 |
| H3C7 | 1.794102201 | 9.024982658 | 6.712216695 | 4.77E-09 | 9.78E-08 | 10.32592605 |
| MCM5 | 1.229977963 | 8.83867425 | 6.700754193 | 5.00E-09 | 1.02E-07 | 10.27970064 |
| B4GALT6 | 1.048521957 | 7.105420458 | 6.678377464 | 5.48E-09 | 1.10E-07 | 10.18950462 |
| EZH2 | 1.061573609 | 7.621863301 | 6.677832057 | 5.49E-09 | 1.11E-07 | 10.18730692 |
| NCAPH | 1.366337145 | 7.061869824 | 6.668137891 | 5.72E-09 | 1.14E-07 | 10.14825052 |
| H1-5 | 2.522239203 | 8.42308077 | 6.665900685 | 5.77E-09 | 1.15E-07 | 10.13923871 |
| EIPR1 | 1.014197491 | 7.99749284 | 6.665055673 | 5.79E-09 | 1.15E-07 | 10.13583503 |
| MCM2 | 1.219664012 | 8.212707569 | 6.661768516 | 5.87E-09 | 1.17E-07 | 10.12259527 |
| PCSK5 | 1.143032216 | 7.901310762 | 6.659422676 | 5.92E-09 | 1.18E-07 | 10.11314767 |
| WFS1 | 1.210033571 | 8.919670455 | 6.653566787 | 6.07E-09 | 1.20E-07 | 10.0895666 |
| MATN3 | 2.813506747 | 7.705570569 | 6.651321151 | 6.12E-09 | 1.21E-07 | 10.08052474 |
| SPC25 | 1.592386507 | 5.675024843 | 6.623571631 | 6.86E-09 | 1.33E-07 | 9.968844111 |
| SV2A | 1.37462147 | 7.670621228 | 6.61818043 | 7.02E-09 | 1.36E-07 | 9.947157623 |
| KIF11 | 1.554600523 | 6.217780816 | 6.612908153 | 7.17E-09 | 1.38E-07 | 9.925952991 |
| KNTC1 | 1.240726417 | 6.564524173 | 6.611021277 | 7.23E-09 | 1.39E-07 | 9.918364978 |
| PGAP1 | 1.501023826 | 7.747815787 | 6.610499081 | 7.24E-09 | 1.39E-07 | 9.916265063 |
| TBX3 | 1.024881784 | 9.55760507 | 6.609925137 | 7.26E-09 | 1.39E-07 | 9.913957092 |
| ITM2C | 1.068815532 | 10.72478684 | 6.604471471 | 7.42E-09 | 1.42E-07 | 9.892028586 |
| H2AC12 | 1.546565093 | 6.277461214 | 6.598553049 | 7.60E-09 | 1.45E-07 | 9.868235566 |
| ZNF382 | 1.204667502 | 6.697680975 | 6.584980781 | 8.04E-09 | 1.52E-07 | 9.813689494 |
| PCLAF | 1.269617309 | 6.697329504 | 6.581092595 | 8.17E-09 | 1.55E-07 | 9.798067429 |
| BCL9 | 1.090415746 | 8.523346012 | 6.574045028 | 8.41E-09 | 1.59E-07 | 9.769756428 |
| PLVAP | 1.064967404 | 8.633254673 | 6.573754216 | 8.42E-09 | 1.59E-07 | 9.768588336 |
| TMEM237 | 1.028932526 | 7.105222955 | 6.570224464 | 8.54E-09 | 1.60E-07 | 9.754411381 |
| H3C8 | 1.424519146 | 7.072705305 | 6.545384866 | 9.46E-09 | 1.76E-07 | 9.654690816 |
| CCDC88C | 1.416030529 | 7.917079793 | 6.536716237 | 9.80E-09 | 1.81E-07 | 9.619908888 |
| PRR11 | 1.290242999 | 7.498320314 | 6.525083464 | 1.03E-08 | 1.88E-07 | 9.573249288 |
| XRCC2 | 1.484714456 | 6.58922937 | 6.516434512 | 1.06E-08 | 1.95E-07 | 9.53856963 |
| P4HA2 | 1.076170124 | 8.461613617 | 6.515383448 | 1.07E-08 | 1.95E-07 | 9.534355868 |
| H2BC9 | 2.061364185 | 10.19698691 | 6.505616353 | 1.11E-08 | 2.02E-07 | 9.495206274 |
| CENPF | 1.727723425 | 7.014690113 | 6.498267573 | 1.15E-08 | 2.07E-07 | 9.465758565 |
| NUF2 | 1.680914716 | 5.924063259 | 6.490782799 | 1.18E-08 | 2.12E-07 | 9.435773481 |
| NRSN2 | 1.247722978 | 8.312428727 | 6.483676031 | 1.22E-08 | 2.17E-07 | 9.407309851 |
| FERMT1 | 1.462675728 | 6.651096937 | 6.475974101 | 1.26E-08 | 2.23E-07 | 9.376470382 |
| H2AC17 | 1.546848708 | 6.419658763 | 6.475685067 | 1.26E-08 | 2.23E-07 | 9.375313215 |
| CDCA5 | 1.112635096 | 6.827956303 | 6.470202563 | 1.29E-08 | 2.28E-07 | 9.353365828 |
| H1-2 | 1.40189956 | 9.088427693 | 6.462113272 | 1.33E-08 | 2.34E-07 | 9.320990695 |
| TBX4 | 2.03800573 | 8.182585813 | 6.461302803 | 1.33E-08 | 2.35E-07 | 9.317747526 |
| DRAM1 | 1.02057435 | 8.292062554 | 6.456055382 | 1.36E-08 | 2.39E-07 | 9.296751685 |
| COLEC12 | 1.38341188 | 8.362657272 | 6.448187848 | 1.41E-08 | 2.45E-07 | 9.2652796 |
| FOXRED2 | 1.078138073 | 9.223806884 | 6.446713191 | 1.42E-08 | 2.46E-07 | 9.259381584 |
| KIFC1 | 1.209645729 | 7.332400645 | 6.433108244 | 1.50E-08 | 2.58E-07 | 9.204982026 |
| FANCG | 1.064700744 | 7.642002839 | 6.421781836 | 1.57E-08 | 2.69E-07 | 9.159713481 |
| MND1 | 1.356836737 | 5.5358125 | 6.420442137 | 1.58E-08 | 2.70E-07 | 9.154360297 |
| DEPDC1B | 1.33274055 | 6.905502028 | 6.414199871 | 1.62E-08 | 2.76E-07 | 9.129420808 |
| SINHCAF | 1.027836378 | 8.66465629 | 6.409911842 | 1.64E-08 | 2.80E-07 | 9.112292297 |
| CDC6 | 1.515149505 | 6.192810153 | 6.407893504 | 1.66E-08 | 2.82E-07 | 9.104230983 |
| LINC01124 | 1.130293266 | 7.19788282 | 6.403776686 | 1.69E-08 | 2.86E-07 | 9.08779011 |
| FDFT1 | 1.073323797 | 11.50647908 | 6.399972951 | 1.71E-08 | 2.89E-07 | 9.072601775 |
| FAM111B | 1.16597456 | 6.281557641 | 6.388170967 | 1.80E-08 | 3.02E-07 | 9.02548996 |
| TP53 | 1.083074351 | 9.802222586 | 6.383955666 | 1.83E-08 | 3.05E-07 | 9.008668086 |
| NETO2 | 1.615814259 | 7.560957864 | 6.378895699 | 1.87E-08 | 3.10E-07 | 8.988478928 |
| EXO1 | 1.038203482 | 6.499214034 | 6.375011469 | 1.90E-08 | 3.15E-07 | 8.97298353 |
| MCM4 | 1.212793479 | 8.014596159 | 6.370562236 | 1.93E-08 | 3.20E-07 | 8.955236938 |
| SGO1 | 1.406933124 | 5.865334342 | 6.366917905 | 1.96E-08 | 3.24E-07 | 8.940703066 |
| TPX2 | 1.634283428 | 7.626301766 | 6.354705812 | 2.06E-08 | 3.39E-07 | 8.892014928 |
| CDC25C | 1.143428911 | 6.38596145 | 6.345165495 | 2.14E-08 | 3.50E-07 | 8.853994621 |
| ZNF660 | 1.048314404 | 5.839213728 | 6.340475675 | 2.18E-08 | 3.56E-07 | 8.835309741 |
| CDCA8 | 1.082763633 | 7.25089669 | 6.323571877 | 2.34E-08 | 3.79E-07 | 8.767990862 |
| NPM3 | 1.05383911 | 8.810164773 | 6.316412834 | 2.41E-08 | 3.89E-07 | 8.739493562 |
| H2BC13 | 1.814727293 | 7.214600455 | 6.309957947 | 2.47E-08 | 3.96E-07 | 8.713806106 |
| PIK3R2 | 1.065062966 | 9.011934836 | 6.303394429 | 2.54E-08 | 4.06E-07 | 8.687693063 |
| H4C4 | 1.666962846 | 8.32476488 | 6.299061108 | 2.58E-08 | 4.12E-07 | 8.670456618 |
| SULT1C4 | 1.392258944 | 7.564990297 | 6.297506326 | 2.60E-08 | 4.14E-07 | 8.664272959 |
| ASF1B | 1.352973027 | 7.76515071 | 6.295116691 | 2.62E-08 | 4.17E-07 | 8.654769682 |
| CENPO | 1.062204179 | 7.867383471 | 6.292405299 | 2.65E-08 | 4.21E-07 | 8.64398792 |
| H2AC21 | 1.908358683 | 10.10396255 | 6.279204777 | 2.80E-08 | 4.41E-07 | 8.591513229 |
| DLGAP5 | 1.598257327 | 5.998263909 | 6.277872865 | 2.81E-08 | 4.43E-07 | 8.586220165 |
| SNORD52 | 1.248559439 | 7.478955027 | 6.263559086 | 2.98E-08 | 4.68E-07 | 8.529354805 |
| SNORD114-2 | 2.701287435 | 7.046828445 | 6.257214189 | 3.06E-08 | 4.77E-07 | 8.504158619 |
| BUB1 | 1.51024697 | 6.570481062 | 6.249438705 | 3.16E-08 | 4.91E-07 | 8.473290401 |
| RAVER2 | 1.06735718 | 7.404150224 | 6.24884892 | 3.17E-08 | 4.92E-07 | 8.470949394 |
| C1orf198 | 1.070806306 | 9.54969002 | 6.244188541 | 3.23E-08 | 4.99E-07 | 8.452453167 |
| CBX5 | 1.134068619 | 9.970525432 | 6.23993227 | 3.28E-08 | 5.06E-07 | 8.435563902 |
| H2AC11 | 1.108508415 | 8.137683582 | 6.229114986 | 3.43E-08 | 5.27E-07 | 8.392653444 |
| SERPINI1 | 2.144525886 | 6.642080699 | 6.226112952 | 3.47E-08 | 5.33E-07 | 8.380748297 |
| MME | 2.451658134 | 8.542176731 | 6.195445415 | 3.93E-08 | 5.98E-07 | 8.259216966 |
| MAP4K4 | 1.071879531 | 9.733696658 | 6.195050542 | 3.94E-08 | 5.99E-07 | 8.257653176 |
| FKBP14 | 1.03566954 | 6.845459514 | 6.19383643 | 3.96E-08 | 6.01E-07 | 8.252845172 |
| GSTP1 | 1.66020402 | 11.36226571 | 6.190272162 | 4.01E-08 | 6.08E-07 | 8.23873177 |
| ZNF503 | 1.025222019 | 8.734343453 | 6.189876726 | 4.02E-08 | 6.08E-07 | 8.237166097 |
| SOAT2 | 1.833173819 | 8.652755057 | 6.177544819 | 4.23E-08 | 6.33E-07 | 8.188353195 |
| GINS2 | 1.231806866 | 8.071692566 | 6.175457913 | 4.26E-08 | 6.37E-07 | 8.180095267 |
| RAPGEFL1 | 1.227957869 | 7.364424969 | 6.17043084 | 4.35E-08 | 6.47E-07 | 8.160206133 |
| CDCA2 | 1.161944943 | 6.306372083 | 6.166364204 | 4.42E-08 | 6.56E-07 | 8.144120084 |
| NEIL3 | 1.139220968 | 6.454728963 | 6.164189243 | 4.46E-08 | 6.61E-07 | 8.13551795 |
| DIAPH3 | 1.035698802 | 5.906704568 | 6.147331658 | 4.78E-08 | 7.02E-07 | 8.068872993 |
| H2BC6 | 1.277842639 | 6.580025237 | 6.141079848 | 4.90E-08 | 7.17E-07 | 8.044169716 |
| TMEM99 | 1.14348917 | 6.392879102 | 6.133388921 | 5.05E-08 | 7.38E-07 | 8.01378943 |
| LSM2 | 1.126437885 | 8.535605476 | 6.126027214 | 5.20E-08 | 7.58E-07 | 7.984719461 |
| KIF23 | 1.076823247 | 6.611269063 | 6.103461059 | 5.70E-08 | 8.22E-07 | 7.895670589 |
| H2AC4 | 1.891377046 | 7.959129565 | 6.096701879 | 5.86E-08 | 8.42E-07 | 7.869015934 |
| NUSAP1 | 1.093642275 | 7.899227438 | 6.079294612 | 6.28E-08 | 8.93E-07 | 7.800409235 |
| KPNA2 | 1.262954123 | 10.0227682 | 6.045341063 | 7.20E-08 | 1.01E-06 | 7.666750512 |
| SPDL1 | 1.190062366 | 6.470439703 | 6.045179687 | 7.21E-08 | 1.01E-06 | 7.666115765 |
| CKB | 1.212793145 | 9.292614163 | 6.034042704 | 7.54E-08 | 1.05E-06 | 7.622322264 |
| NDC80 | 1.143425667 | 5.570586452 | 6.028626414 | 7.71E-08 | 1.07E-06 | 7.60103251 |
| RAD51AP1 | 1.157837276 | 5.769371197 | 6.024459191 | 7.84E-08 | 1.08E-06 | 7.584656252 |
| COL15A1 | 1.101889796 | 7.773260992 | 6.021857405 | 7.92E-08 | 1.09E-06 | 7.574433494 |
| KIF18A | 1.313615383 | 5.614740837 | 5.996727554 | 8.76E-08 | 1.19E-06 | 7.475761999 |
| NAP1L1 | 1.149112145 | 10.25900323 | 5.995240714 | 8.81E-08 | 1.20E-06 | 7.469927801 |
| VWCE | 1.064250556 | 8.471180107 | 5.99514376 | 8.81E-08 | 1.20E-06 | 7.469547381 |
| ECT2 | 1.256470993 | 6.446833202 | 5.989317042 | 9.02E-08 | 1.21E-06 | 7.446688234 |
| RELN | 1.418072197 | 11.67269886 | 5.981987651 | 9.29E-08 | 1.24E-06 | 7.417943282 |
| SNORD114-6 | 1.824692758 | 5.993562166 | 5.981940201 | 9.29E-08 | 1.24E-06 | 7.417757221 |
| HNF1A-AS1 | 2.063082343 | 9.603599215 | 5.967065522 | 9.87E-08 | 1.31E-06 | 7.359453193 |
| ERCC2 | 1.048327328 | 8.847331394 | 5.9642879 | 9.98E-08 | 1.32E-06 | 7.34857064 |
| ZNF738 | 1.382233628 | 7.599726719 | 5.956758877 | 1.03E-07 | 1.36E-06 | 7.319080091 |
| GLMP | 1.163117924 | 10.77015723 | 5.955582863 | 1.03E-07 | 1.36E-06 | 7.314474763 |
| LOC339803 | 1.036189558 | 7.469886512 | 5.932698882 | 1.13E-07 | 1.48E-06 | 7.22491515 |
| WDHD1 | 1.074777091 | 6.075753826 | 5.911252291 | 1.23E-07 | 1.59E-06 | 7.141077144 |
| RPL22L1 | 1.324942678 | 6.736141822 | 5.910983102 | 1.24E-07 | 1.59E-06 | 7.140025438 |
| HJURP | 1.194229559 | 7.369618063 | 5.908782064 | 1.25E-07 | 1.60E-06 | 7.131426681 |
| FBLN1 | 1.423720705 | 9.169655894 | 5.903433486 | 1.27E-07 | 1.63E-06 | 7.110535636 |
| ENAH | 1.139484218 | 8.222113003 | 5.884019626 | 1.38E-07 | 1.74E-06 | 7.034756587 |
| METTL24 | 1.032884004 | 6.494797248 | 5.876784832 | 1.42E-07 | 1.79E-06 | 7.006536723 |
| PYCR1 | 1.416198575 | 8.735784005 | 5.868565593 | 1.46E-07 | 1.84E-06 | 6.974490259 |
| H2AC14 | 1.926891849 | 4.113480487 | 5.86704694 | 1.47E-07 | 1.85E-06 | 6.96857065 |
| DTYMK | 1.083181446 | 7.731490002 | 5.855767845 | 1.54E-07 | 1.93E-06 | 6.924620724 |
| CCN3 | 1.182411776 | 8.127768628 | 5.837347451 | 1.66E-07 | 2.05E-06 | 6.852902244 |
| H2AC15 | 1.834743401 | 8.727390028 | 5.825822122 | 1.74E-07 | 2.13E-06 | 6.808066167 |
| SLC6A4 | 1.022024726 | 6.76942882 | 5.825306794 | 1.74E-07 | 2.14E-06 | 6.806062098 |
| SLCO2A1 | 1.253401758 | 8.283119223 | 5.81028034 | 1.85E-07 | 2.25E-06 | 6.74765067 |
| PIEZO2 | 1.010564518 | 9.35595739 | 5.809603726 | 1.85E-07 | 2.26E-06 | 6.745021664 |
| TTK | 1.532398596 | 5.153295798 | 5.802126278 | 1.91E-07 | 2.31E-06 | 6.71597445 |
| TEX9 | 1.057925794 | 5.387635304 | 5.794004641 | 1.97E-07 | 2.38E-06 | 6.684438674 |
| SLC6A11 | 1.458274565 | 8.343748389 | 5.788666599 | 2.01E-07 | 2.42E-06 | 6.663719312 |
| RCN2 | 1.050520455 | 8.647386066 | 5.776209227 | 2.11E-07 | 2.52E-06 | 6.615391111 |
| DBN1 | 1.049907114 | 8.574812604 | 5.770329471 | 2.16E-07 | 2.58E-06 | 6.592592654 |
| EFNA4 | 1.24240474 | 8.366529751 | 5.766741991 | 2.19E-07 | 2.61E-06 | 6.57868617 |
| H3C12 | 1.993423813 | 7.674183086 | 5.762639543 | 2.23E-07 | 2.65E-06 | 6.562786994 |
| SPINK1 | 3.442134164 | 9.369934796 | 5.759751648 | 2.26E-07 | 2.67E-06 | 6.551597122 |
| DCDC2 | 1.619120515 | 8.196596551 | 5.750185025 | 2.34E-07 | 2.77E-06 | 6.514542248 |
| MDK | 1.162655798 | 10.41527574 | 5.749646741 | 2.35E-07 | 2.77E-06 | 6.5124579 |
| CTSC | 1.085244139 | 10.89968599 | 5.745777344 | 2.39E-07 | 2.81E-06 | 6.497476706 |
| PRR15L | 1.215583328 | 7.257680011 | 5.745679108 | 2.39E-07 | 2.81E-06 | 6.497096408 |
| SERPINH1 | 1.019139619 | 10.35092503 | 5.742899032 | 2.41E-07 | 2.83E-06 | 6.486334908 |
| PLK4 | 1.159711359 | 5.797259038 | 5.741412833 | 2.43E-07 | 2.84E-06 | 6.480582642 |
| DGKK | 1.426002402 | 6.93115851 | 5.738924415 | 2.45E-07 | 2.86E-06 | 6.470952455 |
| SLC39A10 | 1.217838134 | 7.305776396 | 5.730285225 | 2.54E-07 | 2.95E-06 | 6.437529703 |
| DYNC1I1 | 1.560691518 | 7.501810656 | 5.727720937 | 2.56E-07 | 2.96E-06 | 6.427612424 |
| ESCO2 | 1.025365798 | 5.384243546 | 5.725193518 | 2.59E-07 | 2.98E-06 | 6.417839208 |
| FOXQ1 | 1.114754756 | 8.853400475 | 5.722716194 | 2.61E-07 | 3.01E-06 | 6.408261125 |
| LINC00665 | 1.703230281 | 6.888607069 | 5.720667313 | 2.64E-07 | 3.02E-06 | 6.400340593 |
| C1orf112 | 1.006593774 | 7.01306142 | 5.716199485 | 2.68E-07 | 3.07E-06 | 6.383072282 |
| SLC16A12 | 1.505866581 | 6.914130384 | 5.714625901 | 2.70E-07 | 3.08E-06 | 6.37699142 |
| ARHGAP11A | 1.372960066 | 5.757930484 | 5.713877258 | 2.71E-07 | 3.09E-06 | 6.37409861 |
| SNORD50A | 1.283893745 | 8.859926682 | 5.713153917 | 2.71E-07 | 3.10E-06 | 6.371303689 |
| QPCT | 1.088459588 | 6.959721588 | 5.706898419 | 2.78E-07 | 3.16E-06 | 6.347138099 |
| KCNJ5 | 1.205064071 | 7.649147107 | 5.672873336 | 3.18E-07 | 3.56E-06 | 6.215855431 |
| TMC7 | 1.152089904 | 7.210424233 | 5.656138129 | 3.40E-07 | 3.78E-06 | 6.151384106 |
| CDC25A | 1.065815966 | 6.800438381 | 5.652060825 | 3.46E-07 | 3.84E-06 | 6.135686639 |
| PCP4 | 1.797487799 | 8.785231447 | 5.640082795 | 3.62E-07 | 4.01E-06 | 6.089594669 |
| VPS72 | 1.020957624 | 9.509392694 | 5.637444287 | 3.66E-07 | 4.04E-06 | 6.079446208 |
| GAS2L3 | 1.027556732 | 7.56381205 | 5.606982718 | 4.13E-07 | 4.50E-06 | 5.962404273 |
| BARD1 | 1.009222565 | 6.972655936 | 5.596334176 | 4.31E-07 | 4.67E-06 | 5.92154299 |
| TLCD1 | 1.288610678 | 7.681862761 | 5.573323611 | 4.71E-07 | 5.07E-06 | 5.833340971 |
| SGO2 | 1.044678899 | 5.552691443 | 5.567436766 | 4.83E-07 | 5.17E-06 | 5.810797204 |
| PRKD1 | 1.228226193 | 7.921044153 | 5.552662581 | 5.11E-07 | 5.43E-06 | 5.754257499 |
| C19orf48 | 1.193477613 | 8.311632161 | 5.550753487 | 5.15E-07 | 5.47E-06 | 5.746955548 |
| H3C3 | 1.243690784 | 6.995356831 | 5.545808357 | 5.25E-07 | 5.56E-06 | 5.728045573 |
| HTR4 | 1.29545318 | 6.819473994 | 5.537634809 | 5.42E-07 | 5.71E-06 | 5.696803846 |
| ABRAXAS1 | 1.037791678 | 6.855498287 | 5.522814111 | 5.75E-07 | 6.01E-06 | 5.640198145 |
| TK1 | 1.005777759 | 8.072784874 | 5.520476392 | 5.80E-07 | 6.06E-06 | 5.631274667 |
| GJB6 | 1.205774564 | 7.260509276 | 5.511563657 | 6.01E-07 | 6.25E-06 | 5.597266106 |
| H4C2 | 1.419672694 | 8.835672069 | 5.499640453 | 6.30E-07 | 6.52E-06 | 5.551802484 |
| ARHGEF28 | 1.097478349 | 8.160327736 | 5.499122963 | 6.31E-07 | 6.53E-06 | 5.549830113 |
| LIN28B | 2.151232201 | 5.822194894 | 5.489728772 | 6.54E-07 | 6.74E-06 | 5.514036969 |
| OLFML2B | 1.478816198 | 7.76481293 | 5.485824474 | 6.65E-07 | 6.83E-06 | 5.499167833 |
| ITGAE | 1.081303146 | 7.608252789 | 5.469594559 | 7.08E-07 | 7.21E-06 | 5.437400631 |
| PKM | 1.03173229 | 9.766114794 | 5.464135751 | 7.23E-07 | 7.33E-06 | 5.416641296 |
| PGC | 2.882953804 | 9.271129518 | 5.45644434 | 7.45E-07 | 7.53E-06 | 5.387404965 |
| MMP2 | 1.537339866 | 9.535035285 | 5.43790178 | 8.01E-07 | 8.02E-06 | 5.316986372 |
| CPA6 | 1.445832523 | 5.888080845 | 5.414307964 | 8.78E-07 | 8.72E-06 | 5.227518242 |
| CST1 | 3.01899434 | 8.573495199 | 5.391982146 | 9.58E-07 | 9.44E-06 | 5.142997598 |
| MMD | 1.026611794 | 9.13604467 | 5.390052381 | 9.65E-07 | 9.51E-06 | 5.135698341 |
| COL2A1 | 1.154583491 | 8.51328924 | 5.382312279 | 9.95E-07 | 9.75E-06 | 5.106432025 |
| PART1 | 2.033881705 | 5.717165819 | 5.349467472 | 1.13E-06 | 1.09E-05 | 4.982426481 |
| CKAP2L | 1.135155676 | 6.218730557 | 5.334526817 | 1.20E-06 | 1.15E-05 | 4.926118017 |
| TNFSF4 | 1.37549456 | 7.282780706 | 5.330101638 | 1.22E-06 | 1.16E-05 | 4.909452471 |
| KIF15 | 1.122828566 | 5.419852341 | 5.323760983 | 1.25E-06 | 1.19E-05 | 4.885582787 |
| UHRF1 | 1.136325008 | 8.152519739 | 5.322581155 | 1.25E-06 | 1.19E-05 | 4.881142533 |
| HEY1 | 1.141305957 | 7.738823472 | 5.320283363 | 1.27E-06 | 1.20E-05 | 4.872495983 |
| ERP27 | 1.758246481 | 7.833767292 | 5.308560375 | 1.32E-06 | 1.25E-05 | 4.828406069 |
| SLC5A9 | 1.272824506 | 8.272261871 | 5.273157081 | 1.52E-06 | 1.41E-05 | 4.695495208 |
| BRIP1 | 1.095512984 | 6.292566954 | 5.25434571 | 1.63E-06 | 1.50E-05 | 4.625022107 |
| SRARP | 1.175554976 | 7.644221162 | 5.241213966 | 1.72E-06 | 1.57E-05 | 4.575888354 |
| USP27X | 1.056266278 | 8.109477694 | 5.218150849 | 1.88E-06 | 1.70E-05 | 4.489719359 |
| SNORA71C | 1.216100584 | 10.58083145 | 5.208577732 | 1.95E-06 | 1.76E-05 | 4.453998797 |
| SMOC2 | 1.330631992 | 7.469510026 | 5.190884276 | 2.08E-06 | 1.87E-05 | 4.388051283 |
| H2AC20 | 1.10646399 | 9.932533138 | 5.184691029 | 2.13E-06 | 1.91E-05 | 4.364990097 |
| NEK2 | 1.020105928 | 6.557636773 | 5.176444929 | 2.20E-06 | 1.96E-05 | 4.334303063 |
| LINC01549 | 1.621866972 | 5.447939435 | 5.150225044 | 2.44E-06 | 2.15E-05 | 4.236867126 |
| SNORD44 | 1.309898882 | 7.84162479 | 5.14533367 | 2.48E-06 | 2.18E-05 | 4.21871376 |
| MPPED2 | 1.497093097 | 7.495991744 | 5.143239374 | 2.50E-06 | 2.19E-05 | 4.210943467 |
| SNORD30 | 1.113537348 | 7.995096401 | 5.142943502 | 2.50E-06 | 2.19E-05 | 4.20984583 |
| RNASE1 | 1.066256354 | 10.46144144 | 5.133425108 | 2.60E-06 | 2.27E-05 | 4.174548617 |
| SLC1A5 | 1.229878082 | 8.834315988 | 5.130289439 | 2.63E-06 | 2.29E-05 | 4.162926758 |
| SLC2A1 | 1.563974278 | 9.358111316 | 5.110210929 | 2.84E-06 | 2.44E-05 | 4.088582123 |
| H3C1 | 1.727358253 | 7.88150793 | 5.107942658 | 2.86E-06 | 2.46E-05 | 4.080191389 |
| NYNRIN | 1.295176221 | 9.171581237 | 5.095198766 | 3.01E-06 | 2.56E-05 | 4.033079814 |
| SLC22A11 | 1.095875444 | 8.062109117 | 5.076767176 | 3.22E-06 | 2.72E-05 | 3.965033512 |
| DEPDC1 | 1.222154908 | 5.244888808 | 5.050011746 | 3.57E-06 | 2.97E-05 | 3.866451374 |
| MYBL2 | 1.245419756 | 8.102045831 | 5.039749892 | 3.71E-06 | 3.06E-05 | 3.828702499 |
| LPCAT2 | 1.065470543 | 8.509283772 | 5.038298061 | 3.73E-06 | 3.08E-05 | 3.82336462 |
| H2BC10 | 1.472560109 | 5.746532104 | 5.029433712 | 3.86E-06 | 3.17E-05 | 3.790788463 |
| MYCN | 1.752790741 | 8.58215868 | 5.024454348 | 3.93E-06 | 3.22E-05 | 3.772500794 |
| GPD1L | 1.214785538 | 7.132563491 | 5.022103647 | 3.97E-06 | 3.25E-05 | 3.763870225 |
| FGF20 | 1.116251909 | 7.582142022 | 5.018432412 | 4.02E-06 | 3.29E-05 | 3.750394976 |
| BCL11A | 1.245420473 | 7.436137579 | 5.011276062 | 4.13E-06 | 3.36E-05 | 3.724140424 |
| VANGL2 | 1.005586922 | 7.480459214 | 5.001961922 | 4.28E-06 | 3.47E-05 | 3.68999498 |
| COL4A1 | 1.092460999 | 9.197450451 | 4.98351434 | 4.59E-06 | 3.68E-05 | 3.622451786 |
| STRA6 | 1.3608053 | 8.043824353 | 4.971316054 | 4.81E-06 | 3.83E-05 | 3.577852122 |
| EDIL3 | 1.889105841 | 7.614271996 | 4.9575451 | 5.06E-06 | 4.01E-05 | 3.527562745 |
| SLC13A3 | 1.311723638 | 9.050878857 | 4.924763734 | 5.73E-06 | 4.46E-05 | 3.408110005 |
| C12orf75 | 1.08643156 | 8.297093054 | 4.921249853 | 5.81E-06 | 4.51E-05 | 3.395327565 |
| LINC01512 | 1.167145304 | 7.304188723 | 4.917578451 | 5.89E-06 | 4.57E-05 | 3.381976659 |
| MIR15A | 1.046484749 | 6.246451254 | 4.894036279 | 6.43E-06 | 4.94E-05 | 3.296477539 |
| SNORD49A | 1.004142866 | 6.968821984 | 4.883692058 | 6.68E-06 | 5.11E-05 | 3.258971077 |
| ODAM | 2.02151941 | 5.819552763 | 4.879715146 | 6.78E-06 | 5.17E-05 | 3.244561422 |
| EDDM3A | 1.703866605 | 5.970964673 | 4.877177795 | 6.85E-06 | 5.21E-05 | 3.235370675 |
| SNORA5A | 1.091994596 | 8.479744256 | 4.868919362 | 7.06E-06 | 5.34E-05 | 3.205472819 |
| B3GALT2 | 1.662989831 | 6.285589082 | 4.833945995 | 8.05E-06 | 5.98E-05 | 3.079127436 |
| RBP1 | 1.090691404 | 9.527732558 | 4.81807269 | 8.54E-06 | 6.30E-05 | 3.02192761 |
| SNORD81 | 1.120458793 | 8.452665945 | 4.808678102 | 8.85E-06 | 6.51E-05 | 2.988116824 |
| MIR299 | 1.103489775 | 5.289902572 | 4.788334595 | 9.54E-06 | 6.96E-05 | 2.915011155 |
| ZAP70 | 1.230421481 | 7.846400491 | 4.775201766 | 1.00E-05 | 7.26E-05 | 2.867897865 |
| B3GALT1 | 1.436586755 | 5.754869077 | 4.771189407 | 1.02E-05 | 7.36E-05 | 2.853516395 |
| LDOC1 | 1.206832332 | 8.351034578 | 4.766115783 | 1.04E-05 | 7.47E-05 | 2.835339535 |
| SLC6A9 | 1.023158946 | 8.418630273 | 4.76239005 | 1.05E-05 | 7.56E-05 | 2.821997711 |
| FADS2 | 1.565725669 | 11.09920119 | 4.748641074 | 1.11E-05 | 7.90E-05 | 2.772807248 |
| FDPS | 1.017602254 | 9.245243627 | 4.741789511 | 1.13E-05 | 8.05E-05 | 2.748320279 |
| WIF1 | 2.27176879 | 7.286830269 | 4.733240188 | 1.17E-05 | 8.28E-05 | 2.717790235 |
| SEMA3C | 1.396890272 | 6.969690865 | 4.727619124 | 1.19E-05 | 8.43E-05 | 2.69773206 |
| MBNL3 | 1.009580362 | 11.44284296 | 4.726806144 | 1.20E-05 | 8.45E-05 | 2.694832009 |
| DKK3 | 1.107682606 | 9.016645621 | 4.639785443 | 1.65E-05 | 0.000111702 | 2.385866739 |
| IQCH | 1.084036345 | 6.450206725 | 4.639140766 | 1.66E-05 | 0.000111926 | 2.383588711 |
| H4C13 | 1.305165419 | 8.340866306 | 4.59112856 | 1.97E-05 | 0.000130691 | 2.21439148 |
| COL4A2 | 1.01122991 | 9.33484263 | 4.581433184 | 2.04E-05 | 0.000134968 | 2.18033523 |
| SLC38A11 | 1.275107662 | 7.051229443 | 4.578317382 | 2.07E-05 | 0.000136318 | 2.169398543 |
| H4C1 | 1.072139114 | 7.464933341 | 4.57725192 | 2.08E-05 | 0.000136783 | 2.165659585 |
| DACT1 | 1.084040874 | 8.153543513 | 4.570906496 | 2.12E-05 | 0.000139414 | 2.143401409 |
| CPXM1 | 1.147973575 | 8.163440873 | 4.549347313 | 2.30E-05 | 0.00014932 | 2.067898053 |
| ACTG2 | 1.656616796 | 8.793554896 | 4.546688698 | 2.32E-05 | 0.000150625 | 2.058600196 |
| TERC | 1.428897053 | 10.00277638 | 4.534917194 | 2.42E-05 | 0.000156393 | 2.017466687 |
| SNORD78 | 1.165772903 | 7.355162347 | 4.512423872 | 2.63E-05 | 0.000168007 | 1.939024723 |
| TDGF1 | 1.337394146 | 4.279190427 | 4.465949514 | 3.11E-05 | 0.000193906 | 1.777613124 |
| MIR154 | 1.466587291 | 7.043171104 | 4.443382137 | 3.37E-05 | 0.000207969 | 1.699558668 |
| PWAR6 | 1.379217437 | 5.568083092 | 4.409385798 | 3.81E-05 | 0.000231536 | 1.582381185 |
| DUSP9 | 1.52900924 | 9.525435034 | 4.401410253 | 3.92E-05 | 0.000237706 | 1.55496273 |
| MUCL1 | 1.356540728 | 7.369806253 | 4.374261254 | 4.32E-05 | 0.000258837 | 1.461834709 |
| SNORD116-13 | 1.556037184 | 7.786699192 | 4.303735621 | 5.55E-05 | 0.00032182 | 1.221416782 |
| VCAN | 1.678252112 | 9.782831623 | 4.27920354 | 6.05E-05 | 0.000347235 | 1.138305178 |
| MAP1B | 1.002985048 | 8.261224408 | 4.267663899 | 6.30E-05 | 0.000359993 | 1.099303845 |
| LYPD1 | 1.097867273 | 8.001107114 | 4.25811326 | 6.51E-05 | 0.000370991 | 1.067070401 |
| TSPAN18 | 1.437708193 | 9.578727347 | 4.174400508 | 8.74E-05 | 0.000480221 | 0.786327168 |
| SFRP4 | 1.375568768 | 5.614578735 | 4.163598717 | 9.07E-05 | 0.000496615 | 0.750338551 |
| MYEF2 | 1.016300495 | 6.317361027 | 4.157358796 | 9.27E-05 | 0.000505907 | 0.729573812 |
| DLX5 | 1.140947986 | 7.164792234 | 4.140201128 | 9.84E-05 | 0.000531883 | 0.672572493 |
| MYH7B | 1.373015665 | 7.992713735 | 4.096596785 | 0.000114455 | 0.000605783 | 0.528339821 |
| PCDHB14 | 1.055205961 | 6.700006568 | 4.064161205 | 0.000127999 | 0.000669557 | 0.421643812 |
| SHISA6 | 1.02430068 | 7.128033728 | 4.053785371 | 0.000132648 | 0.000691571 | 0.387620702 |
| FMOD | 1.357829044 | 8.764266023 | 4.039985357 | 0.000139085 | 0.00072014 | 0.342451071 |
| FADS1 | 1.028430387 | 10.55994825 | 3.998233051 | 0.000160441 | 0.000813704 | 0.206360996 |
| LRRN1 | 1.154924033 | 5.230238175 | 3.997534727 | 0.000160823 | 0.000814767 | 0.204092191 |
| KCNJ10 | 1.221235997 | 7.903886342 | 3.975014936 | 0.000173647 | 0.000870825 | 0.131057796 |
| SNORD116-11 | 1.237318063 | 7.947855014 | 3.951766513 | 0.000187914 | 0.000933912 | 0.055927904 |
| KRT19 | 1.109252991 | 8.103717254 | 3.944420107 | 0.000192651 | 0.000954434 | 0.032244032 |
| FREM1 | 1.440269769 | 7.976908298 | 3.926294036 | 0.000204837 | 0.001006779 | -0.026074626 |
| MIR382 | 1.241665457 | 5.774161199 | 3.833426951 | 0.0002798 | 0.00131063 | -0.322205887 |
| OLFML3 | 1.086643451 | 9.523513491 | 3.800138488 | 0.000312586 | 0.001442664 | -0.427253972 |
| DKK2 | 1.194262132 | 6.899045149 | 3.798604065 | 0.000314183 | 0.001449677 | -0.432081927 |
| CDH11 | 1.344044727 | 8.520552065 | 3.793556954 | 0.00031949 | 0.00147272 | -0.447953432 |
| SPARCL1 | 1.423824003 | 8.331289146 | 3.793413057 | 0.000319642 | 0.001473063 | -0.448405742 |
| COL1A1 | 1.309318848 | 10.78879275 | 3.701720893 | 0.00043241 | 0.001912217 | -0.734344976 |
| FRZB | 1.261205216 | 7.500117348 | 3.691845647 | 0.000446605 | 0.001961166 | -0.764866585 |
| NRCAM | 1.142640749 | 6.267880628 | 3.67308063 | 0.000474812 | 0.002067664 | -0.822715221 |
| LTBP2 | 1.114302287 | 8.458775263 | 3.660357217 | 0.000494896 | 0.002144206 | -0.861827455 |
| DPP10 | 1.256996582 | 6.392715383 | 3.634278452 | 0.000538618 | 0.002306561 | -0.941711445 |
| CRABP2 | 1.232636903 | 6.960977328 | 3.631004924 | 0.00054436 | 0.00232797 | -0.951711855 |
| CHGB | 1.40901692 | 6.984235417 | 3.594238233 | 0.000612977 | 0.002584997 | -1.063615045 |
| COL1A2 | 1.322444628 | 10.29314172 | 3.56250504 | 0.000678742 | 0.002820625 | -1.159578484 |
| ATP1A2 | 1.224111797 | 7.799491719 | 3.550259429 | 0.000705871 | 0.002917257 | -1.196455229 |
| NQO1 | 1.493769467 | 7.660985519 | 3.533332264 | 0.00074507 | 0.003054446 | -1.247287235 |
| ASB4 | 1.058355446 | 6.640929404 | 3.530484276 | 0.000751865 | 0.003076941 | -1.255823346 |
| SNORA65 | 1.064425939 | 8.385629647 | 3.499584402 | 0.000829471 | 0.00334723 | -1.348133439 |
| CPE | 1.024788677 | 8.529154431 | 3.478517285 | 0.000886674 | 0.003549146 | -1.410747916 |
| FMO1 | 1.084052979 | 5.764601079 | 3.466998191 | 0.000919511 | 0.003664223 | -1.444873429 |
| PDE5A | 1.041790963 | 6.344463578 | 3.443383175 | 0.000990467 | 0.003902359 | -1.514586849 |
| FREM2 | 1.386805036 | 6.371250443 | 3.401249466 | 0.001130106 | 0.004378289 | -1.638139575 |
| SNORD116-29 | 1.000188427 | 8.1799878 | 3.337944897 | 0.001375313 | 0.005177989 | -1.821749551 |
| GFRA3 | 1.405723265 | 7.701086578 | 3.293916092 | 0.001574554 | 0.005833598 | -1.94799743 |
| COL11A2 | 1.062658329 | 8.093815109 | 3.270017535 | 0.001693775 | 0.006215351 | -2.016018048 |
| MCTP1 | 1.130673484 | 7.809149081 | 3.205595616 | 0.002058799 | 0.007362834 | -2.197581376 |
| ERVH48-1 | 1.012192495 | 6.941030572 | 3.166441393 | 0.002315432 | 0.008144489 | -2.306636137 |
| TRH | 1.001990171 | 8.457290607 | 3.138439418 | 0.002517016 | 0.008735525 | -2.384020889 |
| SNORD116-25 | 1.047742273 | 9.237323176 | 3.086179093 | 0.002937801 | 0.009982372 | -2.527073432 |
| CCL18 | 1.077969709 | 7.341658892 | 3.04160188 | 0.003347677 | 0.01115359 | -2.647667673 |
| REG1A | 1.126105268 | 5.845042567 | 2.961519344 | 0.004220495 | 0.013555279 | -2.860960904 |
| SPP1 | 1.294622191 | 9.221577655 | 2.895696502 | 0.005091168 | 0.015901239 | -3.032994721 |
| KRT23 | 1.057740155 | 7.620661858 | 2.811020059 | 0.006455282 | 0.019487659 | -3.24986546 |
| SNORD116-26 | 1.003195229 | 9.723925751 | 2.765917433 | 0.007312129 | 0.021676103 | -3.363308741 |
| HEPACAM | 1.005832403 | 8.043301498 | 2.670826762 | 0.009469888 | 0.026818051 | -3.597676804 |
