# Supplementary material for: A Comprehensive Genomic Analysis Constructs miRNA–mRNA Interaction Network in Hepatoblastoma
Source: Front Cell Dev Biol. 2021 Aug 6;9:655703. doi: 10.3389/fcell.2021.655703 (PMC8377242; doi:10.3389/fcell.2021.655703)
Supplement: Supplementary file 12 [file Table_9.DOCX]

**Table S9. Downregulated DE-mRNAs between HB and normal liver samples from the GSE131329 dataset.**

| **Downregulated DE‐mRNA** | **logFC** | **AveExpr** | ***t*** | **P.Value** | **adj.P.Val** | **B** |
| --- | --- | --- | --- | --- | --- | --- |
| C3P1 | -3.398382531 | 7.330928815 | -17.77162349 | 3.53E-27 | 3.32E-23 | 51.26581097 |
| GLS2 | -3.405991505 | 7.35863353 | -17.18031519 | 2.31E-26 | 1.09E-22 | 49.44602153 |
| SLC22A1 | -4.52652519 | 8.378363025 | -16.91192809 | 5.50E-26 | 2.07E-22 | 48.60557304 |
| HAO2 | -4.440361767 | 7.775769678 | -16.42854179 | 2.68E-25 | 8.40E-22 | 47.06869822 |
| RDH16 | -2.981288501 | 7.707421563 | -16.13150417 | 7.18E-25 | 1.93E-21 | 46.10935275 |
| GBA3 | -3.05543598 | 6.945680279 | -15.07028123 | 2.66E-23 | 4.18E-20 | 42.58735242 |
| SDS | -3.856931259 | 7.804048947 | -14.84333964 | 5.87E-23 | 8.50E-20 | 41.81476946 |
| MGLL | -2.199274421 | 8.233109614 | -14.26206322 | 4.59E-22 | 5.39E-19 | 39.80443398 |
| FETUB | -3.737970751 | 8.219904385 | -13.98607105 | 1.24E-21 | 1.23E-18 | 38.83402849 |
| SRD5A1 | -1.447425501 | 9.003672882 | -13.95250027 | 1.40E-21 | 1.31E-18 | 38.7152934 |
| TTC36 | -1.493400957 | 8.190955936 | -13.93394606 | 1.49E-21 | 1.34E-18 | 38.64960485 |
| CLEC4M | -3.459962181 | 7.042606174 | -13.42318786 | 9.63E-21 | 6.97E-18 | 36.82321411 |
| XDH | -3.450097632 | 7.304399959 | -13.40417017 | 1.03E-20 | 7.20E-18 | 36.75453605 |
| KMO | -3.176596227 | 7.03754358 | -13.21246727 | 2.10E-20 | 1.32E-17 | 36.05955451 |
| CA2 | -2.484648826 | 8.702979409 | -12.99422086 | 4.73E-20 | 2.87E-17 | 35.26240822 |
| CYP2C18 | -3.423144837 | 6.906090164 | -12.93168012 | 5.98E-20 | 3.52E-17 | 35.03281798 |
| GPAT3 | -3.034669889 | 6.979382263 | -12.91139961 | 6.45E-20 | 3.68E-17 | 34.95825666 |
| ASPG | -1.522152344 | 7.97560448 | -12.81857365 | 9.14E-20 | 5.06E-17 | 34.61629223 |
| CYP1A2 | -3.739629424 | 8.221352925 | -12.8100887 | 9.43E-20 | 5.07E-17 | 34.58497788 |
| SRD5A2 | -3.22578442 | 7.400139992 | -12.76389068 | 1.12E-19 | 5.87E-17 | 34.41431532 |
| APOF | -4.456935167 | 7.757625803 | -12.74554458 | 1.20E-19 | 6.12E-17 | 34.34646459 |
| PZP | -2.766601802 | 6.954706026 | -12.6780959 | 1.55E-19 | 7.48E-17 | 34.09663659 |
| BCO2 | -3.765209513 | 7.057636043 | -12.63608486 | 1.82E-19 | 8.26E-17 | 33.94072942 |
| GNE | -1.675237676 | 8.960046282 | -12.63305654 | 1.84E-19 | 8.26E-17 | 33.92948215 |
| GBP7 | -2.565038638 | 6.99978761 | -12.42229586 | 4.09E-19 | 1.67E-16 | 33.14379426 |
| SOCS2 | -1.970345057 | 8.116681461 | -12.30731705 | 6.34E-19 | 2.49E-16 | 32.71275856 |
| CDA | -1.984231808 | 7.542192042 | -12.27765064 | 7.11E-19 | 2.73E-16 | 32.60127009 |
| SNX29P2 | -1.731726258 | 8.084272371 | -12.23127672 | 8.49E-19 | 3.19E-16 | 32.42676928 |
| CYP2B7P | -5.31185684 | 7.974750245 | -12.00428967 | 2.03E-18 | 7.21E-16 | 31.56871666 |
| SPTBN2 | -1.496297505 | 7.404084223 | -11.97957101 | 2.23E-18 | 7.79E-16 | 31.47488531 |
| AVPR1A | -3.297189272 | 6.895543884 | -11.87168918 | 3.39E-18 | 1.12E-15 | 31.06448017 |
| NAT2 | -2.365859652 | 5.50142363 | -11.83743709 | 3.87E-18 | 1.25E-15 | 30.93387697 |
| DHODH | -1.832553263 | 8.106134932 | -11.81143814 | 4.29E-18 | 1.34E-15 | 30.83464665 |
| OGDHL | -2.58063259 | 7.933441978 | -11.79651233 | 4.54E-18 | 1.38E-15 | 30.77764176 |
| ERN1 | -1.090284846 | 8.871958411 | -11.78982866 | 4.66E-18 | 1.39E-15 | 30.75210653 |
| GAREM1 | -1.753215006 | 8.134399705 | -11.71442097 | 6.25E-18 | 1.84E-15 | 30.4636301 |
| LPA | -2.840718546 | 7.916505631 | -11.61593664 | 9.17E-18 | 2.66E-15 | 30.08583254 |
| TRIB1 | -2.096586091 | 10.18031492 | -11.60701382 | 9.50E-18 | 2.71E-15 | 30.05154573 |
| GCH1 | -1.918002276 | 7.963100892 | -11.55312736 | 1.17E-17 | 3.29E-15 | 29.84427844 |
| FCN2 | -1.85193 | 8.271243434 | -11.48398949 | 1.54E-17 | 4.13E-15 | 29.57784075 |
| SLC27A5 | -2.360525487 | 8.913874716 | -11.42119033 | 1.97E-17 | 5.14E-15 | 29.33533936 |
| AKR7L | -2.091157244 | 6.841708181 | -11.36759477 | 2.43E-17 | 6.18E-15 | 29.12801094 |
| HGFAC | -1.963614827 | 8.614663789 | -11.33780173 | 2.73E-17 | 6.85E-15 | 29.01261462 |
| BBOX1 | -2.066137134 | 4.676457237 | -11.28686989 | 3.34E-17 | 8.26E-15 | 28.81510299 |
| SLC1A1 | -3.838464426 | 7.297951719 | -11.14730048 | 5.79E-17 | 1.38E-14 | 28.27232771 |
| KIAA1671 | -1.130267258 | 8.962769206 | -11.09095165 | 7.24E-17 | 1.68E-14 | 28.05256304 |
| CFHR4 | -3.570192325 | 7.460890303 | -11.07121205 | 7.83E-17 | 1.78E-14 | 27.97549255 |
| GSTA3 | -1.442910779 | 5.695423657 | -11.06929637 | 7.89E-17 | 1.78E-14 | 27.96801072 |
| IL13RA2 | -1.400898644 | 3.974574585 | -11.06102824 | 8.15E-17 | 1.80E-14 | 27.93571422 |
| CLEC4G | -2.274703125 | 8.859904222 | -11.03634252 | 8.99E-17 | 1.95E-14 | 27.83924284 |
| ETNPPL | -2.248123825 | 6.393741949 | -11.01881784 | 9.64E-17 | 2.06E-14 | 27.77071544 |
| KCND3 | -1.989031002 | 6.684819228 | -10.95897244 | 1.22E-16 | 2.56E-14 | 27.53644321 |
| GPLD1 | -2.124134436 | 7.651503235 | -10.95003023 | 1.27E-16 | 2.62E-14 | 27.50140389 |
| SPATA6L | -1.782454646 | 6.743186677 | -10.93385619 | 1.35E-16 | 2.76E-14 | 27.43800498 |
| RUNDC3B | -2.106201061 | 6.584236857 | -10.87006239 | 1.74E-16 | 3.49E-14 | 27.18766775 |
| MAP7 | -1.833283063 | 7.456651357 | -10.81452274 | 2.17E-16 | 4.26E-14 | 26.96936214 |
| RIDA | -2.842267835 | 9.543369713 | -10.7212414 | 3.16E-16 | 6.00E-14 | 26.60196598 |
| CFHR3 | -4.275740944 | 6.736752414 | -10.70197743 | 3.41E-16 | 6.42E-14 | 26.52597861 |
| CTH | -2.512634128 | 8.25480776 | -10.66903656 | 3.89E-16 | 7.25E-14 | 26.39595222 |
| MARCO | -1.600463968 | 7.432256641 | -10.64698348 | 4.25E-16 | 7.82E-14 | 26.30883965 |
| DTX1 | -1.952613395 | 8.153572666 | -10.64555509 | 4.28E-16 | 7.82E-14 | 26.30319556 |
| GPT | -1.36119663 | 7.897450503 | -10.63597115 | 4.44E-16 | 8.04E-14 | 26.26532061 |
| VNN3 | -2.806545924 | 7.119095954 | -10.63158519 | 4.52E-16 | 8.11E-14 | 26.24798447 |
| PPP1R1A | -2.155342046 | 9.009812041 | -10.57114434 | 5.77E-16 | 1.00E-13 | 26.00888181 |
| GNMT | -3.048365838 | 8.244876963 | -10.53621537 | 6.64E-16 | 1.15E-13 | 25.8705335 |
| GNAO1 | -2.034392117 | 6.950393366 | -10.51140345 | 7.33E-16 | 1.24E-13 | 25.77218224 |
| RND1 | -2.250314184 | 8.149381425 | -10.50416985 | 7.55E-16 | 1.27E-13 | 25.7434975 |
| THRSP | -2.965092238 | 7.256144861 | -10.46662395 | 8.78E-16 | 1.42E-13 | 25.59452566 |
| ADRA1A | -2.856067991 | 7.232163712 | -10.46036828 | 9.01E-16 | 1.44E-13 | 25.56969118 |
| CYP2B6 | -5.21307 | 9.083542802 | -10.43274742 | 1.01E-15 | 1.59E-13 | 25.45999246 |
| UPP1 | -1.007056045 | 7.238972629 | -10.34330652 | 1.44E-15 | 2.17E-13 | 25.10425594 |
| CLEC1B | -2.434980154 | 6.24503938 | -10.33763207 | 1.48E-15 | 2.21E-13 | 25.08166053 |
| LINC01554 | -1.46735209 | 6.724724206 | -10.28498798 | 1.83E-15 | 2.65E-13 | 24.87188691 |
| NNMT | -3.427392656 | 9.341375391 | -10.20369874 | 2.54E-15 | 3.65E-13 | 24.54745417 |
| ANXA10 | -1.490481315 | 5.397476852 | -10.17606905 | 2.84E-15 | 4.05E-13 | 24.43704124 |
| NRG1 | -1.813585258 | 6.220561212 | -10.163231 | 2.99E-15 | 4.24E-13 | 24.38571428 |
| HAL | -3.992893673 | 8.770964103 | -10.12217198 | 3.54E-15 | 4.93E-13 | 24.22145787 |
| LRG1 | -2.311391451 | 9.42662443 | -10.0943539 | 3.96E-15 | 5.47E-13 | 24.11008495 |
| ETS2 | -1.572303517 | 10.570637 | -10.09309165 | 3.98E-15 | 5.47E-13 | 24.10502971 |
| SERPINB8 | -2.271665398 | 7.175869075 | -10.07800879 | 4.23E-15 | 5.70E-13 | 24.04461295 |
| GLYATL1 | -2.671822914 | 8.930597359 | -10.02511958 | 5.25E-15 | 7.01E-13 | 23.83259725 |
| ESR1 | -1.512812148 | 6.879778838 | -10.0181643 | 5.40E-15 | 7.16E-13 | 23.80469745 |
| BMPER | -1.44450995 | 6.922167531 | -10.00456588 | 5.71E-15 | 7.51E-13 | 23.75013773 |
| TRIM55 | -1.799537691 | 6.362613305 | -9.894411425 | 8.94E-15 | 1.15E-12 | 23.30758787 |
| AKR1D1 | -3.190244136 | 7.675199093 | -9.894109206 | 8.95E-15 | 1.15E-12 | 23.30637228 |
| SHFL | -1.063652723 | 9.126303721 | -9.818613985 | 1.22E-14 | 1.52E-12 | 23.00247635 |
| ACACB | -1.825270714 | 8.773660278 | -9.808800266 | 1.27E-14 | 1.57E-12 | 22.96293822 |
| CXCL2 | -2.517157783 | 8.307501078 | -9.801483109 | 1.31E-14 | 1.59E-12 | 22.93345331 |
| RNF152 | -2.17321749 | 8.504230187 | -9.797458192 | 1.33E-14 | 1.60E-12 | 22.91723284 |
| HSD17B13 | -4.827027471 | 7.810676243 | -9.793691038 | 1.35E-14 | 1.61E-12 | 22.90204996 |
| PRKAG2 | -1.428360897 | 7.835703821 | -9.751608739 | 1.60E-14 | 1.87E-12 | 22.73236721 |
| MOGAT2 | -1.926291463 | 7.168629516 | -9.742814625 | 1.66E-14 | 1.91E-12 | 22.69689012 |
| AKR1C8P | -2.124832805 | 6.697426341 | -9.720005715 | 1.82E-14 | 2.08E-12 | 22.60484641 |
| GSTA2 | -3.052961651 | 6.225055646 | -9.691522638 | 2.05E-14 | 2.32E-12 | 22.48984806 |
| RBM47 | -1.452025671 | 8.918690457 | -9.678617859 | 2.16E-14 | 2.44E-12 | 22.43772534 |
| USP2 | -1.448048013 | 7.440847994 | -9.67281339 | 2.21E-14 | 2.48E-12 | 22.41427678 |
| HSD11B1 | -3.936981926 | 7.774957147 | -9.648605351 | 2.44E-14 | 2.71E-12 | 22.31645486 |
| GOT1 | -1.788529732 | 10.89413431 | -9.648254007 | 2.45E-14 | 2.71E-12 | 22.31503479 |
| RCAN1 | -1.394531585 | 8.719436536 | -9.629465069 | 2.64E-14 | 2.91E-12 | 22.2390798 |
| IYD | -1.44871557 | 6.881782978 | -9.578676579 | 3.26E-14 | 3.56E-12 | 22.03363376 |
| CYP2C19 | -4.255192741 | 8.516703432 | -9.559536859 | 3.52E-14 | 3.81E-12 | 21.95616195 |
| SULT1A2 | -1.699002158 | 8.382899489 | -9.522921571 | 4.10E-14 | 4.33E-12 | 21.80788065 |
| SMIM14 | -1.476454958 | 9.640672756 | -9.49215182 | 4.65E-14 | 4.89E-12 | 21.68319835 |
| ACADL | -2.617601979 | 5.485935577 | -9.483495164 | 4.82E-14 | 5.04E-12 | 21.64810869 |
| NFKBIZ | -1.588024036 | 8.302908065 | -9.48027572 | 4.88E-14 | 5.07E-12 | 21.63505737 |
| SLC7A2 | -1.586271354 | 11.08599073 | -9.468732953 | 5.12E-14 | 5.29E-12 | 21.58825821 |
| GDA | -3.287678503 | 6.928994211 | -9.446495637 | 5.61E-14 | 5.77E-12 | 21.49807303 |
| NAMPT | -2.669674254 | 9.764287481 | -9.396483046 | 6.89E-14 | 6.95E-12 | 21.2951205 |
| ACMSD | -2.226820401 | 7.568128832 | -9.380658593 | 7.35E-14 | 7.36E-12 | 21.23086968 |
| MMUT | -1.713249597 | 9.030951472 | -9.373958344 | 7.56E-14 | 7.53E-12 | 21.20366021 |
| SLC46A3 | -2.196650161 | 7.065889265 | -9.368268947 | 7.74E-14 | 7.67E-12 | 21.18055346 |
| SLC25A18 | -2.337267756 | 8.754806654 | -9.366918415 | 7.78E-14 | 7.67E-12 | 21.17506814 |
| C8G | -2.142217612 | 8.982177526 | -9.363575413 | 7.89E-14 | 7.73E-12 | 21.1614897 |
| HABP2 | -3.445561977 | 9.460568324 | -9.350098093 | 8.34E-14 | 8.09E-12 | 21.10674082 |
| ZC3H12A | -1.238408276 | 8.173769133 | -9.331441275 | 9.01E-14 | 8.65E-12 | 21.03093199 |
| GLT1D1 | -2.454462638 | 7.880858265 | -9.328148433 | 9.13E-14 | 8.72E-12 | 21.01754978 |
| INSIG1 | -2.034934054 | 9.566924733 | -9.313929916 | 9.68E-14 | 9.20E-12 | 20.95975737 |
| NFIL3 | -1.868551332 | 8.82631181 | -9.301516584 | 1.02E-13 | 9.61E-12 | 20.90929188 |
| LIPG | -2.104054018 | 8.579302507 | -9.301138052 | 1.02E-13 | 9.61E-12 | 20.90775284 |
| ARHGEF26 | -2.747280888 | 7.258765365 | -9.287146152 | 1.08E-13 | 1.00E-11 | 20.85085793 |
| SLC10A1 | -4.135818515 | 8.958838795 | -9.281203185 | 1.11E-13 | 1.02E-11 | 20.82668852 |
| MBL2 | -2.5553105 | 9.72925086 | -9.230329977 | 1.37E-13 | 1.26E-11 | 20.61970442 |
| CPEB3 | -1.244688427 | 7.561433683 | -9.227633083 | 1.38E-13 | 1.26E-11 | 20.60872742 |
| SLITRK3 | -2.198894748 | 5.268134737 | -9.196939253 | 1.57E-13 | 1.41E-11 | 20.48376591 |
| CYP2C8 | -5.706144294 | 8.587590889 | -9.174340014 | 1.72E-13 | 1.54E-11 | 20.39172421 |
| CFHR5 | -3.662424653 | 6.704750073 | -9.168505235 | 1.77E-13 | 1.57E-11 | 20.36795569 |
| F9 | -4.700269356 | 8.069449268 | -9.163718698 | 1.80E-13 | 1.60E-11 | 20.34845583 |
| BLNK | -1.893794127 | 8.201646122 | -9.159343675 | 1.83E-13 | 1.62E-11 | 20.33063129 |
| CDC37L1 | -1.925179126 | 7.836249965 | -9.144799573 | 1.95E-13 | 1.71E-11 | 20.27136857 |
| NR3C2 | -1.298438233 | 6.640782014 | -9.125823038 | 2.11E-13 | 1.83E-11 | 20.19402729 |
| HAAO | -1.481937735 | 10.09291882 | -9.10013557 | 2.34E-13 | 2.00E-11 | 20.0893032 |
| AADAT | -1.656049438 | 8.337144341 | -9.096650168 | 2.38E-13 | 2.02E-11 | 20.07509095 |
| ACAA2 | -1.204138313 | 9.232357178 | -9.090712448 | 2.43E-13 | 2.05E-11 | 20.05087752 |
| AR | -2.931813756 | 8.074065855 | -9.080036644 | 2.54E-13 | 2.14E-11 | 20.0073379 |
| PGLYRP2 | -2.517688556 | 7.688934449 | -9.058617619 | 2.78E-13 | 2.31E-11 | 19.91996552 |
| HSD17B6 | -4.445812352 | 8.551458529 | -9.046108405 | 2.93E-13 | 2.41E-11 | 19.86892691 |
| REPS2 | -1.590833455 | 7.774367227 | -9.04503741 | 2.94E-13 | 2.41E-11 | 19.86455679 |
| RETREG1 | -2.010671638 | 6.92478274 | -9.018025877 | 3.29E-13 | 2.68E-11 | 19.75431864 |
| SUCLG2 | -1.437744289 | 9.101708052 | -9.014017013 | 3.34E-13 | 2.71E-11 | 19.73795471 |
| CYP26A1 | -1.394587692 | 6.130039899 | -9.006934546 | 3.44E-13 | 2.77E-11 | 19.70904256 |
| MT1IP | -1.211643293 | 7.339152953 | -8.996009889 | 3.60E-13 | 2.89E-11 | 19.66444087 |
| RAB27A | -1.555398295 | 7.565365305 | -8.982940239 | 3.80E-13 | 3.02E-11 | 19.61107418 |
| SLC51A | -2.32255375 | 8.5732068 | -8.980956835 | 3.84E-13 | 3.03E-11 | 19.60297471 |
| NDST3 | -1.438570601 | 5.214037983 | -8.949575623 | 4.37E-13 | 3.41E-11 | 19.47480051 |
| OIT3 | -2.71263922 | 8.551880677 | -8.938131637 | 4.58E-13 | 3.56E-11 | 19.42804678 |
| ABLIM3 | -2.013532519 | 8.949176106 | -8.935040023 | 4.64E-13 | 3.58E-11 | 19.41541513 |
| TSKU | -1.756693717 | 10.45423898 | -8.912860334 | 5.09E-13 | 3.88E-11 | 19.32478084 |
| NSUN6 | -2.409813594 | 8.097453363 | -8.898480539 | 5.40E-13 | 4.08E-11 | 19.26600779 |
| GADD45B | -1.703341133 | 8.906186606 | -8.876066612 | 5.93E-13 | 4.44E-11 | 19.17437927 |
| DHTKD1 | -1.462717615 | 9.726798356 | -8.864104422 | 6.23E-13 | 4.65E-11 | 19.12546861 |
| MT1H | -2.541172549 | 7.944232181 | -8.841059467 | 6.85E-13 | 5.08E-11 | 19.03122576 |
| MAP3K14 | -1.034534795 | 7.326746692 | -8.83673216 | 6.98E-13 | 5.13E-11 | 19.01352664 |
| SLC31A1 | -1.222901379 | 10.57985797 | -8.834163578 | 7.05E-13 | 5.14E-11 | 19.00302051 |
| MTHFD2L | -1.240566188 | 6.417730537 | -8.81935006 | 7.50E-13 | 5.43E-11 | 18.94242426 |
| SLC22A10 | -3.486179108 | 8.231761021 | -8.812845002 | 7.70E-13 | 5.55E-11 | 18.9158118 |
| PTS | -1.544108372 | 7.228354117 | -8.810181451 | 7.79E-13 | 5.59E-11 | 18.9049146 |
| MFSD2A | -2.429172746 | 8.173463294 | -8.790418081 | 8.45E-13 | 5.99E-11 | 18.82404923 |
| BMP10 | -2.398134344 | 6.16240405 | -8.782877163 | 8.72E-13 | 6.13E-11 | 18.79319013 |
| GSTA1 | -3.3755703 | 8.058127829 | -8.773915908 | 9.05E-13 | 6.33E-11 | 18.7565158 |
| UPB1 | -2.654455882 | 8.258290954 | -8.759318648 | 9.62E-13 | 6.68E-11 | 18.69676925 |
| CYP2D6 | -2.512511211 | 9.931720712 | -8.751838729 | 9.92E-13 | 6.77E-11 | 18.66615082 |
| SLC15A1 | -1.928631091 | 7.325001461 | -8.751688582 | 9.93E-13 | 6.77E-11 | 18.66553619 |
| RASGEF1B | -1.994161552 | 8.64874526 | -8.749943566 | 1.00E-12 | 6.77E-11 | 18.6583928 |
| N4BP2L1 | -1.489787747 | 7.526192298 | -8.741408819 | 1.04E-12 | 6.92E-11 | 18.62345335 |
| PVALB | -1.172127356 | 5.824189925 | -8.73741503 | 1.05E-12 | 7.01E-11 | 18.60710269 |
| TMPRSS2 | -1.919014975 | 8.114854394 | -8.735544132 | 1.06E-12 | 7.01E-11 | 18.59944299 |
| LONP2 | -1.256533691 | 10.66511714 | -8.73463738 | 1.07E-12 | 7.01E-11 | 18.59573059 |
| PKD1L3 | -1.107830765 | 5.113877477 | -8.730203797 | 1.09E-12 | 7.12E-11 | 18.57757827 |
| ALDH8A1 | -2.446281697 | 7.966689095 | -8.72795384 | 1.10E-12 | 7.16E-11 | 18.56836605 |
| SEC14L2 | -1.955249516 | 8.275932871 | -8.707646108 | 1.19E-12 | 7.71E-11 | 18.48520982 |
| HAMP | -2.717922486 | 8.460728685 | -8.702836956 | 1.22E-12 | 7.84E-11 | 18.46551513 |
| UGP2 | -2.070842628 | 9.032342447 | -8.699393404 | 1.23E-12 | 7.90E-11 | 18.45141242 |
| C9 | -4.85339197 | 8.655945109 | -8.63669498 | 1.60E-12 | 1.00E-10 | 18.19456729 |
| MAN1C1 | -1.164397328 | 8.136452612 | -8.61694906 | 1.74E-12 | 1.08E-10 | 18.11365147 |
| CLRN3 | -1.487167927 | 5.575952256 | -8.607539494 | 1.81E-12 | 1.12E-10 | 18.07508826 |
| LONRF3 | -2.014690585 | 8.464297976 | -8.594236272 | 1.91E-12 | 1.17E-10 | 18.02056317 |
| TDO2 | -4.248244021 | 8.537307179 | -8.572150157 | 2.09E-12 | 1.27E-10 | 17.93002876 |
| KLF9 | -1.284889818 | 8.744681598 | -8.56605754 | 2.15E-12 | 1.30E-10 | 17.90505174 |
| NUDT16P1 | -1.165461248 | 7.832591568 | -8.562326897 | 2.18E-12 | 1.32E-10 | 17.88975725 |
| DUSP5 | -1.327925695 | 8.628136547 | -8.559874984 | 2.20E-12 | 1.33E-10 | 17.87970496 |
| HERC5 | -1.316584863 | 6.165440645 | -8.546808854 | 2.33E-12 | 1.40E-10 | 17.82613396 |
| LPIN2 | -1.514179546 | 9.649788412 | -8.537347087 | 2.42E-12 | 1.43E-10 | 17.78733796 |
| GSTZ1 | -1.046128667 | 7.643332147 | -8.516066919 | 2.65E-12 | 1.55E-10 | 17.70007455 |
| C6 | -3.234765964 | 9.053837505 | -8.502205979 | 2.80E-12 | 1.63E-10 | 17.64322896 |
| GLYAT | -3.966948151 | 7.764378204 | -8.488186916 | 2.97E-12 | 1.72E-10 | 17.58573015 |
| CNDP1 | -1.732522264 | 6.007380017 | -8.47375459 | 3.15E-12 | 1.80E-10 | 17.52653151 |
| NNT | -1.404728464 | 9.679554475 | -8.469924969 | 3.21E-12 | 1.82E-10 | 17.51082234 |
| UGT2B7 | -2.923980733 | 7.100104758 | -8.459194017 | 3.35E-12 | 1.89E-10 | 17.46680204 |
| HOMER2 | -1.205995158 | 8.434324246 | -8.454714187 | 3.42E-12 | 1.92E-10 | 17.44842422 |
| IL4R | -1.216104221 | 9.305524828 | -8.451242217 | 3.46E-12 | 1.93E-10 | 17.43418069 |
| ASL | -1.250058713 | 9.647095587 | -8.420777097 | 3.93E-12 | 2.16E-10 | 17.30918872 |
| CLTRN | -1.497189887 | 6.929828907 | -8.415706313 | 4.02E-12 | 2.20E-10 | 17.28838254 |
| NTN4 | -1.355893709 | 8.140889916 | -8.411331633 | 4.09E-12 | 2.24E-10 | 17.27043218 |
| PAPSS2 | -1.70436582 | 9.428278206 | -8.407915889 | 4.15E-12 | 2.26E-10 | 17.25641631 |
| CYP2D7 | -2.496655954 | 9.650559908 | -8.400293034 | 4.28E-12 | 2.32E-10 | 17.22513654 |
| CCDC71L | -1.07163149 | 7.79811282 | -8.394840288 | 4.38E-12 | 2.36E-10 | 17.20276098 |
| C8B | -2.077097608 | 9.986073268 | -8.389395485 | 4.48E-12 | 2.40E-10 | 17.18041746 |
| TPD52L1 | -1.148438404 | 8.124898053 | -8.366707128 | 4.93E-12 | 2.61E-10 | 17.08730701 |
| CTSL | -1.478552996 | 9.921985321 | -8.363419718 | 5.00E-12 | 2.64E-10 | 17.07381513 |
| SLED1 | -1.404972873 | 6.719023808 | -8.360924567 | 5.05E-12 | 2.66E-10 | 17.06357464 |
| AKR7A3 | -1.00500751 | 8.356196327 | -8.353280267 | 5.21E-12 | 2.74E-10 | 17.03220063 |
| GREM2 | -1.725565067 | 7.066855481 | -8.34522722 | 5.39E-12 | 2.82E-10 | 16.99914802 |
| LPAL2 | -1.715221657 | 7.400706422 | -8.341796178 | 5.47E-12 | 2.85E-10 | 16.98506548 |
| NDUFV2 | -1.028925214 | 9.680970487 | -8.339927263 | 5.51E-12 | 2.86E-10 | 16.97739454 |
| RNF125 | -1.868996 | 7.737817985 | -8.316174104 | 6.08E-12 | 3.15E-10 | 16.87989552 |
| MT1JP | -1.771667989 | 8.131581407 | -8.280104977 | 7.07E-12 | 3.58E-10 | 16.73182903 |
| TMOD1 | -1.971377039 | 8.317803299 | -8.274241561 | 7.24E-12 | 3.65E-10 | 16.70775779 |
| ANGPTL6 | -1.032328739 | 8.058768677 | -8.272085487 | 7.31E-12 | 3.67E-10 | 16.6989063 |
| ADGRG7 | -2.070054347 | 6.563023522 | -8.267103937 | 7.46E-12 | 3.73E-10 | 16.67845499 |
| BACH2 | -1.127645633 | 7.064960694 | -8.24818766 | 8.07E-12 | 4.00E-10 | 16.60079354 |
| MT1B | -1.06530547 | 7.36260386 | -8.24354551 | 8.23E-12 | 4.07E-10 | 16.5817345 |
| STEAP3 | -1.381724891 | 8.900065507 | -8.242874956 | 8.26E-12 | 4.07E-10 | 16.57898142 |
| TUBE1 | -1.315994108 | 7.113252095 | -8.235364746 | 8.52E-12 | 4.17E-10 | 16.54814665 |
| STAB2 | -2.872476541 | 7.518841164 | -8.218039547 | 9.16E-12 | 4.44E-10 | 16.47701253 |
| ADHFE1 | -1.649716676 | 8.878054275 | -8.207649015 | 9.56E-12 | 4.61E-10 | 16.43434978 |
| DECR1 | -1.138969217 | 9.123442382 | -8.190886677 | 1.03E-11 | 4.91E-10 | 16.36552329 |
| KLF6 | -1.380225392 | 9.660134116 | -8.182546935 | 1.06E-11 | 5.05E-10 | 16.3312795 |
| INPP1 | -1.742251611 | 7.409253351 | -8.176751963 | 1.09E-11 | 5.16E-10 | 16.30748456 |
| PPARGC1A | -2.632739458 | 8.251648444 | -8.167316976 | 1.13E-11 | 5.31E-10 | 16.26874287 |
| TJP2 | -1.214852791 | 9.31616494 | -8.163160212 | 1.15E-11 | 5.39E-10 | 16.25167435 |
| PON3 | -1.945249458 | 10.71779958 | -8.149281161 | 1.22E-11 | 5.70E-10 | 16.19468362 |
| STARD5 | -1.115181749 | 7.851851161 | -8.140135003 | 1.27E-11 | 5.89E-10 | 16.15712692 |
| MCC | -1.139661499 | 7.792236137 | -8.133869322 | 1.30E-11 | 5.97E-10 | 16.13139814 |
| APBB1IP | -1.66535566 | 7.894565152 | -8.133808466 | 1.30E-11 | 5.97E-10 | 16.13114825 |
| CSRNP1 | -1.487534155 | 8.110178207 | -8.096473994 | 1.52E-11 | 6.86E-10 | 15.97784022 |
| GYS2 | -3.696935452 | 8.185607433 | -8.08824267 | 1.57E-11 | 7.05E-10 | 15.94403949 |
| SLC41A2 | -2.748419304 | 8.062900894 | -8.077078272 | 1.65E-11 | 7.37E-10 | 15.8981946 |
| ZFP36 | -1.888805514 | 9.628407344 | -8.073968698 | 1.67E-11 | 7.43E-10 | 15.88542563 |
| CFP | -1.430168319 | 8.123240596 | -8.072285319 | 1.68E-11 | 7.46E-10 | 15.87851311 |
| RND3 | -2.072477176 | 8.416162489 | -8.063027774 | 1.75E-11 | 7.72E-10 | 15.84049861 |
| ABHD5 | -1.116857512 | 8.392475548 | -8.061297569 | 1.76E-11 | 7.76E-10 | 15.83339385 |
| SCP2 | -1.864827286 | 9.928619324 | -8.057152214 | 1.79E-11 | 7.86E-10 | 15.81637174 |
| CES4A | -1.05651627 | 7.376020219 | -8.04367822 | 1.89E-11 | 8.23E-10 | 15.7610437 |
| ACSL1 | -2.183717327 | 10.85097945 | -8.041755912 | 1.91E-11 | 8.24E-10 | 15.7531502 |
| CETP | -1.909450823 | 7.921412659 | -8.04169292 | 1.91E-11 | 8.24E-10 | 15.75289154 |
| PCK2 | -1.664161793 | 9.858071168 | -8.040889508 | 1.92E-11 | 8.25E-10 | 15.74959253 |
| PCCB | -1.037705799 | 9.718262269 | -8.03715697 | 1.95E-11 | 8.33E-10 | 15.73426581 |
| SLPI | -2.401008443 | 7.82533334 | -8.000086652 | 2.27E-11 | 9.61E-10 | 15.58204949 |
| UROC1 | -2.025699305 | 8.057700457 | -7.998181535 | 2.29E-11 | 9.64E-10 | 15.574227 |
| USP38 | -1.160739259 | 8.360903986 | -7.997585996 | 2.30E-11 | 9.64E-10 | 15.5717817 |
| SHBG | -1.842919799 | 8.094531874 | -7.995904573 | 2.31E-11 | 9.67E-10 | 15.56487773 |
| MFAP3L | -1.93215008 | 7.031093935 | -7.985519088 | 2.41E-11 | 1.00E-09 | 15.52223508 |
| TTC39C | -1.456298041 | 9.09661295 | -7.977958694 | 2.49E-11 | 1.03E-09 | 15.4911927 |
| HJV | -2.350542187 | 8.052634856 | -7.972702965 | 2.55E-11 | 1.05E-09 | 15.46961336 |
| FBP1 | -2.515512402 | 10.32286941 | -7.9702893 | 2.57E-11 | 1.06E-09 | 15.45970324 |
| CYP4Z1 | -1.331711117 | 5.855375949 | -7.958320108 | 2.70E-11 | 1.10E-09 | 15.4105604 |
| GHR | -2.810463286 | 8.884185305 | -7.954999708 | 2.74E-11 | 1.12E-09 | 15.3969278 |
| CECR2 | -1.297373705 | 7.064847274 | -7.943238983 | 2.88E-11 | 1.17E-09 | 15.34864254 |
| C1RL | -1.352932795 | 9.605145284 | -7.941582821 | 2.90E-11 | 1.17E-09 | 15.34184305 |
| CD14 | -1.746879461 | 10.87590751 | -7.932691068 | 3.01E-11 | 1.21E-09 | 15.30533783 |
| SOD2 | -2.050925931 | 9.994931543 | -7.930841521 | 3.03E-11 | 1.21E-09 | 15.29774459 |
| DNASE1L3 | -2.209170868 | 7.579255537 | -7.917980058 | 3.20E-11 | 1.27E-09 | 15.24494348 |
| CRHBP | -2.890861435 | 8.591263505 | -7.891134889 | 3.58E-11 | 1.41E-09 | 15.13474055 |
| PFKFB1 | -2.511048775 | 8.244079694 | -7.887911877 | 3.63E-11 | 1.42E-09 | 15.12151031 |
| SERPING1 | -1.108424422 | 12.56739335 | -7.885245635 | 3.67E-11 | 1.44E-09 | 15.11056568 |
| ZG16 | -1.236555383 | 6.960246722 | -7.88309406 | 3.70E-11 | 1.45E-09 | 15.10173377 |
| CYP39A1 | -2.68078182 | 6.85742178 | -7.87100399 | 3.89E-11 | 1.50E-09 | 15.05210702 |
| AGL | -1.524248689 | 7.751712398 | -7.866331821 | 3.97E-11 | 1.53E-09 | 15.03292951 |
| PPP1R3B | -2.244838382 | 8.388381716 | -7.85284081 | 4.20E-11 | 1.61E-09 | 14.97755587 |
| MT1X | -2.08095184 | 11.46700547 | -7.845926438 | 4.32E-11 | 1.65E-09 | 14.94917709 |
| FAM99A | -1.764600272 | 7.619492128 | -7.843332821 | 4.37E-11 | 1.66E-09 | 14.93853227 |
| HRG | -3.720389775 | 10.10476516 | -7.835560873 | 4.51E-11 | 1.70E-09 | 14.90663504 |
| ACSM5 | -2.344379007 | 8.277775994 | -7.825505919 | 4.71E-11 | 1.77E-09 | 14.8653696 |
| SERPINA10 | -1.274932328 | 9.483657394 | -7.815080233 | 4.91E-11 | 1.84E-09 | 14.82258465 |
| MT1HL1 | -1.134550789 | 8.069954282 | -7.810614988 | 5.01E-11 | 1.87E-09 | 14.8042608 |
| ADK | -1.24915419 | 8.615579051 | -7.8039401 | 5.15E-11 | 1.91E-09 | 14.77687005 |
| SERPINE1 | -2.659203062 | 10.0792853 | -7.803894132 | 5.15E-11 | 1.91E-09 | 14.77668142 |
| VIPR1 | -1.43482969 | 7.854047952 | -7.797838158 | 5.28E-11 | 1.95E-09 | 14.75183119 |
| GCLM | -1.303804623 | 7.149981049 | -7.797578049 | 5.29E-11 | 1.95E-09 | 14.75076387 |
| LRAT | -1.288304744 | 6.275908673 | -7.794869783 | 5.35E-11 | 1.97E-09 | 14.73965096 |
| GPD1 | -1.840326924 | 8.352169047 | -7.789868058 | 5.46E-11 | 2.00E-09 | 14.71912763 |
| PTPN3 | -1.769749699 | 8.778786113 | -7.783434752 | 5.61E-11 | 2.04E-09 | 14.69273093 |
| ABHD2 | -1.439381424 | 10.20962336 | -7.754730322 | 6.32E-11 | 2.24E-09 | 14.57496396 |
| PDLIM5 | -1.123285924 | 9.410625557 | -7.748550396 | 6.48E-11 | 2.29E-09 | 14.54961177 |
| SLC25A15 | -1.410887479 | 8.633647717 | -7.739251346 | 6.74E-11 | 2.37E-09 | 14.51146558 |
| SHMT1 | -1.698571457 | 8.51944014 | -7.738760783 | 6.75E-11 | 2.37E-09 | 14.50945327 |
| AMDHD1 | -1.574726169 | 8.974343584 | -7.734690616 | 6.87E-11 | 2.40E-09 | 14.49275751 |
| SDC4 | -1.669884229 | 10.68868097 | -7.716342289 | 7.42E-11 | 2.57E-09 | 14.41749817 |
| TIMD4 | -2.464595077 | 6.705442914 | -7.71083476 | 7.59E-11 | 2.62E-09 | 14.39490964 |
| GCKR | -2.163915303 | 8.367261074 | -7.708591987 | 7.66E-11 | 2.64E-09 | 14.38571138 |
| TAT | -4.142670615 | 9.478219808 | -7.699458971 | 7.96E-11 | 2.72E-09 | 14.34825566 |
| TM6SF2 | -1.283677881 | 8.343695812 | -7.69007716 | 8.27E-11 | 2.82E-09 | 14.30978199 |
| NFKBIA | -1.134878932 | 11.03841181 | -7.681748501 | 8.57E-11 | 2.91E-09 | 14.27562925 |
| ADTRP | -1.024196487 | 5.108731135 | -7.674655301 | 8.82E-11 | 2.98E-09 | 14.24654424 |
| ALPL | -2.087647198 | 8.425906523 | -7.668675825 | 9.05E-11 | 3.05E-09 | 14.22202711 |
| ABCB11 | -3.866466291 | 7.327504888 | -7.666235913 | 9.14E-11 | 3.07E-09 | 14.21202325 |
| KYNU | -1.868528618 | 8.221023453 | -7.662839726 | 9.27E-11 | 3.10E-09 | 14.19809888 |
| SOD1 | -1.237462996 | 10.93026066 | -7.642984694 | 1.01E-10 | 3.33E-09 | 14.11670029 |
| ACOX1 | -1.415132832 | 9.805179908 | -7.634256419 | 1.04E-10 | 3.44E-09 | 14.08092138 |
| CYP4F2 | -2.618320856 | 9.041582616 | -7.619628519 | 1.11E-10 | 3.64E-09 | 14.02096433 |
| SLC25A25 | -1.277479079 | 8.077007227 | -7.617227241 | 1.12E-10 | 3.67E-09 | 14.01112262 |
| PCCA | -1.072981268 | 8.690310787 | -7.595359619 | 1.23E-10 | 3.98E-09 | 13.92150655 |
| CES3 | -1.952692411 | 7.280709025 | -7.591933506 | 1.25E-10 | 4.02E-09 | 13.90746745 |
| PON1 | -3.657036909 | 9.011450165 | -7.580993648 | 1.30E-10 | 4.19E-09 | 13.86264229 |
| WDR72 | -1.881619719 | 5.341484343 | -7.574947787 | 1.34E-10 | 4.29E-09 | 13.83787174 |
| ACAT1 | -1.797712861 | 9.442849062 | -7.56287428 | 1.41E-10 | 4.48E-09 | 13.78840933 |
| CPM | -1.595343983 | 8.628337013 | -7.52849815 | 1.62E-10 | 5.10E-09 | 13.64760889 |
| AQP3 | -1.672080822 | 9.386451816 | -7.509920416 | 1.75E-10 | 5.48E-09 | 13.57153652 |
| MCL1 | -1.335647002 | 10.79328336 | -7.508674061 | 1.76E-10 | 5.50E-09 | 13.56643345 |
| BCKDHB | -1.001151101 | 9.49502406 | -7.507940198 | 1.77E-10 | 5.51E-09 | 13.56342874 |
| CLYBL | -1.200331291 | 7.841547844 | -7.48339303 | 1.96E-10 | 6.05E-09 | 13.46293709 |
| STEAP4 | -2.911882347 | 7.72961666 | -7.471416835 | 2.06E-10 | 6.28E-09 | 13.41391831 |
| FGF14 | -1.709054448 | 5.651675025 | -7.464526975 | 2.12E-10 | 6.45E-09 | 13.38572091 |
| NR1I3 | -2.155766719 | 8.73141551 | -7.444327536 | 2.30E-10 | 6.96E-09 | 13.30306541 |
| TPST1 | -1.01080371 | 9.014227567 | -7.444320866 | 2.30E-10 | 6.96E-09 | 13.30303812 |
| MT1DP | -1.361251342 | 10.8967498 | -7.442102801 | 2.32E-10 | 7.01E-09 | 13.29396303 |
| EXT1 | -1.171110995 | 9.932720295 | -7.428506449 | 2.46E-10 | 7.33E-09 | 13.23833944 |
| GLRX | -1.414716576 | 8.119262154 | -7.41846872 | 2.56E-10 | 7.62E-09 | 13.19728017 |
| ALAS1 | -1.422984358 | 10.11114844 | -7.415968937 | 2.59E-10 | 7.69E-09 | 13.18705559 |
| CXCL1 | -1.062184135 | 8.049095498 | -7.407612166 | 2.68E-10 | 7.93E-09 | 13.1528771 |
| ASS1 | -1.411768873 | 12.0003997 | -7.395489351 | 2.82E-10 | 8.32E-09 | 13.10330207 |
| AASS | -1.427895774 | 9.052698053 | -7.391007443 | 2.87E-10 | 8.44E-09 | 13.08497566 |
| HPGD | -2.242887476 | 5.292769706 | -7.388133241 | 2.91E-10 | 8.50E-09 | 13.07322366 |
| C1R | -2.21409356 | 11.07150796 | -7.387778018 | 2.91E-10 | 8.50E-09 | 13.07177126 |
| RASD1 | -1.342590775 | 8.488022306 | -7.381674966 | 2.99E-10 | 8.69E-09 | 13.04681872 |
| F8 | -1.645392904 | 7.351375724 | -7.378911337 | 3.02E-10 | 8.78E-09 | 13.03552017 |
| DMGDH | -1.733135352 | 8.62844555 | -7.37557756 | 3.06E-10 | 8.87E-09 | 13.02189122 |
| FCN3 | -3.114421672 | 9.012275542 | -7.354561003 | 3.34E-10 | 9.54E-09 | 12.93598623 |
| GCGR | -1.134010476 | 9.17338123 | -7.348160966 | 3.43E-10 | 9.75E-09 | 12.9098309 |
| HPX | -2.49973736 | 11.54434674 | -7.340362339 | 3.55E-10 | 1.00E-08 | 12.87796294 |
| MAT1A | -2.549802623 | 10.62389722 | -7.337379039 | 3.59E-10 | 1.01E-08 | 12.86577301 |
| VNN1 | -3.82755772 | 8.621666107 | -7.321315182 | 3.84E-10 | 1.08E-08 | 12.80014385 |
| PHYH | -1.404812263 | 9.442082151 | -7.308512256 | 4.05E-10 | 1.13E-08 | 12.74784788 |
| NIPAL1 | -1.215042154 | 7.877663357 | -7.304061834 | 4.13E-10 | 1.15E-08 | 12.72967153 |
| AGPAT2 | -1.537626762 | 9.265214198 | -7.303353963 | 4.14E-10 | 1.15E-08 | 12.72678056 |
| TPMT | -1.162764918 | 7.444360715 | -7.292020853 | 4.34E-10 | 1.20E-08 | 12.68049979 |
| GADD45G | -1.63427269 | 7.84451962 | -7.286818014 | 4.43E-10 | 1.22E-08 | 12.65925564 |
| SULT1B1 | -1.955537094 | 6.451520692 | -7.286238895 | 4.44E-10 | 1.22E-08 | 12.65689109 |
| CRP | -4.497210638 | 8.257084647 | -7.257007442 | 5.02E-10 | 1.37E-08 | 12.53756547 |
| EPAS1 | -1.040193617 | 11.11617601 | -7.240498749 | 5.37E-10 | 1.46E-08 | 12.470199 |
| ST3GAL6 | -1.658030673 | 8.499681183 | -7.22638558 | 5.70E-10 | 1.54E-08 | 12.41262176 |
| APOL1 | -1.770827572 | 8.895137231 | -7.223472583 | 5.76E-10 | 1.56E-08 | 12.40073928 |
| PXDC1 | -1.375943303 | 10.07180869 | -7.211909844 | 6.05E-10 | 1.62E-08 | 12.35357893 |
| FCGR2B | -2.025165101 | 7.39046611 | -7.210299507 | 6.09E-10 | 1.63E-08 | 12.34701163 |
| EDEM1 | -1.109914111 | 8.375413175 | -7.196167546 | 6.46E-10 | 1.72E-08 | 12.28938601 |
| MT1M | -2.757190932 | 9.812826599 | -7.191676819 | 6.58E-10 | 1.75E-08 | 12.2710771 |
| DPYS | -1.794136833 | 9.77327576 | -7.187330445 | 6.70E-10 | 1.78E-08 | 12.25335804 |
| SLC28A1 | -1.154845228 | 6.818254021 | -7.179402065 | 6.92E-10 | 1.83E-08 | 12.22103942 |
| MT1L | -1.548461181 | 10.75807597 | -7.164808259 | 7.35E-10 | 1.94E-08 | 12.16156186 |
| LRRK2 | -1.587516789 | 5.975527224 | -7.163139005 | 7.40E-10 | 1.95E-08 | 12.15475971 |
| STX11 | -1.146958443 | 8.003339685 | -7.159508163 | 7.52E-10 | 1.97E-08 | 12.13996486 |
| GFRA1 | -1.94405853 | 7.705514595 | -7.157566082 | 7.58E-10 | 1.98E-08 | 12.13205171 |
| GDF2 | -1.26283362 | 7.693746852 | -7.151311807 | 7.78E-10 | 2.02E-08 | 12.10657005 |
| FGGY | -1.614629733 | 8.459828907 | -7.149920069 | 7.82E-10 | 2.03E-08 | 12.1009001 |
| NRBP2 | -1.014149399 | 8.39113053 | -7.141779855 | 8.09E-10 | 2.09E-08 | 12.06773965 |
| NR1I2 | -1.216017511 | 7.785432127 | -7.130034889 | 8.49E-10 | 2.19E-08 | 12.01990321 |
| HMGCL | -1.295499653 | 9.33244373 | -7.125835011 | 8.64E-10 | 2.23E-08 | 12.00279986 |
| DAO | -2.480364756 | 7.96778915 | -7.098705175 | 9.67E-10 | 2.46E-08 | 11.89234978 |
| CTPS1 | -1.121128706 | 8.159956954 | -7.096007249 | 9.78E-10 | 2.48E-08 | 11.88136914 |
| CMYA5 | -1.089116192 | 5.973303316 | -7.08602514 | 1.02E-09 | 2.58E-08 | 11.84074654 |
| SDHD | -1.005468311 | 10.58453033 | -7.08575895 | 1.02E-09 | 2.58E-08 | 11.83966337 |
| ADH1C | -2.598482184 | 9.659975185 | -7.084360003 | 1.03E-09 | 2.59E-08 | 11.83397095 |
| LYVE1 | -2.120317351 | 9.239950369 | -7.071576605 | 1.08E-09 | 2.72E-08 | 11.78196143 |
| ECM1 | -1.398929318 | 8.277296346 | -7.068068885 | 1.10E-09 | 2.76E-08 | 11.76769245 |
| ADGRA3 | -1.061271805 | 9.148594842 | -7.067076622 | 1.10E-09 | 2.76E-08 | 11.76365623 |
| TNFSF14 | -1.38618666 | 7.90491786 | -7.054596933 | 1.16E-09 | 2.89E-08 | 11.71289935 |
| NAV2 | -1.167966907 | 8.609369833 | -7.050044179 | 1.18E-09 | 2.94E-08 | 11.69438571 |
| GRAMD1C | -1.730147176 | 6.960419114 | -7.049593457 | 1.19E-09 | 2.94E-08 | 11.69255295 |
| GBE1 | -1.271194008 | 8.216782055 | -7.044260729 | 1.21E-09 | 3.00E-08 | 11.6708699 |
| SAA2 | -4.0396279 | 8.494327281 | -7.035992108 | 1.25E-09 | 3.10E-08 | 11.63725402 |
| PTGS2 | -1.79251305 | 7.121091905 | -7.032434866 | 1.27E-09 | 3.13E-08 | 11.62279387 |
| SH3RF1 | -1.123359207 | 7.775133694 | -7.029866058 | 1.29E-09 | 3.16E-08 | 11.61235235 |
| ABAT | -1.411843431 | 10.5065254 | -7.018785371 | 1.35E-09 | 3.30E-08 | 11.56731861 |
| LONRF2 | -1.031500438 | 6.741064082 | -7.005726501 | 1.42E-09 | 3.45E-08 | 11.51425847 |
| G0S2 | -1.565755013 | 8.952640542 | -7.000595766 | 1.45E-09 | 3.52E-08 | 11.49341551 |
| TGM2 | -1.213478246 | 10.95284787 | -6.991273707 | 1.51E-09 | 3.62E-08 | 11.45555161 |
| RNFT1 | -1.269381586 | 9.031219458 | -6.991171358 | 1.51E-09 | 3.62E-08 | 11.45513593 |
| ETFDH | -1.837848813 | 8.885764568 | -6.973036856 | 1.63E-09 | 3.89E-08 | 11.38149989 |
| SEC14L4 | -1.390011306 | 7.613855158 | -6.971624797 | 1.64E-09 | 3.90E-08 | 11.37576737 |
| AFF3 | -1.165128847 | 7.242259046 | -6.971419327 | 1.64E-09 | 3.90E-08 | 11.37493324 |
| ABCC3 | -1.865841471 | 8.263797294 | -6.968421071 | 1.66E-09 | 3.94E-08 | 11.36276189 |
| CEBPD | -1.106712718 | 10.63498994 | -6.962692532 | 1.70E-09 | 4.01E-08 | 11.33950924 |
| FRMD4B | -1.176133483 | 8.860631957 | -6.960385192 | 1.71E-09 | 4.04E-08 | 11.33014437 |
| CAT | -1.320071294 | 11.01678765 | -6.944925991 | 1.83E-09 | 4.28E-08 | 11.26741202 |
| PIK3AP1 | -1.049781367 | 9.1827581 | -6.93766639 | 1.88E-09 | 4.40E-08 | 11.23796056 |
| SLC38A4 | -2.797226592 | 9.6102422 | -6.90849034 | 2.12E-09 | 4.90E-08 | 11.11964545 |
| SETBP1 | -1.064397275 | 7.934641652 | -6.876223058 | 2.43E-09 | 5.52E-08 | 10.98888859 |
| ENPEP | -1.865208574 | 8.104780749 | -6.860082931 | 2.59E-09 | 5.82E-08 | 10.92352182 |
| MAP3K5 | -1.391991527 | 7.169592015 | -6.857350428 | 2.62E-09 | 5.88E-08 | 10.91245786 |
| SDSL | -1.105813344 | 7.948639573 | -6.856026749 | 2.64E-09 | 5.90E-08 | 10.90709852 |
| C1S | -2.128330635 | 11.30723056 | -6.835262069 | 2.87E-09 | 6.35E-08 | 10.82304907 |
| MRC1 | -1.490675377 | 8.374682697 | -6.830841509 | 2.93E-09 | 6.44E-08 | 10.80516155 |
| SLC16A2 | -1.285000389 | 9.175473042 | -6.818958072 | 3.07E-09 | 6.76E-08 | 10.75708586 |
| AKR1C6P | -1.467848972 | 5.749434072 | -6.815494122 | 3.12E-09 | 6.85E-08 | 10.74307482 |
| CHST9 | -2.623808433 | 6.361593719 | -6.804383053 | 3.26E-09 | 7.13E-08 | 10.69814102 |
| CYP4F11 | -2.496300109 | 8.571621806 | -6.803383908 | 3.28E-09 | 7.15E-08 | 10.69410106 |
| LDHD | -1.233464723 | 8.770802524 | -6.800805739 | 3.31E-09 | 7.20E-08 | 10.6836769 |
| METTL7B | -1.220052351 | 10.20991977 | -6.800561933 | 3.32E-09 | 7.20E-08 | 10.68269117 |
| SAA1 | -3.719471657 | 6.538227536 | -6.798606008 | 3.34E-09 | 7.25E-08 | 10.67478342 |
| REEP6 | -1.535137432 | 10.0757949 | -6.798170665 | 3.35E-09 | 7.25E-08 | 10.67302339 |
| HIGD1A | -1.154192861 | 8.803803118 | -6.797134704 | 3.36E-09 | 7.27E-08 | 10.66883524 |
| CYP4V2 | -1.527119184 | 9.6759094 | -6.79311291 | 3.42E-09 | 7.35E-08 | 10.65257712 |
| TENM1 | -1.512174125 | 6.837184065 | -6.778424551 | 3.63E-09 | 7.78E-08 | 10.59321396 |
| ADM | -1.054214304 | 8.800640824 | -6.777135249 | 3.65E-09 | 7.81E-08 | 10.58800433 |
| FGD4 | -1.485942227 | 7.376803621 | -6.769160859 | 3.77E-09 | 8.02E-08 | 10.55578653 |
| ICAM1 | -1.403920677 | 9.713479453 | -6.768804409 | 3.78E-09 | 8.02E-08 | 10.55434657 |
| DCAF11 | -1.031821892 | 9.497904674 | -6.764954613 | 3.84E-09 | 8.12E-08 | 10.53879539 |
| EXPH5 | -1.387895117 | 6.35391103 | -6.75932542 | 3.93E-09 | 8.28E-08 | 10.51605927 |
| SCN9A | -1.383266416 | 6.55037255 | -6.758327969 | 3.94E-09 | 8.31E-08 | 10.51203095 |
| SLC25A47 | -1.800208399 | 8.723988605 | -6.75604729 | 3.98E-09 | 8.37E-08 | 10.5028206 |
| FXYD1 | -1.601352574 | 9.011132177 | -6.756025362 | 3.98E-09 | 8.37E-08 | 10.50273205 |
| C8A | -2.018868896 | 9.477821757 | -6.745152567 | 4.16E-09 | 8.66E-08 | 10.45883095 |
| RSPO3 | -1.343250249 | 6.212739692 | -6.741327277 | 4.23E-09 | 8.79E-08 | 10.44338867 |
| GPM6A | -1.173498322 | 6.700880576 | -6.737035512 | 4.31E-09 | 8.93E-08 | 10.42606522 |
| ANO1 | -1.434749499 | 7.976907743 | -6.734727515 | 4.35E-09 | 8.97E-08 | 10.41674997 |
| FNDC4 | -1.258877249 | 8.969861722 | -6.730989848 | 4.41E-09 | 9.09E-08 | 10.40166574 |
| EGFR | -1.038826136 | 9.260476339 | -6.725273118 | 4.52E-09 | 9.29E-08 | 10.37859756 |
| NUDT7 | -1.202922304 | 8.799221775 | -6.698345037 | 5.05E-09 | 1.03E-07 | 10.26998704 |
| FAM102A | -1.055994161 | 9.375658284 | -6.685226509 | 5.33E-09 | 1.08E-07 | 10.21710553 |
| CYP4F3 | -1.886247559 | 9.633607777 | -6.685178411 | 5.33E-09 | 1.08E-07 | 10.21691168 |
| TIPARP | -1.312986049 | 8.475292913 | -6.683725473 | 5.36E-09 | 1.09E-07 | 10.21105605 |
| COX7B | -1.248411959 | 7.738862059 | -6.681147207 | 5.42E-09 | 1.09E-07 | 10.20066572 |
| CMBL | -1.101178443 | 9.324678157 | -6.679702773 | 5.45E-09 | 1.10E-07 | 10.19484504 |
| ITIH4 | -1.93312038 | 11.62631382 | -6.676637528 | 5.52E-09 | 1.11E-07 | 10.18249374 |
| TNIK | -1.197385171 | 6.95348784 | -6.670226979 | 5.67E-09 | 1.14E-07 | 10.15666622 |
| PCTP | -1.110869125 | 9.922733991 | -6.663210787 | 5.83E-09 | 1.16E-07 | 10.12840418 |
| BHLHE40 | -1.53639029 | 10.0227816 | -6.657736733 | 5.97E-09 | 1.18E-07 | 10.10635813 |
| SLC20A1 | -1.267519105 | 8.890710663 | -6.657709037 | 5.97E-09 | 1.18E-07 | 10.10624659 |
| BCL3 | -1.042767748 | 9.209404691 | -6.639396857 | 6.43E-09 | 1.26E-07 | 10.0325228 |
| MTHFD1 | -1.292850853 | 9.852553563 | -6.632895988 | 6.61E-09 | 1.29E-07 | 10.00636043 |
| SARDH | -1.0249035 | 9.169069292 | -6.631997372 | 6.63E-09 | 1.29E-07 | 10.00274441 |
| CPT2 | -1.088000967 | 8.244633898 | -6.627319641 | 6.76E-09 | 1.32E-07 | 9.983922854 |
| ACADM | -1.417613432 | 9.61548975 | -6.6257132 | 6.80E-09 | 1.32E-07 | 9.977459717 |
| SQOR | -1.084799689 | 8.945447161 | -6.620825678 | 6.94E-09 | 1.34E-07 | 9.957797872 |
| IRF1 | -1.129933576 | 8.315173152 | -6.61009662 | 7.25E-09 | 1.39E-07 | 9.914646663 |
| ASPDH | -1.35278627 | 8.679386058 | -6.592240512 | 7.80E-09 | 1.48E-07 | 9.842862993 |
| PIGR | -1.800646873 | 7.768049846 | -6.544043994 | 9.51E-09 | 1.77E-07 | 9.649310065 |
| CP | -3.315975501 | 10.71441068 | -6.528450518 | 1.01E-08 | 1.86E-07 | 9.586752852 |
| ATF3 | -1.142735264 | 7.102130482 | -6.524218302 | 1.03E-08 | 1.89E-07 | 9.5697798 |
| CYP3A4 | -3.406126846 | 10.4013461 | -6.514828821 | 1.07E-08 | 1.95E-07 | 9.532132403 |
| TCIM | -1.402207144 | 7.918951338 | -6.511508546 | 1.09E-08 | 1.98E-07 | 9.518822499 |
| RBP5 | -1.267145887 | 9.593071472 | -6.50038163 | 1.14E-08 | 2.05E-07 | 9.474229168 |
| CCNL1 | -1.107352869 | 8.755030643 | -6.474529989 | 1.26E-08 | 2.24E-07 | 9.370688893 |
| GABARAPL1 | -1.161290074 | 10.28414029 | -6.465455295 | 1.31E-08 | 2.31E-07 | 9.334365105 |
| SIAE | -1.079490751 | 7.796181869 | -6.454488629 | 1.37E-08 | 2.40E-07 | 9.290483588 |
| ALDH2 | -1.099808784 | 12.30865062 | -6.450887367 | 1.39E-08 | 2.43E-07 | 9.276077359 |
| FNIP2 | -1.273533036 | 9.112411322 | -6.44871554 | 1.40E-08 | 2.45E-07 | 9.267390224 |
| PRG4 | -2.181730213 | 10.3293135 | -6.444859936 | 1.43E-08 | 2.48E-07 | 9.251969767 |
| ALDH5A1 | -1.110449879 | 8.93309727 | -6.4392293 | 1.46E-08 | 2.53E-07 | 9.229453879 |
| HGF | -2.120626581 | 7.473928641 | -6.433699661 | 1.49E-08 | 2.58E-07 | 9.207346267 |
| C5 | -1.712174938 | 10.77962584 | -6.433511076 | 1.49E-08 | 2.58E-07 | 9.206592376 |
| DEPDC7 | -1.258348302 | 7.230370573 | -6.433000645 | 1.50E-08 | 2.58E-07 | 9.204551897 |
| MT1P3 | -1.567371936 | 9.467233735 | -6.410382519 | 1.64E-08 | 2.80E-07 | 9.114172284 |
| MASP2 | -1.652713808 | 8.985485084 | -6.407545364 | 1.66E-08 | 2.82E-07 | 9.102840559 |
| EPHX2 | -1.355116808 | 9.125024584 | -6.397614441 | 1.73E-08 | 2.92E-07 | 9.063185302 |
| CYB5A | -1.190826221 | 11.72530532 | -6.393086174 | 1.76E-08 | 2.97E-07 | 9.045108254 |
| APOA5 | -1.798968114 | 8.951621646 | -6.391878024 | 1.77E-08 | 2.98E-07 | 9.040285777 |
| OCIAD2 | -1.217978779 | 7.949307531 | -6.386720592 | 1.81E-08 | 3.03E-07 | 9.019701695 |
| MMAA | -1.069248538 | 7.935832381 | -6.385576515 | 1.82E-08 | 3.04E-07 | 9.015136046 |
| ALDH6A1 | -1.096657174 | 11.34883221 | -6.368502856 | 1.95E-08 | 3.22E-07 | 8.947023725 |
| HHIP | -2.049972109 | 7.614706686 | -6.361109848 | 2.01E-08 | 3.31E-07 | 8.917544226 |
| PRSS8 | -1.077305341 | 6.870376793 | -6.355673297 | 2.05E-08 | 3.38E-07 | 8.895871348 |
| GRHL1 | -1.065277402 | 6.871000084 | -6.354671391 | 2.06E-08 | 3.39E-07 | 8.891877726 |
| MT1G | -1.988035343 | 11.74896656 | -6.340390033 | 2.18E-08 | 3.56E-07 | 8.834968563 |
| CYP2C9 | -3.650986468 | 9.930861902 | -6.33563606 | 2.23E-08 | 3.63E-07 | 8.816031603 |
| CYP21A2 | -1.103571191 | 7.566385254 | -6.333486789 | 2.25E-08 | 3.66E-07 | 8.807471349 |
| ARL5B | -1.079714599 | 9.803163276 | -6.316098599 | 2.41E-08 | 3.89E-07 | 8.738242902 |
| APOL6 | -1.44680225 | 8.593131705 | -6.300360338 | 2.57E-08 | 4.10E-07 | 8.67562419 |
| LURAP1L | -1.221718892 | 9.268094549 | -6.295609203 | 2.62E-08 | 4.17E-07 | 8.656728267 |
| COLEC10 | -2.636935684 | 7.981076836 | -6.265409802 | 2.96E-08 | 4.65E-07 | 8.536705407 |
| MT1E | -1.483886311 | 10.67322324 | -6.259677213 | 3.03E-08 | 4.73E-07 | 8.513938741 |
| MYO1B | -1.45699859 | 10.20439325 | -6.257848921 | 3.05E-08 | 4.76E-07 | 8.506678902 |
| EGR2 | -1.285315514 | 8.170435135 | -6.250519108 | 3.14E-08 | 4.89E-07 | 8.477578945 |
| CYP4X1 | -1.782279722 | 7.364414922 | -6.243728108 | 3.23E-08 | 5.00E-07 | 8.450625985 |
| CPNE8 | -1.517220453 | 7.756985969 | -6.236111377 | 3.33E-08 | 5.13E-07 | 8.420404806 |
| TLR4 | -1.368028632 | 8.620706019 | -6.199302033 | 3.87E-08 | 5.90E-07 | 8.274491482 |
| ORM1 | -2.26306021 | 11.39854054 | -6.194664765 | 3.94E-08 | 5.99E-07 | 8.256125431 |
| CYP3A5 | -2.209443382 | 10.13080415 | -6.185665139 | 4.09E-08 | 6.17E-07 | 8.2204926 |
| ZDBF2 | -1.364868818 | 6.050439732 | -6.178889553 | 4.20E-08 | 6.31E-07 | 8.193674732 |
| ABCG2 | -1.275560332 | 7.363475508 | -6.178855627 | 4.20E-08 | 6.31E-07 | 8.19354047 |
| C11orf54 | -1.187999166 | 10.13674016 | -6.176514143 | 4.24E-08 | 6.35E-07 | 8.184274696 |
| PXMP2 | -1.096288456 | 11.38528269 | -6.173075544 | 4.30E-08 | 6.42E-07 | 8.170669106 |
| C4BPB | -1.534303831 | 8.387510299 | -6.168048504 | 4.39E-08 | 6.53E-07 | 8.150782177 |
| SMPDL3A | -1.104350226 | 8.587649811 | -6.162367427 | 4.49E-08 | 6.64E-07 | 8.12831317 |
| SGMS2 | -1.502602409 | 8.186843024 | -6.161026522 | 4.52E-08 | 6.67E-07 | 8.123010626 |
| ARAP2 | -1.688653157 | 6.329427714 | -6.154805098 | 4.63E-08 | 6.83E-07 | 8.098412428 |
| SLC22A7 | -2.215275093 | 9.041358078 | -6.152580242 | 4.67E-08 | 6.89E-07 | 8.089617462 |
| TRIM15 | -1.018029373 | 7.94035897 | -6.13211486 | 5.08E-08 | 7.41E-07 | 8.008757715 |
| FYB2 | -1.606626805 | 6.654519622 | -6.127828783 | 5.17E-08 | 7.53E-07 | 7.991832617 |
| RALGAPA2 | -1.266791101 | 9.237178261 | -6.117608406 | 5.38E-08 | 7.82E-07 | 7.951487077 |
| PCK1 | -2.805776453 | 10.24482045 | -6.115269511 | 5.44E-08 | 7.87E-07 | 7.942256783 |
| ANO5 | -1.311191469 | 4.794460716 | -6.106166132 | 5.64E-08 | 8.14E-07 | 7.906340299 |
| SLC4A4 | -2.253451278 | 8.538107856 | -6.098173167 | 5.82E-08 | 8.38E-07 | 7.874817215 |
| 2-Mar | -1.234395841 | 8.880606127 | -6.086705146 | 6.10E-08 | 8.72E-07 | 7.829609346 |
| GCDH | -1.005647509 | 9.089854549 | -6.086300297 | 6.11E-08 | 8.72E-07 | 7.828013834 |
| MPC1 | -1.33857217 | 9.687879322 | -6.084125828 | 6.16E-08 | 8.78E-07 | 7.819444778 |
| BIRC3 | -1.655151085 | 6.545574709 | -6.082525714 | 6.20E-08 | 8.82E-07 | 7.813139671 |
| CRYBG1 | -1.010182629 | 6.95218128 | -6.074783813 | 6.40E-08 | 9.07E-07 | 7.782640057 |
| TP53INP1 | -1.395954123 | 9.601556377 | -6.062990772 | 6.71E-08 | 9.46E-07 | 7.736202081 |
| DUSP10 | -1.253891525 | 8.661504527 | -6.043978147 | 7.24E-08 | 1.01E-06 | 7.661389871 |
| DBH | -1.017189355 | 7.582802348 | -6.040144519 | 7.36E-08 | 1.03E-06 | 7.646313276 |
| PIK3C2G | -1.823700472 | 7.212122753 | -6.037012172 | 7.45E-08 | 1.04E-06 | 7.633996689 |
| PTPRB | -1.35810929 | 8.225613657 | -6.034168257 | 7.54E-08 | 1.05E-06 | 7.622815842 |
| FTCD | -1.060048431 | 10.13258101 | -6.03098533 | 7.63E-08 | 1.06E-06 | 7.610303989 |
| EGR1 | -1.698506488 | 11.01688618 | -6.026912802 | 7.76E-08 | 1.07E-06 | 7.594297996 |
| ABCA6 | -2.693187666 | 8.606820877 | -6.015034475 | 8.14E-08 | 1.12E-06 | 7.547631464 |
| CRY1 | -1.07740094 | 7.575659789 | -6.011876946 | 8.24E-08 | 1.13E-06 | 7.535230992 |
| GNA14 | -1.158437835 | 6.746308695 | -6.01170098 | 8.25E-08 | 1.13E-06 | 7.534539983 |
| SLC27A2 | -1.760849628 | 10.10975668 | -6.001643882 | 8.59E-08 | 1.17E-06 | 7.495056193 |
| ZSWIM6 | -1.221253564 | 9.175494455 | -5.997771828 | 8.72E-08 | 1.19E-06 | 7.479859877 |
| PLK3 | -1.166301541 | 7.819112909 | -5.991146069 | 8.96E-08 | 1.21E-06 | 7.453863089 |
| HPR | -1.916007487 | 10.66157122 | -5.984300778 | 9.21E-08 | 1.24E-06 | 7.42701394 |
| AGXT2 | -1.633392534 | 8.652081989 | -5.981980812 | 9.29E-08 | 1.24E-06 | 7.417916463 |
| EDNRB | -1.391017261 | 8.62562415 | -5.978455464 | 9.43E-08 | 1.26E-06 | 7.40409425 |
| BZW1 | -1.131356063 | 9.385894265 | -5.970716228 | 9.72E-08 | 1.29E-06 | 7.373758764 |
| IL1RN | -1.493823359 | 9.063876013 | -5.970060106 | 9.75E-08 | 1.29E-06 | 7.371187502 |
| CYP4A22 | -2.422470308 | 9.46329993 | -5.952594964 | 1.05E-07 | 1.38E-06 | 7.302775247 |
| SULT2A1 | -2.308589381 | 10.04925932 | -5.949385929 | 1.06E-07 | 1.39E-06 | 7.29021183 |
| SLC25A20 | -1.141437119 | 8.909730192 | -5.94886798 | 1.06E-07 | 1.39E-06 | 7.288184246 |
| CDO1 | -1.35403464 | 9.842391332 | -5.947336559 | 1.07E-07 | 1.40E-06 | 7.282189598 |
| ATP11C | -1.309410337 | 7.496840588 | -5.942814205 | 1.09E-07 | 1.43E-06 | 7.264489882 |
| ZBTB21 | -1.074182785 | 7.757098951 | -5.917249405 | 1.20E-07 | 1.56E-06 | 7.164511332 |
| CYP8B1 | -2.320667254 | 8.968624868 | -5.914596747 | 1.22E-07 | 1.57E-06 | 7.154144957 |
| ENO3 | -1.201950769 | 8.041671171 | -5.911675541 | 1.23E-07 | 1.59E-06 | 7.142730782 |
| FMO4 | -1.417054258 | 8.353776986 | -5.891841166 | 1.33E-07 | 1.70E-06 | 7.065277374 |
| EHHADH | -1.603269285 | 9.46687445 | -5.89094123 | 1.34E-07 | 1.70E-06 | 7.061765048 |
| PHLDA1 | -1.028442315 | 9.443524386 | -5.890803785 | 1.34E-07 | 1.70E-06 | 7.061228632 |
| DPYD | -1.175953483 | 9.436632619 | -5.890144486 | 1.34E-07 | 1.71E-06 | 7.058655602 |
| LBP | -2.546604715 | 10.90845602 | -5.889798834 | 1.34E-07 | 1.71E-06 | 7.057306672 |
| TSPYL5 | -1.428926813 | 7.421227147 | -5.887607525 | 1.36E-07 | 1.72E-06 | 7.048755513 |
| IL13RA1 | -1.040955104 | 10.00329481 | -5.881978412 | 1.39E-07 | 1.75E-06 | 7.026793567 |
| ALPK2 | -1.362124034 | 6.336399715 | -5.88063727 | 1.39E-07 | 1.76E-06 | 7.021562087 |
| SOCS3 | -1.395261038 | 8.055945994 | -5.862286111 | 1.50E-07 | 1.88E-06 | 6.950016406 |
| JUNB | -1.377878965 | 10.15621508 | -5.855212619 | 1.54E-07 | 1.93E-06 | 6.922457936 |
| HAO1 | -2.231521865 | 8.966188806 | -5.854550845 | 1.55E-07 | 1.93E-06 | 6.91988019 |
| CLU | -1.532959124 | 11.75394004 | -5.844788033 | 1.61E-07 | 2.00E-06 | 6.881862898 |
| IGF1 | -1.612198219 | 7.414840588 | -5.841335951 | 1.63E-07 | 2.03E-06 | 6.868425034 |
| ACAA1 | -1.113839654 | 9.431704662 | -5.83834358 | 1.65E-07 | 2.04E-06 | 6.856778748 |
| ABCA9 | -2.13249771 | 7.682218424 | -5.834557719 | 1.68E-07 | 2.07E-06 | 6.842046947 |
| VNN2 | -1.406022342 | 6.562896655 | -5.827571678 | 1.72E-07 | 2.12E-06 | 6.814870487 |
| ACADS | -1.146895666 | 9.095862381 | -5.827115728 | 1.73E-07 | 2.12E-06 | 6.813097154 |
| ACTR3C | -1.029192454 | 7.452866992 | -5.823176331 | 1.75E-07 | 2.15E-06 | 6.7977775 |
| HSD17B14 | -1.078573646 | 7.301304991 | -5.820876216 | 1.77E-07 | 2.17E-06 | 6.788834288 |
| SERPINB9 | -1.2626185 | 8.252687977 | -5.809998391 | 1.85E-07 | 2.25E-06 | 6.746555138 |
| PAMR1 | -1.518919203 | 7.327767144 | -5.808604833 | 1.86E-07 | 2.26E-06 | 6.741140616 |
| SERPINA11 | -1.392106429 | 11.37244836 | -5.806723746 | 1.87E-07 | 2.28E-06 | 6.733832524 |
| SLC37A4 | -1.096894262 | 10.1344444 | -5.801579758 | 1.91E-07 | 2.32E-06 | 6.713851896 |
| AGXT | -1.616539976 | 11.03848259 | -5.799581796 | 1.93E-07 | 2.33E-06 | 6.706092839 |
| PDE7B | -1.008009804 | 6.796761811 | -5.79710989 | 1.95E-07 | 2.35E-06 | 6.69649444 |
| CYP4F22 | -1.352905249 | 6.836769924 | -5.794938101 | 1.96E-07 | 2.37E-06 | 6.688062498 |
| ALDOB | -2.277243571 | 11.93667078 | -5.792236929 | 1.98E-07 | 2.39E-06 | 6.677576684 |
| MT2A | -1.153573749 | 13.15313437 | -5.759900009 | 2.26E-07 | 2.67E-06 | 6.55217194 |
| NPY1R | -1.717677799 | 5.854515512 | -5.743407037 | 2.41E-07 | 2.83E-06 | 6.488301233 |
| IRF6 | -1.356453888 | 8.190127652 | -5.741455338 | 2.43E-07 | 2.84E-06 | 6.48074715 |
| ACSM3 | -1.563849963 | 8.315471543 | -5.738662022 | 2.45E-07 | 2.86E-06 | 6.469937078 |
| AKAP12 | -1.331145366 | 7.564397049 | -5.737745154 | 2.46E-07 | 2.87E-06 | 6.466389205 |
| TUT7 | -1.140051511 | 8.182261456 | -5.736613408 | 2.47E-07 | 2.88E-06 | 6.462010113 |
| ZFAND5 | -1.051681676 | 10.07854015 | -5.735146639 | 2.49E-07 | 2.90E-06 | 6.456335143 |
| ZBTB16 | -1.087572922 | 8.563221454 | -5.728817112 | 2.55E-07 | 2.96E-06 | 6.431851652 |
| PLA2G2A | -2.737346426 | 8.273103125 | -5.72765997 | 2.56E-07 | 2.96E-06 | 6.427376658 |
| PIPOX | -1.828177109 | 10.53364511 | -5.725987459 | 2.58E-07 | 2.98E-06 | 6.420909122 |
| KLKB1 | -2.247143217 | 9.881950285 | -5.723912464 | 2.60E-07 | 2.99E-06 | 6.412886091 |
| ABCC9 | -1.889754084 | 7.774271915 | -5.716897761 | 2.67E-07 | 3.06E-06 | 6.385770841 |
| SLCO2B1 | -1.029481797 | 10.72521978 | -5.707100431 | 2.78E-07 | 3.16E-06 | 6.347918351 |
| MASP1 | -1.377494113 | 10.11085694 | -5.705625723 | 2.80E-07 | 3.18E-06 | 6.342222659 |
| KLF4 | -1.042089011 | 7.296209827 | -5.70100182 | 2.85E-07 | 3.23E-06 | 6.324367262 |
| DUSP1 | -1.524847514 | 11.43281768 | -5.695559667 | 2.91E-07 | 3.29E-06 | 6.303358524 |
| ARG1 | -1.90331457 | 9.869395902 | -5.689829834 | 2.98E-07 | 3.36E-06 | 6.281246693 |
| DBT | -1.028510454 | 8.332638867 | -5.689732523 | 2.98E-07 | 3.36E-06 | 6.280871229 |
| SGK1 | -1.069414072 | 7.957122126 | -5.683399512 | 3.05E-07 | 3.43E-06 | 6.256440768 |
| PARP9 | -1.16244947 | 7.951414772 | -5.679259953 | 3.10E-07 | 3.48E-06 | 6.240476933 |
| MBNL2 | -1.256902295 | 10.32683399 | -5.648778522 | 3.50E-07 | 3.89E-06 | 6.123052777 |
| STAT4 | -1.039329313 | 6.657813436 | -5.642511141 | 3.59E-07 | 3.98E-06 | 6.098936267 |
| SAMD12 | -1.14646719 | 5.446457873 | -5.639342405 | 3.64E-07 | 4.02E-06 | 6.086746749 |
| MS4A6A | -1.298170013 | 8.603315855 | -5.638180743 | 3.65E-07 | 4.03E-06 | 6.08227866 |
| TMEM45A | -1.027585152 | 8.112233128 | -5.636295193 | 3.68E-07 | 4.06E-06 | 6.075026987 |
| ABCA10 | -1.487947174 | 5.893075969 | -5.629723528 | 3.78E-07 | 4.15E-06 | 6.049759586 |
| SLITRK6 | -1.224560467 | 4.911418995 | -5.625577981 | 3.84E-07 | 4.21E-06 | 6.033825736 |
| HPD | -2.3164892 | 10.91384572 | -5.615414525 | 3.99E-07 | 4.36E-06 | 5.994779067 |
| RAB17 | -1.015352622 | 8.237992078 | -5.610871404 | 4.07E-07 | 4.44E-06 | 5.977333128 |
| CD163 | -1.389937258 | 8.299919623 | -5.606128869 | 4.14E-07 | 4.51E-06 | 5.959126801 |
| KDM7A | -1.178887094 | 7.813161187 | -5.597089384 | 4.29E-07 | 4.66E-06 | 5.924440006 |
| BHMT | -1.850261398 | 10.66769889 | -5.594533013 | 4.34E-07 | 4.70E-06 | 5.914634206 |
| SHTN1 | -1.260750142 | 8.219331078 | -5.587124833 | 4.47E-07 | 4.83E-06 | 5.886226818 |
| INHBE | -1.43990795 | 8.022678632 | -5.580387721 | 4.59E-07 | 4.95E-06 | 5.860404501 |
| IL1B | -1.235903749 | 6.910321357 | -5.570495959 | 4.77E-07 | 5.12E-06 | 5.822511353 |
| ABCB4 | -1.614050705 | 9.379801107 | -5.566213378 | 4.85E-07 | 5.19E-06 | 5.806113311 |
| PLA1A | -1.044330527 | 8.674862542 | -5.563768199 | 4.90E-07 | 5.23E-06 | 5.796752755 |
| FAM180A | -1.326205683 | 7.51635319 | -5.562106074 | 4.93E-07 | 5.26E-06 | 5.790390722 |
| CFI | -1.369182288 | 9.995034561 | -5.549877084 | 5.17E-07 | 5.48E-06 | 5.743603771 |
| INHBC | -1.65141704 | 9.106961935 | -5.541569262 | 5.34E-07 | 5.64E-06 | 5.711840379 |
| ATP2B2 | -1.04190301 | 8.854822247 | -5.527958298 | 5.63E-07 | 5.90E-06 | 5.65983933 |
| IFITM10 | -1.007562235 | 7.740333956 | -5.507949833 | 6.09E-07 | 6.34E-06 | 5.583482575 |
| ARRDC3 | -1.625772611 | 9.624455352 | -5.503572566 | 6.20E-07 | 6.44E-06 | 5.56679171 |
| IL18R1 | -1.090606582 | 5.973228798 | -5.4997241 | 6.29E-07 | 6.52E-06 | 5.552121308 |
| PLAC8 | -1.550180385 | 7.412550811 | -5.499400708 | 6.30E-07 | 6.53E-06 | 5.550888708 |
| DCXR | -1.013458547 | 12.24466143 | -5.497691168 | 6.34E-07 | 6.56E-06 | 5.544373296 |
| ACOX2 | -1.113125292 | 9.857347311 | -5.493781361 | 6.44E-07 | 6.64E-06 | 5.529475065 |
| ANGPTL3 | -2.033491065 | 9.899615279 | -5.489293689 | 6.56E-07 | 6.74E-06 | 5.512379801 |
| SIPA1L2 | -1.070849909 | 7.600229134 | -5.485468491 | 6.65E-07 | 6.83E-06 | 5.497812306 |
| CREB3L3 | -1.45403617 | 10.28083445 | -5.477928519 | 6.85E-07 | 7.01E-06 | 5.46910906 |
| UGT2B10 | -2.427128402 | 9.364643402 | -5.46935751 | 7.09E-07 | 7.22E-06 | 5.436498993 |
| SATB1 | -1.344668052 | 7.510255114 | -5.469229395 | 7.09E-07 | 7.22E-06 | 5.436011703 |
| ESRP2 | -1.041098825 | 8.551281391 | -5.465621708 | 7.19E-07 | 7.30E-06 | 5.422291471 |
| USP12 | -1.01956955 | 9.393204708 | -5.464762996 | 7.22E-07 | 7.32E-06 | 5.41902625 |
| CES2 | -1.278267246 | 9.532573977 | -5.459219813 | 7.37E-07 | 7.47E-06 | 5.397953191 |
| AOX1 | -1.749800892 | 10.70206031 | -5.450880573 | 7.62E-07 | 7.66E-06 | 5.36626596 |
| PROS1 | -1.133434031 | 10.63229841 | -5.449882317 | 7.65E-07 | 7.68E-06 | 5.362474051 |
| NR4A3 | -1.188026758 | 7.242152388 | -5.449188396 | 7.67E-07 | 7.70E-06 | 5.359838327 |
| TFR2 | -1.688425167 | 10.65310105 | -5.449125251 | 7.67E-07 | 7.70E-06 | 5.359598489 |
| CD274 | -1.126249485 | 5.570424235 | -5.437351269 | 8.03E-07 | 8.04E-06 | 5.314897115 |
| SERPINA3 | -1.588639569 | 12.37200071 | -5.435872716 | 8.08E-07 | 8.08E-06 | 5.309286233 |
| CAST | -1.009531916 | 9.369462561 | -5.434316193 | 8.13E-07 | 8.12E-06 | 5.303380098 |
| CIDEB | -1.071397717 | 8.983403615 | -5.427322436 | 8.35E-07 | 8.33E-06 | 5.276850748 |
| PTGR1 | -1.171236964 | 9.498888221 | -5.427086154 | 8.36E-07 | 8.33E-06 | 5.275954691 |
| PLAAT4 | -1.040407593 | 8.239007107 | -5.388640147 | 9.71E-07 | 9.56E-06 | 5.13035727 |
| F11 | -1.93441723 | 9.348138788 | -5.386026678 | 9.81E-07 | 9.64E-06 | 5.12047458 |
| CES1P1 | -1.808746726 | 10.99378556 | -5.384859904 | 9.85E-07 | 9.68E-06 | 5.116063096 |
| CPED1 | -2.096571745 | 7.996236693 | -5.375981372 | 1.02E-06 | 9.98E-06 | 5.082506352 |
| SLCO1B7 | -2.19889763 | 6.837261794 | -5.375439956 | 1.02E-06 | 9.99E-06 | 5.080460759 |
| CYP2J2 | -1.276275315 | 9.343576053 | -5.370640411 | 1.04E-06 | 1.01E-05 | 5.062330507 |
| FOSB | -1.928989303 | 8.890493717 | -5.369390953 | 1.05E-06 | 1.02E-05 | 5.057611737 |
| SELENOP | -1.025399653 | 12.15717354 | -5.343310224 | 1.16E-06 | 1.11E-05 | 4.959213373 |
| SORBS1 | -1.195340827 | 8.663241531 | -5.335269676 | 1.19E-06 | 1.15E-05 | 4.92891622 |
| CASP4 | -1.144683702 | 8.111183062 | -5.315012367 | 1.29E-06 | 1.22E-05 | 4.852667017 |
| TTR | -1.197371498 | 12.50651778 | -5.301868232 | 1.36E-06 | 1.28E-05 | 4.803254716 |
| ARHGEF12 | -1.074310965 | 9.751956825 | -5.298818767 | 1.38E-06 | 1.29E-05 | 4.79179806 |
| CYFIP2 | -1.01432092 | 8.713593095 | -5.296739571 | 1.39E-06 | 1.30E-05 | 4.783988181 |
| SORL1 | -1.003158382 | 10.04654353 | -5.295400283 | 1.39E-06 | 1.31E-05 | 4.778958207 |
| CYP1A1 | -1.868266708 | 6.820988858 | -5.292832176 | 1.41E-06 | 1.32E-05 | 4.769314594 |
| HOOK1 | -1.347243316 | 7.492766774 | -5.289708203 | 1.42E-06 | 1.33E-05 | 4.757586194 |
| ACADSB | -1.368109979 | 10.34117637 | -5.289144216 | 1.43E-06 | 1.34E-05 | 4.755469109 |
| SLAIN1 | -1.279166551 | 7.421556275 | -5.272009289 | 1.53E-06 | 1.42E-05 | 4.691192265 |
| TREM1 | -1.137825627 | 6.287333926 | -5.263391536 | 1.58E-06 | 1.46E-05 | 4.658897561 |
| MGST1 | -1.088134823 | 10.93638384 | -5.257444494 | 1.61E-06 | 1.49E-05 | 4.636623944 |
| CFH | -1.983634702 | 10.7111686 | -5.23959702 | 1.73E-06 | 1.58E-05 | 4.569841919 |
| CD55 | -1.175163438 | 9.282393868 | -5.235258668 | 1.76E-06 | 1.60E-05 | 4.553622841 |
| ADAMTS1 | -1.389184089 | 8.107592379 | -5.232595404 | 1.78E-06 | 1.61E-05 | 4.543668909 |
| LIFR | -2.045839689 | 8.393549227 | -5.217155527 | 1.88E-06 | 1.71E-05 | 4.48600419 |
| MBOAT1 | -1.336646891 | 7.77533446 | -5.21693766 | 1.89E-06 | 1.71E-05 | 4.485191016 |
| ECHDC3 | -1.353304407 | 8.225617243 | -5.216017313 | 1.89E-06 | 1.71E-05 | 4.481756023 |
| NUGGC | -1.079578347 | 7.677361383 | -5.212637771 | 1.92E-06 | 1.73E-05 | 4.469144823 |
| BCHE | -1.498220834 | 6.966206378 | -5.180502923 | 2.17E-06 | 1.94E-05 | 4.349401883 |
| TEK | -1.128629119 | 7.649078022 | -5.175441552 | 2.21E-06 | 1.97E-05 | 4.330570515 |
| IL6 | -1.157593263 | 5.681708177 | -5.164098424 | 2.31E-06 | 2.05E-05 | 4.288395726 |
| HGD | -1.42630552 | 11.09094829 | -5.157867392 | 2.37E-06 | 2.09E-05 | 4.265245028 |
| CYP3A43 | -2.266173171 | 7.688965645 | -5.149538839 | 2.44E-06 | 2.15E-05 | 4.234319963 |
| SLC17A4 | -1.804983936 | 7.072212308 | -5.144235724 | 2.49E-06 | 2.19E-05 | 4.214639971 |
| NR5A2 | -1.065011164 | 9.825565998 | -5.138674827 | 2.55E-06 | 2.23E-05 | 4.194012738 |
| FAH | -1.004596143 | 10.41335438 | -5.129168169 | 2.64E-06 | 2.30E-05 | 4.158771698 |
| CDH19 | -1.179678586 | 5.431876489 | -5.127410146 | 2.66E-06 | 2.31E-05 | 4.152257829 |
| MST1 | -1.071204952 | 10.34153185 | -5.117765711 | 2.76E-06 | 2.38E-05 | 4.116540305 |
| OTC | -1.926044625 | 9.026919986 | -5.11617909 | 2.77E-06 | 2.39E-05 | 4.110667161 |
| AZGP1 | -1.704478232 | 11.23984828 | -5.112022291 | 2.82E-06 | 2.43E-05 | 4.095283834 |
| CTSO | -1.197586245 | 7.714077744 | -5.088860997 | 3.08E-06 | 2.61E-05 | 4.009669599 |
| IGFBP3 | -1.098821767 | 9.41881788 | -5.081517832 | 3.17E-06 | 2.68E-05 | 3.982561732 |
| CNGA1 | -1.211060387 | 5.909247031 | -5.074894312 | 3.25E-06 | 2.74E-05 | 3.958125304 |
| MYOM1 | -1.574855932 | 8.509086885 | -5.070338924 | 3.30E-06 | 2.78E-05 | 3.941327105 |
| PAIP2B | -1.072590907 | 8.430990583 | -5.069577113 | 3.31E-06 | 2.78E-05 | 3.938518547 |
| FUOM | -1.102448726 | 9.81834688 | -5.06633323 | 3.35E-06 | 2.81E-05 | 3.92656145 |
| TMEM71 | -1.016562327 | 5.777044201 | -5.059740197 | 3.44E-06 | 2.88E-05 | 3.902269709 |
| RORA | -1.250873444 | 8.633782174 | -5.05384621 | 3.52E-06 | 2.93E-05 | 3.880565485 |
| AKR1C4 | -2.501051513 | 8.062141879 | -5.046482053 | 3.62E-06 | 3.00E-05 | 3.853463303 |
| RNF19A | -1.08832396 | 8.717621572 | -5.043880475 | 3.65E-06 | 3.02E-05 | 3.84389298 |
| GSAP | -1.091324073 | 8.29718331 | -5.03970698 | 3.71E-06 | 3.06E-05 | 3.828544715 |
| CD5L | -1.719419838 | 9.626460529 | -5.038553867 | 3.73E-06 | 3.08E-05 | 3.824305082 |
| KAT2B | -1.240185391 | 8.464884366 | -5.030108309 | 3.85E-06 | 3.16E-05 | 3.793266677 |
| CES1 | -1.641967737 | 11.86376331 | -5.029175281 | 3.86E-06 | 3.17E-05 | 3.789839126 |
| NOSTRIN | -1.027501011 | 6.051472491 | -5.025957474 | 3.91E-06 | 3.21E-05 | 3.778020453 |
| EPHA2 | -1.049996252 | 8.528298637 | -5.015155855 | 4.07E-06 | 3.32E-05 | 3.73837215 |
| CXCR1 | -1.16384153 | 6.060992528 | -5.012727937 | 4.11E-06 | 3.35E-05 | 3.729465561 |
| IL32 | -1.202926231 | 9.148973532 | -5.003563295 | 4.26E-06 | 3.46E-05 | 3.695863529 |
| ARHGAP42 | -1.212753855 | 7.780515769 | -5.00275122 | 4.27E-06 | 3.46E-05 | 3.692887413 |
| ANGPTL4 | -1.271736854 | 8.087776447 | -5.001389161 | 4.29E-06 | 3.47E-05 | 3.687896188 |
| NR0B2 | -1.294846359 | 9.679505536 | -4.994793124 | 4.40E-06 | 3.56E-05 | 3.663733949 |
| CFHR2 | -2.317287042 | 10.47769825 | -4.994521116 | 4.40E-06 | 3.56E-05 | 3.662737855 |
| CYP4A11 | -2.175394796 | 11.14500258 | -4.992108642 | 4.44E-06 | 3.58E-05 | 3.653904458 |
| KIAA0040 | -1.207572413 | 7.114640217 | -4.989128972 | 4.49E-06 | 3.61E-05 | 3.642996922 |
| IL33 | -1.084048523 | 5.877122683 | -4.987372029 | 4.52E-06 | 3.63E-05 | 3.636566759 |
| ALDH1A1 | -1.570061831 | 10.24365286 | -4.972933851 | 4.78E-06 | 3.81E-05 | 3.583764269 |
| BHMT2 | -1.17995638 | 10.45581592 | -4.972094714 | 4.79E-06 | 3.82E-05 | 3.580697579 |
| CXCL14 | -1.667635436 | 8.337106011 | -4.968333976 | 4.86E-06 | 3.87E-05 | 3.566956601 |
| RTP3 | -1.266690802 | 6.857375131 | -4.964601904 | 4.93E-06 | 3.91E-05 | 3.553325078 |
| ITGB3 | -1.106211182 | 7.751870703 | -4.954592902 | 5.12E-06 | 4.05E-05 | 3.516790145 |
| BAAT | -2.621381332 | 9.988489083 | -4.950047421 | 5.21E-06 | 4.10E-05 | 3.500209425 |
| APCS | -2.952924941 | 10.19914146 | -4.947457574 | 5.26E-06 | 4.14E-05 | 3.490765491 |
| ADH1B | -2.384028972 | 10.7422025 | -4.946327696 | 5.28E-06 | 4.15E-05 | 3.486646083 |
| GBP2 | -1.288117891 | 7.573701679 | -4.909542359 | 6.07E-06 | 4.69E-05 | 3.352770006 |
| FOS | -1.816576205 | 10.31658309 | -4.888285955 | 6.57E-06 | 5.03E-05 | 3.275623177 |
| MMRN1 | -1.418021417 | 6.534398126 | -4.888211149 | 6.57E-06 | 5.03E-05 | 3.275351957 |
| RAPGEF5 | -1.188094992 | 7.559304689 | -4.879485343 | 6.79E-06 | 5.18E-05 | 3.243728941 |
| FGFR2 | -1.75062725 | 7.975636394 | -4.87500715 | 6.90E-06 | 5.24E-05 | 3.227509999 |
| FNDC5 | -1.069537564 | 7.843687889 | -4.873031083 | 6.96E-06 | 5.28E-05 | 3.220355402 |
| SERPINC1 | -1.531194672 | 12.20814514 | -4.872130024 | 6.98E-06 | 5.29E-05 | 3.217093462 |
| CFB | -1.284805711 | 12.27298011 | -4.8697828 | 7.04E-06 | 5.32E-05 | 3.208597582 |
| SLC6A1 | -1.668664104 | 9.690955236 | -4.860469125 | 7.29E-06 | 5.50E-05 | 3.174905513 |
| F13B | -1.834129735 | 8.35634441 | -4.856011466 | 7.41E-06 | 5.57E-05 | 3.158790879 |
| IL1RAP | -1.155173795 | 8.904176828 | -4.855892086 | 7.42E-06 | 5.57E-05 | 3.158359413 |
| MT1F | -1.667852052 | 11.65500106 | -4.842627006 | 7.79E-06 | 5.81E-05 | 3.110447933 |
| CFHR1 | -1.793046832 | 11.81908993 | -4.839600965 | 7.88E-06 | 5.87E-05 | 3.099527121 |
| MGAM | -1.09192296 | 5.384513314 | -4.833790686 | 8.06E-06 | 5.98E-05 | 3.078567339 |
| SLC17A3 | -1.379548433 | 7.787604913 | -4.823225555 | 8.38E-06 | 6.20E-05 | 3.040486134 |
| HP | -1.966029722 | 12.4213168 | -4.787276415 | 9.58E-06 | 6.98E-05 | 2.911212651 |
| NR4A2 | -1.404510902 | 7.697236522 | -4.784377823 | 9.68E-06 | 7.04E-05 | 2.900809797 |
| C4BPA | -1.764002659 | 11.69099348 | -4.765885236 | 1.04E-05 | 7.48E-05 | 2.8345138 |
| GCLC | -1.067898803 | 9.435874215 | -4.748098553 | 1.11E-05 | 7.91E-05 | 2.770867679 |
| MID2 | -1.216881626 | 7.660000771 | -4.745991217 | 1.12E-05 | 7.95E-05 | 2.763334782 |
| ERRFI1 | -1.346218089 | 11.17203533 | -4.730037729 | 1.18E-05 | 8.37E-05 | 2.706361144 |
| CR1 | -1.11445478 | 6.930549687 | -4.729657217 | 1.19E-05 | 8.37E-05 | 2.705003409 |
| PROZ | -1.234475343 | 9.073907749 | -4.729574431 | 1.19E-05 | 8.37E-05 | 2.704708023 |
| RGN | -1.052455879 | 9.001851251 | -4.724987213 | 1.21E-05 | 8.49E-05 | 2.688344443 |
| FMO3 | -2.866345064 | 9.614276427 | -4.719492265 | 1.23E-05 | 8.63E-05 | 2.668753218 |
| ADAMTSL3 | -1.246711106 | 8.141320585 | -4.714730918 | 1.25E-05 | 8.77E-05 | 2.651786711 |
| XAF1 | -1.13959136 | 8.486683893 | -4.702857195 | 1.31E-05 | 9.12E-05 | 2.609513428 |
| CPEB4 | -1.130353999 | 9.124154437 | -4.700082488 | 1.32E-05 | 9.18E-05 | 2.599642515 |
| CLDN1 | -1.024300585 | 11.82015717 | -4.697617681 | 1.33E-05 | 9.26E-05 | 2.590876511 |
| CTSS | -1.028706521 | 8.566445387 | -4.681767402 | 1.42E-05 | 9.74E-05 | 2.534560871 |
| DSG1 | -1.205557901 | 6.454691886 | -4.676777305 | 1.44E-05 | 9.89E-05 | 2.516851067 |
| CYP4F12 | -1.213201967 | 7.931858126 | -4.653007834 | 1.57E-05 | 0.000107037 | 2.432625013 |
| CCDC68 | -1.125337098 | 5.74211801 | -4.651783591 | 1.58E-05 | 0.000107433 | 2.428292873 |
| ARNTL | -1.124394573 | 7.388008057 | -4.649786258 | 1.59E-05 | 0.000108062 | 2.421226309 |
| C4A | -1.20649518 | 12.37460778 | -4.640346976 | 1.65E-05 | 0.000111513 | 2.387851102 |
| TTPA | -1.162828566 | 9.550949939 | -4.636227719 | 1.67E-05 | 0.000112844 | 2.373297201 |
| ADH4 | -2.168715834 | 9.580112121 | -4.626502563 | 1.73E-05 | 0.000116561 | 2.338963193 |
| SGK2 | -1.020780987 | 8.560580078 | -4.610518605 | 1.84E-05 | 0.000122705 | 2.282613524 |
| NR4A1 | -1.12686093 | 8.923052719 | -4.604707897 | 1.88E-05 | 0.000125117 | 2.26215347 |
| AADAC | -2.357571867 | 9.62447244 | -4.593877994 | 1.95E-05 | 0.000129614 | 2.22405603 |
| PLIN2 | -1.044324793 | 10.72818996 | -4.584252125 | 2.02E-05 | 0.000133775 | 2.190233259 |
| KNG1 | -1.659081422 | 11.99978471 | -4.549811866 | 2.29E-05 | 0.000149182 | 2.06952301 |
| P4HA1 | -1.225325954 | 8.931017462 | -4.528133644 | 2.48E-05 | 0.000159742 | 1.993788335 |
| TRANK1 | -1.03269502 | 7.861728386 | -4.524007733 | 2.52E-05 | 0.000161846 | 1.979395798 |
| UGT2B4 | -1.753235967 | 11.41900948 | -4.500641276 | 2.74E-05 | 0.000174318 | 1.898017587 |
| SLCO1B1 | -2.200427568 | 9.909398132 | -4.498432544 | 2.76E-05 | 0.00017542 | 1.890336873 |
| DIO1 | -1.535114805 | 9.623925245 | -4.495613074 | 2.79E-05 | 0.000176859 | 1.880535292 |
| PAPPA2 | -1.344518709 | 7.012338887 | -4.475695847 | 3.00E-05 | 0.000188084 | 1.811389076 |
| IFITM1 | -1.052899771 | 10.78849991 | -4.464544255 | 3.12E-05 | 0.000194761 | 1.772746468 |
| GATM | -1.638368884 | 11.36395367 | -4.434157269 | 3.48E-05 | 0.000213995 | 1.667714162 |
| GBP4 | -1.091022495 | 7.429763427 | -4.429528526 | 3.54E-05 | 0.000217156 | 1.651749192 |
| NFIB | -1.289458559 | 9.088947175 | -4.425020213 | 3.60E-05 | 0.00022034 | 1.636208341 |
| FGG | -1.552003774 | 11.78722135 | -4.41651481 | 3.71E-05 | 0.000226355 | 1.606912462 |
| RGS2 | -1.409519991 | 7.747739172 | -4.412112377 | 3.77E-05 | 0.000229731 | 1.59176091 |
| AFM | -2.064896297 | 9.857146124 | -4.36754659 | 4.42E-05 | 0.000263935 | 1.438850896 |
| LEAP2 | -1.451919722 | 10.22830264 | -4.353602875 | 4.65E-05 | 0.000275457 | 1.391185284 |
| MAOA | -1.032437115 | 9.147216835 | -4.34822912 | 4.74E-05 | 0.000279807 | 1.372838159 |
| ABCA8 | -1.566168308 | 7.87368119 | -4.344766218 | 4.79E-05 | 0.000283098 | 1.361021792 |
| MLIP | -1.267348062 | 6.15665293 | -4.295232274 | 5.71E-05 | 0.000330226 | 1.192577996 |
| TLR3 | -1.105264906 | 7.312784639 | -4.287584779 | 5.87E-05 | 0.000338239 | 1.166669424 |
| SYBU | -1.100629271 | 8.138949708 | -4.271217045 | 6.22E-05 | 0.000356123 | 1.111306261 |
| F2 | -1.02289347 | 11.49185781 | -4.267138927 | 6.31E-05 | 0.000360467 | 1.09753099 |
| HMGCS2 | -1.363579993 | 12.14736531 | -4.253504701 | 6.62E-05 | 0.000376378 | 1.051531288 |
| HAND2-AS1 | -1.049389577 | 6.51004825 | -4.249219909 | 6.72E-05 | 0.000381291 | 1.037092513 |
| CCL16 | -1.781986549 | 8.448706771 | -4.233337944 | 7.11E-05 | 0.000399812 | 0.983646901 |
| SLC2A3 | -1.034552459 | 8.770003142 | -4.214929559 | 7.58E-05 | 0.00042345 | 0.921844019 |
| SLC22A25 | -1.336550758 | 8.298297653 | -4.209941044 | 7.72E-05 | 0.000430151 | 0.905122831 |
| FGB | -1.415966481 | 12.34304602 | -4.197782689 | 8.05E-05 | 0.000445946 | 0.864416988 |
| NFIA | -1.037698175 | 9.934495355 | -4.176417486 | 8.68E-05 | 0.000477412 | 0.793053247 |
| PBLD | -1.11389106 | 9.252659939 | -4.113961949 | 0.000107783 | 0.00057614 | 0.585670693 |
| ADH1A | -1.361692485 | 11.28552174 | -4.112663511 | 0.000108269 | 0.000578407 | 0.581378913 |
| TNFSF10 | -1.139338856 | 9.987031748 | -4.053192638 | 0.000132919 | 0.00069279 | 0.385678676 |
| ITIH3 | -1.148670986 | 11.89926517 | -4.046013524 | 0.000136237 | 0.000707342 | 0.36217073 |
| ANGPTL1 | -1.209122408 | 5.827248268 | -4.031782885 | 0.000143052 | 0.000738648 | 0.315647397 |
| NAT8 | -1.355366497 | 8.866738003 | -3.987370984 | 0.000166494 | 0.000839877 | 0.171098502 |
| SERPINA6 | -1.170268775 | 11.17982997 | -3.910452963 | 0.00021609 | 0.001051453 | -0.076903995 |
| CCL2 | -1.288180858 | 9.92473694 | -3.894044013 | 0.00022837 | 0.00110096 | -0.129419447 |
| THBS1 | -1.259602199 | 10.80702517 | -3.820094429 | 0.000292515 | 0.001361382 | -0.364349789 |
| ACSM2A | -1.153084434 | 6.757957112 | -3.812518724 | 0.000299985 | 0.001393045 | -0.388254514 |
| CXCL8 | -1.43367324 | 8.095559426 | -3.800914784 | 0.000311781 | 0.001439655 | -0.424810935 |
| FGA | -1.11958111 | 12.82288404 | -3.769522921 | 0.00034596 | 0.001576971 | -0.523345233 |
| CCN1 | -1.294339923 | 9.184118784 | -3.751362963 | 0.000367338 | 0.001661941 | -0.580104329 |
| CPS1 | -1.02731141 | 12.2473429 | -3.735334796 | 0.000387251 | 0.001734932 | -0.630051908 |
| SNORA14A | -1.169691155 | 10.87973709 | -3.705765203 | 0.000426722 | 0.001890616 | -0.72182962 |
| AGMO | -1.027195015 | 9.871512864 | -3.701621672 | 0.000432551 | 0.001912389 | -0.734651908 |
| NR1H4 | -1.161753516 | 8.833298861 | -3.701133362 | 0.000433243 | 0.001914548 | -0.736162373 |
| MYRIP | -1.089915169 | 7.744892956 | -3.698056418 | 0.000437627 | 0.001930296 | -0.74567712 |
| SLCO1B3 | -2.284602643 | 8.565508632 | -3.694966475 | 0.000442072 | 0.001946254 | -0.755226804 |
| ART4 | -1.333965596 | 7.355233744 | -3.680079032 | 0.0004641 | 0.002028993 | -0.801163452 |
| FGL1 | -1.272751121 | 11.28376871 | -3.676031161 | 0.000470267 | 0.00205167 | -0.8136323 |
| FMO5 | -1.159637133 | 10.62322583 | -3.644157144 | 0.00052164 | 0.002244579 | -0.911496126 |
| APOC3 | -1.12518463 | 12.87811339 | -3.623963271 | 0.000556908 | 0.002376233 | -0.973203142 |
| UGT3A1 | -1.239441297 | 7.968190877 | -3.619892755 | 0.000564287 | 0.002405536 | -0.985613672 |
| GC | -1.183853432 | 12.33455121 | -3.575967159 | 0.000650063 | 0.002720062 | -1.118938611 |
| OSMR | -1.243635724 | 8.530623506 | -3.552788966 | 0.000700184 | 0.002896935 | -1.188844813 |
| PLG | -1.186993452 | 12.65058302 | -3.545647603 | 0.000716352 | 0.002952148 | -1.210320932 |
| ETNK2 | -1.020182832 | 10.25456499 | -3.538600381 | 0.000732654 | 0.003010103 | -1.231484979 |
| CCL21 | -1.492195003 | 9.011717926 | -3.508783365 | 0.000805608 | 0.003264931 | -1.320710921 |
| IGFBP1 | -1.088821413 | 12.52205051 | -3.462132159 | 0.000933726 | 0.003709872 | -1.459265492 |
| SLC25A27 | -1.000095348 | 7.383214885 | -3.443493528 | 0.000990123 | 0.003901824 | -1.514261851 |
| ITIH1 | -1.021929339 | 12.21281562 | -3.390840309 | 0.001167365 | 0.004506103 | -1.668498359 |
| S100A8 | -1.308343514 | 8.097157949 | -3.326639852 | 0.001424072 | 0.005346592 | -1.854280226 |
| PDK4 | -1.062293107 | 10.33651767 | -3.308913455 | 0.001503819 | 0.005611728 | -1.905129286 |
| IFI44L | -1.085943004 | 7.52058432 | -3.243934822 | 0.001833554 | 0.006658173 | -2.089845297 |
| DEFB1 | -1.034004742 | 6.410424368 | -3.201807759 | 0.002082409 | 0.007435596 | -2.208174616 |
| ACOT12 | -1.11696167 | 8.647556182 | -3.16841295 | 0.002301824 | 0.008102231 | -2.301168473 |
| S100A12 | -1.106592901 | 6.234600781 | -3.146779739 | 0.00245532 | 0.008552949 | -2.361025339 |
| C7 | -1.813608649 | 9.976821781 | -3.08472123 | 0.002950431 | 0.010017539 | -2.531038256 |
| MIR21 | -1.136766165 | 8.391551958 | -2.89754914 | 0.005064545 | 0.015839115 | -3.028193664 |
| G6PC | -1.062793116 | 12.01116107 | -2.724808582 | 0.008182718 | 0.023781183 | -3.465434517 |
| DCN | -1.352149177 | 9.194210282 | -2.660495833 | 0.009736332 | 0.027424051 | -3.622741843 |
| CYP2E1 | -1.17532846 | 12.72618172 | -2.54139754 | 0.013340136 | 0.03577582 | -3.905976212 |
| CHI3L1 | -1.210343305 | 8.244051578 | -2.442233194 | 0.017217123 | 0.04408155 | -4.133628438 |
