# Supplementary material for: A Comprehensive Genomic Analysis Constructs miRNA–mRNA Interaction Network in Hepatoblastoma
Source: Front Cell Dev Biol. 2021 Aug 6;9:655703. doi: 10.3389/fcell.2021.655703 (PMC8377242; doi:10.3389/fcell.2021.655703)
Supplement: Supplementary file 13 [file Table_10.DOCX]

**Table S10. The intersection of downregulated DE-mRNAs and potential target genes of upregulated hub miRNAs.**

| **Entrez ID** | **Gene symbol** | **Gene title** | **logFC** | **P.Value** | **adj.P.Val** |
| --- | --- | --- | --- | --- | --- |
| 3290 | HSD11B1 | Hydroxysteroid 11-Beta Dehydrogenase 1 | -3.936982 | 2.44E-14 | 2.71E-12 |
| 8647 | ABCB11 | ATP Binding Cassette Subfamily B Member 11 | -3.866466 | 9.14E-11 | 3.07E-09 |
| 6505 | SLC1A1 | Solute Carrier Family 1 Member 1 | -3.838464 | 5.79E-17 | 1.38E-14 |
| 8876 | VNN1 | Vanin 1 | -3.827558 | 3.84E-10 | 1.08E-08 |
| 5444 | PON1 | Paraoxonase 1 | -3.657037 | 1.30E-10 | 4.19E-09 |
| 7498 | XDH | Xanthine Dehydrogenase | -3.450098 | 1.03E-20 | 7.20E-18 |
| 3026 | HABP2 | Hyaluronan Binding Protein 2 | -3.445562 | 8.34E-14 | 8.09E-12 |
| 4837 | NNMT | Nicotinamide N-Methyltransferase | -3.427393 | 2.54E-15 | 3.65E-13 |
| 27165 | GLS2 | Glutaminase 2 | -3.405992 | 2.31E-26 | 1.09E-22 |
| 1356 | CP | Ceruloplasmin | -3.315976 | 1.01E-08 | 1.86E-07 |
| 9615 | GDA | Guanine Deaminase | -3.287679 | 5.61E-14 | 5.77E-12 |
| 729 | C6 | Complement C6 | -3.234766 | 2.80E-12 | 1.63E-10 |
| 27232 | GNMT | Glycine N-Methyltransferase | -3.048366 | 6.64E-16 | 1.15E-13 |
| 325 | APCS | APC Regulator Of WNT Signaling Pathway | -2.952925 | 5.26E-06 | 4.14E-05 |
| 2690 | GHR | Growth Hormone Receptor | -2.810463 | 2.74E-11 | 1.12E-09 |
| 10135 | NAMPT | Nicotinamide Phosphoribosyltransferase | -2.669674 | 6.89E-14 | 6.95E-12 |
| 5054 | SERPINE1 | Serpin Family E Member 1 | -2.659203 | 5.15E-11 | 1.91E-09 |
| 4496 | MT1H | Metallothionein 1H | -2.541173 | 6.85E-13 | 5.08E-11 |
| 114770 | PGLYRP2 | Peptidoglycan Recognition Protein 2 | -2.517689 | 2.78E-13 | 2.31E-11 |
| 2920 | CXCL2 | C-X-C Motif Chemokine Ligand 2 | -2.517158 | 1.31E-14 | 1.59E-12 |
| 3263 | HPX | Hemopexin | -2.499737 | 3.55E-10 | 1.00E-08 |
| 760 | CA2 | Carbonic Anhydrase 2 | -2.484649 | 4.73E-20 | 2.87E-17 |
| 6590 | SLPI | Secretory Leukocyte Peptidase Inhibitor | -2.401008 | 2.27E-11 | 9.61E-10 |
| 27302 | BMP10 | Bone Morphogenetic Protein 10 | -2.398134 | 8.72E-13 | 6.13E-11 |
| 13 | AADAC | Arylacetamide Deacetylase | -2.357572 | 1.95E-05 | 0.00013 |
| 1582 | CYP8B1 | Cytochrome P450 Family 8 Subfamily B Member 1 | -2.320667 | 1.22E-07 | 1.57E-06 |
| 3242 | HPD | 4-Hydroxyphenylpyruvate Dioxygenase | -2.316489 | 3.99E-07 | 4.36E-06 |
| 64816 | CYP3A43 | Cytochrome P450 Family 3 Subfamily A Member 43 | -2.266173 | 2.44E-06 | 2.15E-05 |
| 5004 | ORM1 | Orosomucoid 1 | -2.26306 | 3.94E-08 | 5.99E-07 |
| 8671 | SLC4A4 | Solute Carrier Family 4 Member 4 | -2.253451 | 5.82E-08 | 8.38E-07 |
| 715 | C1R | Complement C1r | -2.214094 | 2.91E-10 | 8.50E-09 |
| 1577 | CYP3A5 | Cytochrome P450 Family 3 Subfamily A Member 5 | -2.209443 | 4.09E-08 | 6.17E-07 |
| 11343 | MGLL | Monoglyceride Lipase | -2.199274 | 4.59E-22 | 5.39E-19 |
| 22865 | SLITRK3 | SLIT And NTRK Like Family Member 3 | -2.198895 | 1.57E-13 | 1.41E-11 |
| 10216 | PRG4 | Proteoglycan 4 | -2.18173 | 1.43E-08 | 2.48E-07 |
| 154661 | RUNDC3B | RUN Domain Containing 3B | -2.106201 | 1.74E-16 | 3.49E-14 |
| 10221 | TRIB1 | Tribbles Pseudokinase 1 | -2.096586 | 9.50E-18 | 2.71E-15 |
| 79974 | CPED1 | Cadherin Like And PC-Esterase Domain Containing 1 | -2.096572 | 1.02E-06 | 9.98E-06 |
| 4501 | MT1X | Metallothionein 1X | -2.080952 | 4.32E-11 | 1.65E-09 |
| 732 | C8B | Complement C8 Beta Chain | -2.077098 | 4.48E-12 | 2.40E-10 |
| 390 | RND3 | Rho Family GTPase 3 | -2.072477 | 1.75E-11 | 7.72E-10 |
| 7360 | UGP2 | UDP-Glucose Pyrophosphorylase 2 | -2.070843 | 1.23E-12 | 7.90E-11 |
| 8424 | BBOX1 | Gamma-Butyrobetaine Hydroxylase 1 | -2.066137 | 3.34E-17 | 8.26E-15 |
| 6648 | SOD2 | Superoxide Dismutase 2 | -2.050926 | 3.03E-11 | 1.21E-09 |
| 64399 | HHIP | Hedgehog Interacting Protein | -2.049972 | 2.01E-08 | 3.31E-07 |
| 3977 | LIFR | LIF Receptor Subunit Alpha | -2.04584 | 1.88E-06 | 1.71E-05 |
| 3638 | INSIG1 | Insulin Induced Gene 1 | -2.034934 | 9.68E-14 | 9.20E-12 |
| 3075 | CFH | Complement Factor H | -1.983635 | 1.73E-06 | 1.58E-05 |
| 8835 | SOCS2 | Suppressor Of Cytokine Signaling 2 | -1.970345 | 6.34E-19 | 2.49E-16 |
| 23541 | SEC14L2 | SEC14 Like Lipid Binding 2 | -1.95525 | 1.19E-12 | 7.71E-11 |
| 23491 | CES3 | Carboxylesterase 3 | -1.952692 | 1.25E-10 | 4.02E-09 |
| 5446 | PON3 | Paraoxonase 3 | -1.945249 | 1.22E-11 | 5.70E-10 |
| 2674 | GFRA1 | GDNF Family Receptor Alpha 1 | -1.944059 | 7.58E-10 | 1.98E-08 |
| 5009 | OTC | Ornithine Transcarbamylase | -1.926045 | 2.77E-06 | 2.39E-05 |
| 55664 | CDC37L1 | Cell Division Cycle 37 Like 1 | -1.925179 | 1.95E-13 | 1.71E-11 |
| 7113 | TMPRSS2 | Transmembrane Serine Protease 2 | -1.919015 | 1.06E-12 | 7.01E-11 |
| 1071 | CETP | Cholesteryl Ester Transfer Protein | -1.909451 | 1.91E-11 | 8.24E-10 |
| 7538 | ZFP36 | ZFP36 Ring Finger Protein | -1.888806 | 1.67E-11 | 7.43E-10 |
| 1543 | CYP1A1 | Cytochrome P450 Family 1 Subfamily A Member 1 | -1.868267 | 1.41E-06 | 1.32E-05 |
| 6342 | SCP2 | Sterol Carrier Protein 2 | -1.864827 | 1.79E-11 | 7.86E-10 |
| 635 | BHMT | Betaine--Homocysteine S-Methyltransferase | -1.850261 | 4.34E-07 | 4.70E-06 |
| 9053 | MAP7 | Microtubule Associated Protein 7 | -1.833283 | 2.17E-16 | 4.26E-14 |
| 2353 | FOS | Fos Proto-Oncogene, AP-1 Transcription Factor Subunit | -1.816576 | 6.57E-06 | 5.03E-05 |
| 3084 | NRG1 | Neuregulin 1 | -1.813585 | 2.99E-15 | 4.24E-13 |
| 5743 | PTGS2 | Prostaglandin-Endoperoxide Synthase 2 | -1.792513 | 1.27E-09 | 3.13E-08 |
| 2805 | GOT1 | Glutamic-Oxaloacetic Transaminase 1 | -1.78853 | 2.45E-14 | 2.71E-12 |
| 260293 | CYP4X1 | Cytochrome P450 Family 4 Subfamily X Member 1 | -1.78228 | 3.23E-08 | 5.00E-07 |
| 722 | C4BPA | Complement Component 4 Binding Protein Alpha | -1.764003 | 1.04E-05 | 7.48E-05 |
| 25987 | TSKU | Tsukushi, Small Leucine Rich Proteoglycan | -1.756694 | 5.09E-13 | 3.88E-11 |
| 4886 | NPY1R | Neuropeptide Y Receptor Y1 | -1.717678 | 2.41E-07 | 2.83E-06 |
| 2259 | FGF14 | Fibroblast Growth Factor 14 | -1.709054 | 2.12E-10 | 6.45E-09 |
| 6799 | SULT1A2 | Sulfotransferase Family 1A Member 2 | -1.699002 | 4.10E-14 | 4.33E-12 |
| 6470 | SHMT1 | Serine Hydroxymethyltransferase 1 | -1.698571 | 6.75E-11 | 2.37E-09 |
| 1958 | EGR1 | Early Growth Response 1 | -1.698506 | 7.76E-08 | 1.07E-06 |
| 360 | AQP3 | Aquaporin 3 | -1.672081 | 1.75E-10 | 5.48E-09 |
| 6385 | SDC4 | Syndecan 4 | -1.669884 | 7.42E-11 | 2.57E-09 |
| 4494 | MT1F | Metallothionein 1F | -1.667852 | 7.79E-06 | 5.81E-05 |
| 9547 | CXCL14 | C-X-C Motif Chemokine Ligand 14 | -1.667635 | 4.86E-06 | 3.87E-05 |
| 5106 | PCK2 | Phosphoenolpyruvate Carboxykinase 2, Mitochondrial | -1.664162 | 1.92E-11 | 8.25E-10 |
| 330 | BIRC3 | Baculoviral IAP Repeat Containing 3 | -1.655151 | 6.20E-08 | 8.82E-07 |
| 3626 | INHBC | Inhibin Subunit Beta C | -1.651417 | 5.34E-07 | 5.64E-06 |
| 1066 | CES1 | Carboxylesterase 1 | -1.641968 | 3.86E-06 | 3.17E-05 |
| 2628 | GATM | Glycine Amidinotransferase | -1.638369 | 3.48E-05 | 0.000214 |
| 64902 | AGXT2 | Alanine--Glyoxylate Aminotransferase 2 | -1.633393 | 9.29E-08 | 1.24E-06 |
| 57561 | ARRDC3 | Arrestin Domain Containing 3 | -1.625773 | 6.20E-07 | 6.44E-06 |
| 3479 | IGF1 | Insulin Like Growth Factor 1 | -1.612198 | 1.63E-07 | 2.03E-06 |
| 5348 | FXYD1 | FXYD Domain Containing Ion Transport Regulator 1 | -1.601353 | 3.98E-09 | 8.37E-08 |
| 1368 | CPM | Carboxypeptidase M | -1.595344 | 1.62E-10 | 5.10E-09 |
| 9185 | REPS2 | RALBP1 Associated Eps Domain Containing 2 | -1.590833 | 2.94E-13 | 2.41E-11 |
| 12 | SERPINA3 | Serpin Family A Member 3 | -1.58864 | 8.08E-07 | 8.08E-06 |
| 64332 | NFKBIZ | NFKB Inhibitor Zeta | -1.588024 | 4.88E-14 | 5.07E-12 |
| 6542 | SLC7A2 | Solute Carrier Family 7 Member 2 | -1.586271 | 5.12E-14 | 5.29E-12 |
| 2114 | ETS2 | ETS Proto-Oncogene 2, Transcription Factor | -1.572304 | 3.98E-15 | 5.47E-13 |
| 5873 | RAB27A | RAB27A, Member RAS Oncogene Family | -1.555398 | 3.80E-13 | 3.02E-11 |
| 2266 | FGG | Fibrinogen Gamma Chain | -1.552004 | 3.71E-05 | 0.000226 |
| 10555 | AGPAT2 | 1-Acylglycerol-3-Phosphate O-Acyltransferase 2 | -1.537627 | 4.14E-10 | 1.15E-08 |
| 8553 | BHLHE40 | Basic Helix-Loop-Helix Family Member E40 | -1.53639 | 5.97E-09 | 1.18E-07 |
| 462 | SERPINC1 | Serpin Family C Member 1 | -1.531195 | 6.98E-06 | 5.29E-05 |
| 1843 | DUSP1 | Dual Specificity Phosphatase 1 | -1.524848 | 2.91E-07 | 3.29E-06 |
| 2099 | ESR1 | Estrogen Receptor 1 | -1.512812 | 5.40E-15 | 7.16E-13 |
| 166929 | SGMS2 | Sphingomyelin Synthase 2 | -1.502602 | 4.52E-08 | 6.67E-07 |
| 143941 | TTC36 | Tetratricopeptide Repeat Domain 36 | -1.493401 | 1.49E-21 | 1.34E-18 |
| 6366 | CCL21 | C-C Motif Chemokine Ligand 21 | -1.492195 | 0.000806 | 0.003265 |
| 90634 | N4BP2L1 | NEDD4 Binding Protein 2 Like 1 | -1.489788 | 1.04E-12 | 6.92E-11 |
| 64651 | CSRNP1 | Cysteine And Serine Rich Nuclear Protein 1 | -1.487534 | 1.52E-11 | 6.86E-10 |
| 121512 | FGD4 | FYVE, RhoGEF And PH Domain Containing 4 | -1.485942 | 3.77E-09 | 8.02E-08 |
| 4493 | MT1E | Metallothionein 1E | -1.483886 | 3.03E-08 | 4.73E-07 |
| 201895 | SMIM14 | Small Integral Membrane Protein 14 | -1.476455 | 4.65E-14 | 4.89E-12 |
| 55526 | DHTKD1 | Dehydrogenase E1 And Transketolase Domain Containing 1 | -1.462718 | 6.23E-13 | 4.65E-11 |
| 4430 | MYO1B | Myosin IB | -1.456999 | 3.05E-08 | 4.76E-07 |
| 9099 | USP2 | Ubiquitin Specific Peptidase 2 | -1.448048 | 2.21E-14 | 2.48E-12 |
| 6715 | SRD5A1 | Steroid 5 Alpha-Reductase 1 | -1.447426 | 1.40E-21 | 1.31E-18 |
| 11057 | ABHD2 | Abhydrolase Domain Containing 2, Acylglycerol Lipase | -1.439381 | 6.32E-11 | 2.24E-09 |
| 8801 | SUCLG2 | Succinate-CoA Ligase GDP-Forming Subunit Beta | -1.437744 | 3.34E-13 | 2.71E-11 |
| 7433 | VIPR1 | Vasoactive Intestinal Peptide Receptor 1 | -1.43483 | 5.28E-11 | 1.95E-09 |
| 3576 | CXCL8 | C-X-C Motif Chemokine Ligand 8 | -1.433673 | 0.000312 | 0.00144 |
| 85453 | TSPYL5 | TSPY Like 5 | -1.428927 | 1.36E-07 | 1.72E-06 |
| 51422 | PRKAG2 | Protein Kinase AMP-Activated Non-Catalytic Subunit Gamma 2 | -1.428361 | 1.60E-14 | 1.87E-12 |
| 10157 | AASS | Aminoadipate-Semialdehyde Synthase | -1.427896 | 2.87E-10 | 8.44E-09 |
| 2329 | FMO4 | Flavin Containing Dimethylaniline Monoxygenase 4 | -1.417054 | 1.33E-07 | 1.70E-06 |
| 2244 | FGB | Fibrinogen Beta Chain | -1.415966 | 8.05E-05 | 0.000446 |
| 18 | ABAT | 4-Aminobutyrate Aminotransferase | -1.411843 | 1.35E-09 | 3.30E-08 |
| 10166 | SLC25A15 | Solute Carrier Family 25 Member 15 | -1.410887 | 6.74E-11 | 2.37E-09 |
| 5997 | RGS2 | Regulator Of G Protein Signaling 2 | -1.40952 | 3.77E-05 | 0.00023 |
| 23530 | NNT | Nicotinamide Nucleotide Transhydrogenase | -1.404728 | 3.21E-12 | 1.82E-10 |
| 4929 | NR4A2 | Nuclear Receptor Subfamily 4 Group A Member 2 | -1.404511 | 9.68E-06 | 7.04E-05 |
| 3383 | ICAM1 | Intercellular Adhesion Molecule 1 | -1.403921 | 3.78E-09 | 8.02E-08 |
| 94241 | TP53INP1 | Tumor Protein P53 Inducible Nuclear Protein 1 | -1.395954 | 6.71E-08 | 9.46E-07 |
| 1592 | CYP26A1 | Cytochrome P450 Family 26 Subfamily A Member 1 | -1.394588 | 3.44E-13 | 2.77E-11 |
| 1827 | RCAN1 | Regulator Of Calcineurin 1 | -1.394532 | 2.64E-14 | 2.91E-12 |
| 4217 | MAP3K5 | Mitogen-Activated Protein Kinase Kinase Kinase 5 | -1.391992 | 2.62E-09 | 5.88E-08 |
| 1910 | EDNRB | Endothelin Receptor Type B | -1.391017 | 9.43E-08 | 1.26E-06 |
| 9510 | ADAMTS1 | ADAM Metallopeptidase With Thrombospondin Type 1 Motif 1 | -1.389184 | 1.78E-06 | 1.61E-05 |
| 3726 | JUNB | JunB Proto-Oncogene, AP-1 Transcription Factor Subunit | -1.377879 | 1.54E-07 | 1.93E-06 |
| 3426 | CFI | Complement Factor I | -1.369182 | 5.17E-07 | 5.48E-06 |
| 36 | ACADSB | Acyl-CoA Dehydrogenase Short/Branched Chain | -1.36811 | 1.43E-06 | 1.34E-05 |
| 57683 | ZDBF2 | Zinc Finger DBF-Type Containing 2 | -1.364869 | 4.20E-08 | 6.31E-07 |
| 59277 | NTN4 | Netrin 4 | -1.355894 | 4.09E-12 | 2.24E-10 |
| 1634 | DCN | Decorin | -1.352149 | 0.009736 | 0.027424 |
| 51361 | HOOK1 | Hook Microtubule Tethering Protein 1 | -1.347243 | 1.42E-06 | 1.33E-05 |
| 54206 | ERRFI1 | ERBB Receptor Feedback Inhibitor 1 | -1.346218 | 1.18E-05 | 8.37E-05 |
| 6304 | SATB1 | SATB Homeobox 1 | -1.344668 | 7.09E-07 | 7.22E-06 |
| 4170 | MCL1 | MCL1 Apoptosis Regulator, BCL2 Family Member | -1.335647 | 1.76E-10 | 5.50E-09 |
| 9590 | AKAP12 | A-Kinase Anchoring Protein 12 | -1.331145 | 2.46E-07 | 2.87E-06 |
| 1847 | DUSP5 | Dual Specificity Phosphatase 5 | -1.327926 | 2.20E-12 | 1.33E-10 |
| 847 | CAT | Catalase | -1.320071 | 1.83E-09 | 4.28E-08 |
| 25976 | TIPARP | TCDD Inducible Poly(ADP-Ribose) Polymerase | -1.312986 | 5.36E-09 | 1.09E-07 |
| 2730 | GCLM | Glutamate-Cysteine Ligase Modifier Subunit | -1.303805 | 5.29E-11 | 1.95E-09 |
| 4306 | NR3C2 | Nuclear Receptor Subfamily 3 Group C Member 2 | -1.298438 | 2.11E-13 | 1.83E-11 |
| 4781 | NFIB | Nuclear Factor I B | -1.289459 | 3.60E-05 | 0.00022 |
| 6347 | CCL2 | C-C Motif Chemokine Ligand 2 | -1.288181 | 0.000228 | 0.001101 |
| 2634 | GBP2 | Guanylate Binding Protein 2 | -1.288118 | 6.07E-06 | 4.69E-05 |
| 687 | KLF9 | Kruppel Like Factor 9 | -1.28489 | 2.15E-12 | 1.30E-10 |
| 1573 | CYP2J2 | Cytochrome P450 Family 2 Subfamily J Member 2 | -1.276275 | 1.04E-06 | 1.01E-05 |
| 57600 | FNIP2 | Folliculin Interacting Protein 2 | -1.273533 | 1.40E-08 | 2.45E-07 |
| 2267 | FGL1 | Fibrinogen Like 1 | -1.272751 | 0.00047 | 0.002052 |
| 2632 | GBE1 | 1,4-Alpha-Glucan Branching Enzyme 1 | -1.271194 | 1.21E-09 | 3.00E-08 |
| 6574 | SLC20A1 | Solute Carrier Family 20 Member 1 | -1.267519 | 5.97E-09 | 1.18E-07 |
| 83758 | RBP5 | Retinol Binding Protein 5 | -1.267146 | 1.14E-08 | 2.05E-07 |
| 5272 | SERPINB9 | Serpin Family B Member 9 | -1.262618 | 1.85E-07 | 2.25E-06 |
| 7057 | THBS1 | Thrombospondin 1 | -1.259602 | 0.000293 | 0.001361 |
| 91614 | DEPDC7 | DEP Domain Containing 7 | -1.258348 | 1.50E-08 | 2.58E-07 |
| 10150 | MBNL2 | Muscleblind Like Splicing Regulator 2 | -1.256902 | 3.50E-07 | 3.89E-06 |
| 6095 | RORA | RAR Related Orphan Receptor A | -1.250873 | 3.52E-06 | 2.93E-05 |
| 1349 | COX7B | Cytochrome C Oxidase Subunit 7B | -1.248412 | 5.42E-09 | 1.09E-07 |
| 22849 | CPEB3 | Cytoplasmic Polyadenylation Element Binding Protein 3 | -1.244688 | 1.38E-13 | 1.26E-11 |
| 8850 | KAT2B | Lysine Acetyltransferase 2B | -1.240185 | 3.85E-06 | 3.16E-05 |
| 3553 | IL1B | Interleukin 1 Beta | -1.235904 | 4.77E-07 | 5.12E-06 |
| 8858 | PROZ | Protein Z, Vitamin K Dependent Plasma Glycoprotein | -1.234475 | 1.19E-05 | 8.37E-05 |
| 1317 | SLC31A1 | Solute Carrier Family 31 Member 1 | -1.222901 | 7.05E-13 | 5.14E-11 |
| 57688 | ZSWIM6 | Zinc Finger SWIM-Type Containing 6 | -1.221254 | 8.72E-08 | 1.19E-06 |
| 132299 | OCIAD2 | OCIA Domain Containing 2 | -1.217979 | 1.81E-08 | 3.03E-07 |
| 3566 | IL4R | Interleukin 4 Receptor | -1.216104 | 3.46E-12 | 1.93E-10 |
| 9674 | KIAA0040 | KIAA0040 | -1.207572 | 4.49E-06 | 3.61E-05 |
| 10449 | ACAA2 | Acetyl-CoA Acyltransferase 2 | -1.204138 | 2.43E-13 | 2.05E-11 |
| 1519 | CTSO | Cathepsin O | -1.197586 | 3.08E-06 | 2.61E-05 |
| 23043 | TNIK | TRAF2 And NCK Interacting Kinase | -1.197385 | 5.67E-09 | 1.14E-07 |
| 7276 | TTR | Transthyretin | -1.197371 | 1.36E-06 | 1.28E-05 |
| 10580 | SORBS1 | Sorbin And SH3 Domain Containing 1 | -1.195341 | 1.19E-06 | 1.15E-05 |
| 1528 | CYB5A | Cytochrome B5 Type A | -1.190826 | 1.76E-08 | 2.97E-07 |
| 8013 | NR4A3 | Nuclear Receptor Subfamily 4 Group A Member 3 | -1.188027 | 7.67E-07 | 7.70E-06 |
| 28970 | C11orf54 | Chromosome 11 Open Reading Frame 54 | -1.187999 | 4.24E-08 | 6.35E-07 |
| 28513 | CDH19 | Cadherin 19 | -1.179679 | 2.66E-06 | 2.31E-05 |
| 1604 | CD55 | CD55 Molecule | -1.175163 | 1.76E-06 | 1.60E-05 |
| 2823 | GPM6A | Glycoprotein M6A | -1.173498 | 4.31E-09 | 8.93E-08 |
| 3899 | AFF3 | AF4/FMR2 Family Member 3 | -1.165129 | 1.64E-09 | 3.90E-08 |
| 23710 | GABARAPL1 | GABA Type A Receptor Associated Protein Like 1 | -1.16129 | 1.31E-08 | 2.31E-07 |
| 3569 | IL6 | Interleukin 6 | -1.157593 | 2.31E-06 | 2.05E-05 |
| 4502 | MT2A | Metallothionein 2A | -1.153574 | 2.26E-07 | 2.67E-06 |
| 3699 | ITIH3 | Inter-Alpha-Trypsin Inhibitor Heavy Chain 3 | -1.148671 | 0.000136 | 0.000707 |
| 7164 | TPD52L1 | TPD52 Like 1 | -1.148438 | 4.93E-12 | 2.61E-10 |
| 401474 | SAMD12 | Sterile Alpha Motif Domain Containing 12 | -1.146467 | 3.64E-07 | 4.02E-06 |
| 467 | ATF3 | Activating Transcription Factor 3 | -1.142735 | 1.03E-08 | 1.89E-07 |
| 4163 | MCC | MCC Regulator Of WNT Signaling Pathway | -1.139661 | 1.30E-11 | 5.97E-10 |
| 1666 | DECR1 | 2,4-Dienoyl-CoA Reductase 1 | -1.138969 | 1.03E-11 | 4.91E-10 |
| 54210 | TREM1 | Triggering Receptor Expressed On Myeloid Cells 1 | -1.137826 | 1.58E-06 | 1.46E-05 |
| 645745 | MT1HL1 | Metallothionein 1H Like 1 | -1.134551 | 5.01E-11 | 1.87E-09 |
| 5627 | PROS1 | Protein S | -1.133434 | 7.65E-07 | 7.68E-06 |
| 9689 | BZW1 | Basic Leucine Zipper And W2 Domains 1 | -1.131356 | 9.72E-08 | 1.29E-06 |
| 80315 | CPEB4 | Cytoplasmic Polyadenylation Element Binding Protein 4 | -1.130354 | 1.32E-05 | 9.18E-05 |
| 85379 | KIAA1671 | KIAA1671 | -1.130267 | 7.24E-17 | 1.68E-14 |
| 60468 | BACH2 | BTB Domain And CNC Homolog 2 | -1.127646 | 8.07E-12 | 4.00E-10 |
| 3164 | NR4A1 | Nuclear Receptor Subfamily 4 Group A Member 1 | -1.126861 | 1.88E-05 | 0.000125 |
| 57630 | SH3RF1 | SH3 Domain Containing Ring Finger 1 | -1.123359 | 1.29E-09 | 3.16E-08 |
| 58488 | PCTP | Phosphatidylcholine Transfer Protein | -1.110869 | 5.83E-09 | 1.16E-07 |
| 9695 | EDEM1 | ER Degradation Enhancing Alpha-Mannosidase Like Protein 1 | -1.109914 | 6.46E-10 | 1.72E-08 |
| 7098 | TLR3 | Toll Like Receptor 3 | -1.105265 | 5.87E-05 | 0.000338 |
| 55638 | SYBU | Syntabulin | -1.100629 | 6.22E-05 | 0.000356 |
| 217 | ALDH2 | Aldehyde Dehydrogenase 2 Family Member | -1.099809 | 1.39E-08 | 2.43E-07 |
| 3486 | IGFBP3 | Insulin Like Growth Factor Binding Protein 3 | -1.098822 | 3.17E-06 | 2.68E-05 |
| 4329 | ALDH6A1 | Aldehyde Dehydrogenase 6 Family Member A1 | -1.096657 | 1.95E-08 | 3.22E-07 |
| 54103 | GSAP | Gamma-Secretase Activating Protein | -1.091324 | 3.71E-06 | 3.06E-05 |
| 25897 | RNF19A | Ring Finger Protein 19A, RBR E3 Ubiquitin Protein Ligase | -1.088324 | 3.65E-06 | 3.02E-05 |
| 4257 | MGST1 | Microsomal Glutathione S-Transferase 1 | -1.088135 | 1.61E-06 | 1.49E-05 |
| 10964 | IFI44L | Interferon Induced Protein 44 Like | -1.085943 | 0.001834 | 0.006658 |
| 54414 | SIAE | Sialic Acid Acetylesterase | -1.079491 | 1.37E-08 | 2.40E-07 |
| 23365 | ARHGEF12 | Rho Guanine Nucleotide Exchange Factor 12 | -1.074311 | 1.38E-06 | 1.29E-05 |
| 49854 | ZBTB21 | Zinc Finger And BTB Domain Containing 21 | -1.074183 | 1.20E-07 | 1.56E-06 |
| 57568 | SIPA1L2 | Signal Induced Proliferation Associated 1 Like 2 | -1.07085 | 6.65E-07 | 6.83E-06 |
| 6446 | SGK1 | Serum/Glucocorticoid Regulated Kinase 1 | -1.069414 | 3.05E-07 | 3.43E-06 |
| 166785 | MMAA | Metabolism Of Cobalamin Associated A | -1.069249 | 1.82E-08 | 3.04E-07 |
| 2729 | GCLC | Glutamate-Cysteine Ligase Catalytic Subunit | -1.067899 | 1.11E-05 | 7.91E-05 |
| 4490 | MT1B | Metallothionein 1B | -1.065305 | 8.23E-12 | 4.07E-10 |
| 26040 | SETBP1 | SET Binding Protein 1 | -1.064397 | 2.43E-09 | 5.52E-08 |
| 5166 | PDK4 | Pyruvate Dehydrogenase Kinase 4 | -1.062293 | 0.001504 | 0.005612 |
| 2919 | CXCL1 | C-X-C Motif Chemokine Ligand 1 | -1.062184 | 2.68E-10 | 7.93E-09 |
| 10841 | FTCD | Formimidoyltransferase Cyclodeaminase | -1.060048 | 7.63E-08 | 1.06E-06 |
| 399665 | FAM102A | Family With Sequence Similarity 102 Member A | -1.055994 | 5.33E-09 | 1.08E-07 |
| 133 | ADM | Adrenomedullin | -1.054214 | 3.65E-09 | 7.81E-08 |
| 8519 | IFITM1 | Interferon Induced Transmembrane Protein 1 | -1.0529 | 3.12E-05 | 0.000195 |
| 7763 | ZFAND5 | Zinc Finger AN1-Type Containing 5 | -1.051682 | 2.49E-07 | 2.90E-06 |
| 1969 | EPHA2 | EPH Receptor A2 | -1.049996 | 4.07E-06 | 3.32E-05 |
| 491 | ATP2B2 | ATPase Plasma Membrane Ca2+ Transporting 2 | -1.041903 | 5.63E-07 | 5.90E-06 |
| 3597 | IL13RA1 | Interleukin 13 Receptor Subunit Alpha 1 | -1.040955 | 1.39E-07 | 1.75E-06 |
| 2034 | EPAS1 | Endothelial PAS Domain Protein 1 | -1.040194 | 5.37E-10 | 1.46E-08 |
| 1956 | EGFR | Epidermal Growth Factor Receptor | -1.038826 | 4.52E-09 | 9.29E-08 |
| 4774 | NFIA | Nuclear Factor I A | -1.037698 | 8.68E-05 | 0.000477 |
| 164832 | LONRF2 | LON Peptidase N-Terminal Domain And Ring Finger 2 | -1.0315 | 1.42E-09 | 3.45E-08 |
| 1520 | CTSS | Cathepsin S | -1.028707 | 1.42E-05 | 9.74E-05 |
| 1629 | DBT | Dihydrolipoamide Branched Chain Transacylase E2 | -1.02851 | 2.98E-07 | 3.36E-06 |
| 22822 | PHLDA1 | Pleckstrin Homology Like Domain Family A Member 1 | -1.028442 | 1.34E-07 | 1.70E-06 |
| 9076 | CLDN1 | Claudin 1 | -1.024301 | 1.33E-05 | 9.26E-05 |
| 2147 | F2 | Coagulation Factor II, Thrombin | -1.022893 | 6.31E-05 | 0.00036 |
| 219333 | USP12 | Ubiquitin Specific Peptidase 12 | -1.01957 | 7.22E-07 | 7.32E-06 |
| 26999 | CYFIP2 | Cytoplasmic FMR1 Interacting Protein 2 | -1.014321 | 1.39E-06 | 1.30E-05 |
| 27115 | PDE7B | Phosphodiesterase 7B | -1.00801 | 1.95E-07 | 2.35E-06 |
| 7378 | UPP1 | Uridine Phosphorylase 1 | -1.007056 | 1.44E-15 | 2.17E-13 |
| 6392 | SDHD | Succinate Dehydrogenase Complex Subunit D | -1.005468 | 1.02E-09 | 2.58E-08 |
| 6653 | SORL1 | Sortilin Related Receptor 1 | -1.003158 | 1.39E-06 | 1.31E-05 |
| 594 | BCKDHB | Branched Chain Keto Acid Dehydrogenase E1 Subunit Beta | -1.001151 | 1.77E-10 | 5.51E-09 |
