# Supplementary material for: A Comprehensive Genomic Analysis Constructs miRNA–mRNA Interaction Network in Hepatoblastoma
Source: Front Cell Dev Biol. 2021 Aug 6;9:655703. doi: 10.3389/fcell.2021.655703 (PMC8377242; doi:10.3389/fcell.2021.655703)
Supplement: Supplementary file 14 [file Table_11.DOCX]

**Table S11. The intersection of upregulated DE-mRNAs and potential target genes of downregulated hub miRNAs.**

| **Entrez ID** | **Gene symbol** | **Gene title** | **logFC** | **P.Value** | **adj.P.Val** |
| --- | --- | --- | --- | --- | --- |
| 23089 | PEG10 | Paternally Expressed 10 | 3.606445313 | 9.26E-21 | 6.97E-18 |
| 23657 | SLC7A11 | Solute Carrier Family 7 Member 11 | 3.486572778 | 8.05E-15 | 1.05E-12 |
| 1800 | DPEP1 | Dipeptidase 1 | 3.455693831 | 5.35E-13 | 4.06E-11 |
| 255743 | NPNT | Nephronectin | 3.282197308 | 9.85E-25 | 2.32E-21 |
| 10642 | IGF2BP1 | Insulin Like Growth Factor 2 MRNA Binding Protein 1 | 3.278136653 | 4.37E-21 | 3.43E-18 |
| 85407 | NKD1 | NKD Inhibitor Of WNT Signaling Pathway 1 | 3.145090499 | 1.58E-24 | 3.30E-21 |
| 51176 | LEF1 | Lymphoid Enhancer Binding Factor 1 | 3.017950221 | 2.87E-18 | 9.64E-16 |
| 64943 | NT5DC2 | 5'-Nucleotidase Domain Containing 2 | 2.700446625 | 8.94E-16 | 1.44E-13 |
| 4311 | MME | Membrane Metalloendopeptidase | 2.451658134 | 3.93E-08 | 5.98E-07 |
| 5347 | PLK1 | Polo Like Kinase 1 | 2.412514592 | 5.72E-11 | 2.07E-09 |
| 3655 | ITGA6 | Integrin Subunit Alpha | 2.350675736 | 8.99E-17 | 1.95E-14 |
| 7042 | TGFB2 | Transforming Growth Factor Beta 2 | 2.264112043 | 6.97E-11 | 2.43E-09 |
| 283659 | PRTG | Protogenin | 2.245048981 | 7.86E-10 | 2.04E-08 |
| 54894 | RNF43 | Ring Finger Protein 43 | 2.223383012 | 2.16E-16 | 4.26E-14 |
| 2182 | ACSL4 | Acyl-CoA Synthetase Long Chain Family Member 4 | 2.191079707 | 1.65E-10 | 5.15E-09 |
| 7153 | TOP2A | DNA Topoisomerase II Alpha | 2.178370882 | 1.57E-11 | 7.05E-10 |
| 1033 | CDKN3 | Cyclin Dependent Kinase Inhibitor 3 | 2.175037651 | 1.01E-12 | 6.79E-11 |
| 894 | CCND2 | Cyclin D2 | 2.155315522 | 1.11E-11 | 5.24E-10 |
| 389421 | LIN28B | Lin-28 Homolog B | 2.151232201 | 6.54E-07 | 6.74E-06 |
| 5274 | SERPINI1 | Serpin Family I Member 1 | 2.144525886 | 3.47E-08 | 5.33E-07 |
| 57127 | RHBG | Rh Family B Glycoprotein | 2.132733015 | 1.10E-11 | 5.22E-10 |
| 6091 | ROBO1 | Roundabout Guidance Receptor 1 | 2.131387277 | 1.25E-21 | 1.23E-18 |
| 983 | CDK1 | Cyclin Dependent Kinase 1 | 2.083547989 | 2.40E-11 | 9.97E-10 |
| 100288413 | ERVMER34-1 | Endogenous Retrovirus Group MER34 Member 1, Envelope | 2.071770702 | 1.12E-09 | 2.79E-08 |
| 28982 | FLVCR1 | FLVCR Heme Transporter 1 | 2.059286768 | 1.30E-14 | 1.58E-12 |
| 25805 | BAMBI | BMP And Activin Membrane Bound Inhibitor | 2.032911916 | 1.63E-20 | 1.10E-17 |
| 2305 | FOXM1 | Forkhead Box M1 | 2.024086663 | 1.01E-10 | 3.33E-09 |
| 10644 | IGF2BP2 | Insulin Like Growth Factor 2 MRNA Binding Protein 2 | 1.946770317 | 5.44E-19 | 2.18E-16 |
| 54443 | ANLN | Anillin Actin Binding Protein | 1.945267933 | 9.22E-11 | 3.09E-09 |
| 9543 | IGDCC3 | Immunoglobulin Superfamily DCC Subclass Member 3 | 1.94210042 | 1.97E-10 | 6.07E-09 |
| 8091 | HMGA2 | High Mobility Group AT-Hook 2 | 1.940571302 | 2.86E-13 | 2.36E-11 |
| 221150 | SKA3 | Spindle And Kinetochore Associated Complex Subunit 3 | 1.938216705 | 2.65E-10 | 7.84E-09 |
| 2882 | GPX7 | Glutathione Peroxidase 7 | 1.916614525 | 1.29E-14 | 1.58E-12 |
| 259266 | ASPM | Assembly Factor For Spindle Microtubules | 1.895340459 | 2.99E-10 | 8.69E-09 |
| 26022 | TMEM98 | Transmembrane Protein 98 | 1.846653982 | 2.87E-24 | 5.39E-21 |
| 5563 | PRKAA2 | Protein Kinase AMP-Activated Catalytic Subunit Alpha 2 | 1.836274412 | 6.73E-10 | 1.79E-08 |
| 55137 | FIGN | Fidgetin, Microtubule Severing Factor | 1.827531693 | 8.05E-11 | 2.75E-09 |
| 57643 | ZSWIM5 | Zinc Finger SWIM-Type Containing 5 | 1.811866754 | 6.34E-13 | 4.72E-11 |
| 9833 | MELK | Maternal Embryonic Leucine Zipper Kinase | 1.806620561 | 2.96E-12 | 1.71E-10 |
| 90381 | TICRR | TOPBP1 Interacting Checkpoint And Replication Regulator | 1.787516611 | 1.45E-10 | 4.59E-09 |
| 5019 | OXCT1 | 3-Oxoacid CoA-Transferase 1 | 1.771004551 | 3.92E-19 | 1.67E-16 |
| 55872 | PBK | PDZ Binding Kinase | 1.767009814 | 3.08E-10 | 8.90E-09 |
| 29127 | RACGAP1 | Rac GTPase Activating Protein 1 | 1.738577693 | 7.94E-17 | 1.78E-14 |
| 1063 | CENPF | Centromere Protein F | 1.727723425 | 1.15E-08 | 2.07E-07 |
| 9315 | NREP | Neuronal Regeneration Related Protein | 1.716951754 | 3.02E-11 | 1.21E-09 |
| 6790 | AURKA | Aurora Kinase A | 1.697446459 | 1.92E-10 | 5.96E-09 |
| 26049 | FAM169A | Family With Sequence Similarity 169 Member A | 1.689995381 | 6.82E-12 | 3.47E-10 |
| 26586 | CKAP2 | Cytoskeleton Associated Protein 2 | 1.681375739 | 1.46E-11 | 6.62E-10 |
| 3070 | HELLS | Helicase, Lymphoid Specific | 1.678850919 | 1.36E-10 | 4.36E-09 |
| 1462 | VCAN | Versican | 1.678252112 | 6.05E-05 | 0.000347235 |
| 891 | CCNB1 | Cyclin B1 | 1.675570182 | 1.61E-10 | 5.08E-09 |
| 84133 | ZNRF3 | Zinc And Ring Finger 3 | 1.669130819 | 1.34E-14 | 1.60E-12 |
| 72 | ACTG2 | Actin Gamma 2, Smooth Muscle | 1.656616796 | 2.32E-05 | 0.000150625 |
| 9057 | SLC7A6 | Solute Carrier Family 7 Member 6 | 1.655143258 | 1.29E-17 | 3.55E-15 |
| 5784 | PTPN14 | Protein Tyrosine Phosphatase Non-Receptor Type 14 | 1.64052097 | 1.74E-15 | 2.54E-13 |
| 55353 | LAPTM4B | Lysosomal Protein Transmembrane 4 Beta | 1.639573557 | 2.34E-10 | 7.03E-09 |
| 4288 | MKI67 | Marker Of Proliferation Ki-67 | 1.637800832 | 2.39E-11 | 9.95E-10 |
| 22974 | TPX2 | TPX2 Microtubule Nucleation Factor | 1.634283428 | 2.06E-08 | 3.39E-07 |
| 63920 | ZBED8 | Zinc Finger BED-Type Containing 8 | 1.626980376 | 4.61E-13 | 3.57E-11 |
| 81831 | NETO2 | Neuropilin And Tolloid Like 2 | 1.615814259 | 1.87E-08 | 3.10E-07 |
| 5754 | PTK7 | Protein Tyrosine Kinase 7 | 1.61061442 | 2.84E-11 | 1.15E-09 |
| 28951 | TRIB2 | Tribbles Pseudokinase 2 | 1.598725714 | 4.06E-19 | 1.67E-16 |
| 9787 | DLGAP5 | DLG Associated Protein 5 | 1.598257327 | 2.81E-08 | 4.43E-07 |
| 9415 | FADS2 | Fatty Acid Desaturase 2 | 1.565725669 | 1.11E-05 | 7.90E-05 |
| 3832 | KIF11 | Kinesin Family Member 11 | 1.554600523 | 7.17E-09 | 1.38E-07 |
| 2048 | EPHB2 | EPH Receptor B2 | 1.545548603 | 4.24E-13 | 3.33E-11 |
| 6241 | RRM2 | Ribonucleotide Reductase Regulatory Subunit M2 | 1.536782251 | 4.22E-10 | 1.17E-08 |
| 80055 | PGAP1 | Post-GPI Attachment To Proteins Inositol Deacylase 1 | 1.501023826 | 7.24E-09 | 1.39E-07 |
| 64151 | NCAPG | Non-SMC Condensin I Complex Subunit G | 1.493597014 | 2.01E-09 | 4.68E-08 |
| 1111 | CHEK1 | Checkpoint Kinase 1 | 1.489541296 | 1.46E-10 | 4.62E-09 |
| 7516 | XRCC2 | X-Ray Repair Cross Complementing 2 | 1.484714456 | 1.06E-08 | 1.95E-07 |
| 5324 | PLAG1 | PLAG1 Zinc Finger | 1.477146037 | 5.59E-11 | 2.04E-09 |
| 55215 | FANCI | FA Complementation Group I | 1.474634584 | 3.36E-11 | 1.33E-09 |
| 9918 | NCAPD2 | Non-SMC Condensin I Complex Subunit D2 | 1.469891557 | 2.71E-13 | 2.26E-11 |
| 30811 | HUNK | Hormonally Up-Regulated Neu-Associated Kinase | 1.463194082 | 2.68E-10 | 7.92E-09 |
| 1875 | E2F5 | E2F Transcription Factor 5 | 1.452304495 | 6.01E-10 | 1.62E-08 |
| 7552 | ZNF711 | Zinc Finger Protein 711 | 1.443436117 | 2.67E-09 | 5.96E-08 |
| 2702 | GJA5 | Gap Junction Protein Alpha 5 | 1.438558726 | 6.13E-12 | 3.16E-10 |
| 139189 | DGKK | Diacylglycerol Kinase Kappa | 1.426002402 | 2.45E-07 | 2.86E-06 |
| 8404 | SPARCL1 | SPARC Like 1 | 1.423824003 | 0.000319642 | 0.001473063 |
| 440193 | CCDC88C | Coiled-Coil Domain Containing 88C | 1.416030529 | 9.80E-09 | 1.81E-07 |
| 3915 | LAMC1 | Laminin Subunit Gamma 1 | 1.412920452 | 8.95E-14 | 8.63E-12 |
| 83693 | HSDL1 | Hydroxysteroid Dehydrogenase Like 1 | 1.410013246 | 1.32E-18 | 4.89E-16 |
| 1114 | CHGB | Chromogranin B | 1.40901692 | 0.000612977 | 0.002584997 |
| 10512 | SEMA3C | Semaphorin 3C | 1.396890272 | 1.19E-05 | 8.43E-05 |
| 341640 | FREM2 | FRAS1 Related Extracellular Matrix 2 | 1.386805036 | 0.001130106 | 0.004378289 |
| 81035 | COLEC12 | Collectin Subfamily Member 12 | 1.38341188 | 1.41E-08 | 2.45E-07 |
| 9824 | ARHGAP11A | Rho GTPase Activating Protein 11A | 1.372960066 | 2.71E-07 | 3.09E-06 |
| 5335 | PLCG1 | Phospholipase C Gamma 1 | 1.36143934 | 1.13E-16 | 2.39E-14 |
| 1009 | CDH11 | Cadherin 11 | 1.344044727 | 0.00031949 | 0.00147272 |
| 128178 | EDARADD | EDAR Associated Death Domain | 1.337316446 | 2.31E-11 | 9.67E-10 |
| 55789 | DEPDC1B | DEP Domain Containing 1B | 1.33274055 | 1.62E-08 | 2.76E-07 |
| 200916 | RPL22L1 | Ribosomal Protein L22 Like 1 | 1.324942678 | 1.24E-07 | 1.59E-06 |
| 1278 | COL1A2 | Collagen Type I Alpha 2 Chain | 1.322444628 | 0.000678742 | 0.002820625 |
| 286827 | TRIM59 | Tripartite Motif Containing 59 | 1.321362731 | 3.51E-11 | 1.38E-09 |
| 81930 | KIF18A | Kinesin Family Member 18A | 1.313615383 | 8.76E-08 | 1.19E-06 |
| 1277 | COL1A1 | Collagen Type I Alpha 1 Chain | 1.309318848 | 0.00043241 | 0.001912217 |
| 6628 | SNRPB | Small Nuclear Ribonucleoprotein Polypeptides B And B1 | 1.308231607 | 1.98E-12 | 1.21E-10 |
| 79968 | WDR76 | WD Repeat Domain 76 | 1.306458708 | 1.89E-09 | 4.40E-08 |
| 10916 | MAGED2 | MAGE Family Member D2 | 1.298488751 | 1.01E-15 | 1.59E-13 |
| 1012 | CDH13 | Cadherin 13 | 1.297535823 | 7.25E-11 | 2.52E-09 |
| 6696 | SPP1 | Secreted Phosphoprotein 1 | 1.294622191 | 0.005091168 | 0.015901239 |
| 55771 | PRR11 | Proline Rich 11 | 1.290242999 | 1.03E-08 | 1.88E-07 |
| 4692 | NDN | Necdin, MAGE Family Member | 1.28306659 | 5.86E-10 | 1.58E-08 |
| 1021 | CDK6 | Cyclin Dependent Kinase 6 | 1.276795869 | 3.73E-13 | 2.97E-11 |
| 5603 | MAPK13 | Mitogen-Activated Protein Kinase 13 | 1.272749762 | 2.97E-11 | 1.20E-09 |
| 4436 | MSH2 | MutS Homolog 2 | 1.268386096 | 3.05E-11 | 1.22E-09 |
| 3838 | KPNA2 | Karyopherin Subunit Alpha 2 | 1.262954123 | 7.20E-08 | 1.01E-06 |
| 220134 | SKA1 | Spindle And Kinetochore Associated Complex Subunit 1 | 1.258371464 | 3.90E-09 | 8.22E-08 |
| 55227 | LRRC1 | Leucine Rich Repeat Containing 1 | 1.257251169 | 3.26E-15 | 4.58E-13 |
| 4176 | MCM7 | Minichromosome Maintenance Complex Component 7 | 1.255867325 | 5.20E-10 | 1.41E-08 |
| 53335 | BCL11A | BAF Chromatin Remodeling Complex Subunit BCL11A | 1.245420473 | 4.13E-06 | 3.36E-05 |
| 4605 | MYBL2 | MYB Proto-Oncogene Like 2 | 1.245419756 | 3.71E-06 | 3.06E-05 |
| 9735 | KNTC1 | Kinetochore Associated 1 | 1.240726417 | 7.23E-09 | 1.39E-07 |
| 1382 | CRABP2 | Cellular Retinoic Acid Binding Protein 2 | 1.232636903 | 0.00054436 | 0.00232797 |
| 253714 | MMS22L | MMS22 Like, DNA Repair Protein | 1.23222546 | 1.46E-09 | 3.53E-08 |
| 4174 | MCM5 | Minichromosome Maintenance Complex Component 5 | 1.229977963 | 5.00E-09 | 1.02E-07 |
| 6510 | SLC1A5 | Solute Carrier Family 1 Member 5 | 1.229878082 | 2.63E-06 | 2.29E-05 |
| 51195 | RAPGEFL1 | Rap Guanine Nucleotide Exchange Factor Like 1 | 1.227957869 | 4.35E-08 | 6.47E-07 |
| 477 | ATP1A2 | ATPase Na+/K+ Transporting Subunit Alpha 2 | 1.224111797 | 0.000705871 | 0.002917257 |
| 55635 | DEPDC1 | DEP Domain Containing 1 | 1.222154908 | 3.57E-06 | 2.97E-05 |
| 3766 | KCNJ10 | Potassium Inwardly Rectifying Channel Subfamily J Member 10 | 1.221235997 | 0.000173647 | 0.000870825 |
| 4171 | MCM2 | Minichromosome Maintenance Complex Component 2 | 1.219664012 | 5.87E-09 | 1.17E-07 |
| 57198 | ATP8B2 | ATPase Phospholipid Transporting 8B2 | 1.217890176 | 1.38E-10 | 4.40E-09 |
| 23171 | GPD1L | Glycerol-3-Phosphate Dehydrogenase 1 Like | 1.214785538 | 3.97E-06 | 3.25E-05 |
| 4173 | MCM4 | Minichromosome Maintenance Complex Component 4 | 1.212793479 | 1.93E-08 | 3.20E-07 |
| 1152 | CKB | Creatine Kinase B | 1.212793145 | 7.54E-08 | 1.05E-06 |
| 3833 | KIFC1 | Kinesin Family Member C1 | 1.209645729 | 1.50E-08 | 2.58E-07 |
| 3762 | KCNJ5 | Potassium Inwardly Rectifying Channel Subfamily J Member 5 | 1.205064071 | 3.18E-07 | 3.56E-06 |
| 80312 | TET1 | Tet Methylcytosine Dioxygenase 1 | 1.198754251 | 2.02E-10 | 6.19E-09 |
| 1019 | CDK4 | Cyclin Dependent Kinase 4 | 1.194233101 | 8.33E-12 | 4.09E-10 |
| 84798 | C19orf48 | Chromosome 19 Open Reading Frame 48 | 1.193477613 | 5.15E-07 | 5.47E-06 |
| 54908 | SPDL1 | Spindle Apparatus Coiled-Coil Protein 1 | 1.190062366 | 7.21E-08 | 1.01E-06 |
| 10155 | TRIM28 | Tripartite Motif Containing 28 | 1.182951146 | 4.06E-11 | 1.56E-09 |
| 619279 | ZNF704 | Zinc Finger Protein 704 | 1.165593036 | 2.02E-10 | 6.19E-09 |
| 79644 | SRD5A3 | Steroid 5 Alpha-Reductase 3 | 1.162481738 | 1.57E-12 | 9.85E-11 |
| 5832 | ALDH18A1 | Aldehyde Dehydrogenase 18 Family Member A1 | 1.162397337 | 1.95E-10 | 6.05E-09 |
| 5557 | PRIM1 | DNA Primase Subunit 1 | 1.160490681 | 2.26E-11 | 9.60E-10 |
| 10733 | PLK4 | Polo Like Kinase 4 | 1.159711359 | 2.43E-07 | 2.84E-06 |
| 10635 | RAD51AP1 | RAD51 Associated Protein 1 | 1.157837276 | 7.84E-08 | 1.08E-06 |
| 80727 | TTYH3 | Tweety Family Member 3 | 1.150129393 | 2.82E-09 | 6.27E-08 |
| 4673 | NAP1L1 | Nucleosome Assembly Protein 1 Like 1 | 1.149112145 | 8.81E-08 | 1.20E-06 |
| 4172 | MCM3 | Minichromosome Maintenance Complex Component 3 | 1.148280065 | 1.42E-11 | 6.48E-10 |
| 55084 | SOBP | Sine Oculis Binding Protein Homolog | 1.144253776 | 8.73E-11 | 2.96E-09 |
| 10403 | NDC80 | NDC80 Kinetochore Complex Component | 1.143425667 | 7.71E-08 | 1.07E-06 |
| 23462 | HEY1 | Hes Related Family BHLH Transcription Factor With YRPW Motif 1 | 1.141305957 | 1.27E-06 | 1.20E-05 |
| 55740 | ENAH | ENAH Actin Regulator | 1.139484218 | 1.38E-07 | 1.74E-06 |
| 47 | ACLY | ATP Citrate Lyase | 1.135436034 | 8.68E-13 | 6.12E-11 |
| 150468 | CKAP2L | Cytoskeleton Associated Protein 2 Like | 1.135155676 | 1.20E-06 | 1.15E-05 |
| 23468 | CBX5 | Chromobox 5 | 1.134068619 | 3.28E-08 | 5.06E-07 |
| 56992 | KIF15 | Kinesin Family Member 15 | 1.122828566 | 1.25E-06 | 1.19E-05 |
| 9203 | ZMYM3 | Zinc Finger MYM-Type Containing 3 | 1.119125701 | 8.21E-11 | 2.80E-09 |
| 7283 | TUBG1 | Tubulin Gamma 1 | 1.118529969 | 3.77E-11 | 1.46E-09 |
| 94234 | FOXQ1 | Forkhead Box Q1 | 1.114754756 | 2.61E-07 | 3.01E-06 |
| 113130 | CDCA5 | Cell Division Cycle Associated 5 | 1.112635096 | 1.29E-08 | 2.28E-07 |
| 1836 | SLC26A2 | Solute Carrier Family 26 Member 2 | 1.108743685 | 5.73E-11 | 2.07E-09 |
| 5983 | RFC3 | Replication Factor C Subunit 3 | 1.102139322 | 1.22E-10 | 3.95E-09 |
| 55662 | HIF1AN | Hypoxia Inducible Factor 1 Subunit Alpha Inhibitor | 1.100867652 | 3.77E-14 | 4.06E-12 |
| 116372 | LYPD1 | LY6/PLAUR Domain Containing 1 | 1.097867273 | 6.51E-05 | 0.000370991 |
| 55867 | SLC22A11 | Solute Carrier Family 22 Member 11 | 1.095875444 | 3.22E-06 | 2.72E-05 |
| 83990 | BRIP1 | BRCA1 Interacting Helicase 1 | 1.095512984 | 1.63E-06 | 1.50E-05 |
| 51203 | NUSAP1 | Nucleolar And Spindle Associated Protein 1 | 1.093642275 | 6.28E-08 | 8.93E-07 |
| 1282 | COL4A1 | Collagen Type IV Alpha 1 Chain | 1.092460999 | 4.59E-06 | 3.68E-05 |
| 5947 | RBP1 | Retinol Binding Protein 1 | 1.090691404 | 8.54E-06 | 6.30E-05 |
| 607 | BCL9 | BCL9 Transcription Coactivator | 1.090415746 | 8.41E-09 | 1.59E-07 |
| 25797 | QPCT | Glutaminyl-Peptide Cyclotransferase | 1.088459588 | 2.78E-07 | 3.16E-06 |
| 56944 | OLFML3 | Olfactomedin Like 3 | 1.086643451 | 0.000312586 | 0.001442664 |
| 7157 | TP53 | Tumor Protein P53 | 1.083074351 | 1.83E-08 | 3.05E-07 |
| 5326 | PLAGL2 | PLAG1 Like Zinc Finger 2 | 1.081327318 | 7.93E-10 | 2.05E-08 |
| 80020 | FOXRED2 | FAD Dependent Oxidoreductase Domain Containing 2 | 1.078138073 | 1.42E-08 | 2.46E-07 |
| 9493 | KIF23 | Kinesin Family Member 23 | 1.076823247 | 5.70E-08 | 8.22E-07 |
| 6502 | SKP2 | S-Phase Kinase Associated Protein 2 | 1.076306002 | 2.84E-10 | 8.34E-09 |
| 2941 | GSTA4 | Glutathione S-Transferase Alpha 4 | 1.0761372 | 2.49E-09 | 5.61E-08 |
| 23731 | TMEM245 | Transmembrane Protein 245 | 1.073459239 | 4.32E-10 | 1.19E-08 |
| 2222 | FDFT1 | Farnesyl-Diphosphate Farnesyltransferase 1 | 1.073323797 | 1.71E-08 | 2.89E-07 |
| 130827 | TMEM182 | Transmembrane Protein 182 | 1.072893011 | 3.95E-11 | 1.52E-09 |
| 84886 | C1orf198 | Chromosome 1 Open Reading Frame 198 | 1.070806306 | 3.23E-08 | 4.99E-07 |
| 50937 | CDON | Cell Adhesion Associated, Oncogene Regulated | 1.068972884 | 2.88E-10 | 8.45E-09 |
| 81618 | ITM2C | Integral Membrane Protein 2C | 1.068815532 | 7.42E-09 | 1.42E-07 |
| 23177 | CEP68 | Centrosomal Protein 68 | 1.068679968 | 3.91E-18 | 1.25E-15 |
| 11130 | ZWINT | ZW10 Interacting Kinetochore Protein | 1.06849927 | 7.54E-10 | 1.97E-08 |
| 10389 | SCML2 | Scm Polycomb Group Protein Like 2 | 1.066559831 | 2.43E-09 | 5.52E-08 |
| 79172 | CENPO | Centromere Protein O | 1.062204179 | 2.65E-08 | 4.21E-07 |
| 56122 | PCDHB14 | Protocadherin Beta 14 | 1.055205961 | 0.000127999 | 0.000669557 |
| 5955 | RCN2 | Reticulocalbin 2 | 1.050520455 | 2.11E-07 | 2.52E-06 |
| 1627 | DBN1 | Drebrin 1 | 1.049907114 | 2.16E-07 | 2.58E-06 |
| 9331 | B4GALT6 | Beta-1,4-Galactosyltransferase 6 | 1.048521957 | 5.48E-09 | 1.10E-07 |
| 27005 | USP21 | Ubiquitin Specific Peptidase 21 | 1.047938434 | 1.02E-09 | 2.58E-08 |
| 92370 | PXYLP1 | 2-Phosphoxylose Phosphatase 1 | 1.045977505 | 2.45E-09 | 5.55E-08 |
| 9156 | EXO1 | Exonuclease 1 | 1.038203482 | 1.90E-08 | 3.15E-07 |
| 55033 | FKBP14 | FKBP Prolyl Isomerase 14 | 1.03566954 | 3.96E-08 | 6.01E-07 |
| 57446 | NDRG3 | NDRG Family Member 3 | 1.034632045 | 5.73E-11 | 2.07E-09 |
| 5315 | PKM | Pyruvate Kinase M1/2 | 1.03173229 | 7.23E-07 | 7.33E-06 |
| 283431 | GAS2L3 | Growth Arrest Specific 2 Like 3 | 1.027556732 | 4.13E-07 | 4.50E-06 |
| 1363 | CPE | Carboxypeptidase E | 1.024788677 | 0.000886674 | 0.003549146 |
| 6532 | SLC6A4 | Solute Carrier Family 6 Member 4 | 1.022024726 | 1.74E-07 | 2.14E-06 |
| 6944 | VPS72 | Vacuolar Protein Sorting 72 Homolog | 1.020957624 | 3.66E-07 | 4.04E-06 |
| 871 | SERPINH1 | Serpin Family H Member 1 | 1.019139619 | 2.41E-07 | 2.83E-06 |
| 7260 | EIPR1 | EARP Complex And GARP Complex Interacting Protein 1 | 1.014197491 | 5.79E-09 | 1.15E-07 |
| 580 | BARD1 | BRCA1 Associated RING Domain 1 | 1.009222565 | 4.31E-07 | 4.67E-06 |
| 57216 | VANGL2 | VANGL Planar Cell Polarity Protein 2 | 1.005586922 | 4.28E-06 | 3.47E-05 |
| 4131 | MAP1B | Microtubule Associated Protein 1B | 1.002985048 | 6.30E-05 | 0.000359993 |
